# Parallel evolution of integrated craniofacial traits in trophic specialist pupfishes

**DOI:** 10.1002/ece3.11640

**Published:** 2024-07-07

**Authors:** Michelle E. St. John, Julia C. Dunker, Emilie J. Richards, Stephanie Romero, Christopher H. Martin

**Affiliations:** ^1^ Department of Biology University of Oklahoma Norman Oklahoma USA; ^2^ Department of Integrative Biology University of California Berkeley California USA; ^3^ Department of Ecology, Evolution and Behavior University of Minnesota Minneapolis Minnesota USA; ^4^ Department of Evolution and Ecology University of California Davis California USA; ^5^ Museum of Vertebrate Zoology University of California Berkeley California USA

**Keywords:** convergence, durophagy, ecological speciation, ecomorph, functional morphology, lepidophagy, parallel evolution, quantitative trait loci

## Abstract

Populations may adapt to similar environments via parallel or non‐parallel genetic changes, but the frequency of these alternative mechanisms and underlying contributing factors are still poorly understood outside model systems. We used QTL mapping to investigate the genetic basis of highly divergent craniofacial traits between the scale‐eater (*Cyprinodon desquamator*) and molluscivore (*C. brontotheroides*) pupfish adapting to two different hypersaline lake environments on San Salvador Island, Bahamas. We lab‐reared F2 scale‐eater x molluscivore intercrosses from two different lake populations, estimated linkage maps, scanned for significant QTL for 29 skeletal and craniofacial traits, female mate preference, and sex. We compared the location of QTL between lakes to quantify parallel and non‐parallel genetic changes. We detected significant QTL for six craniofacial traits in at least one lake. However, nearly all shared QTL loci were associated with a different craniofacial trait within each lake. Therefore, our estimate of parallel evolution of craniofacial genetic architecture could range from one out of six identical trait QTL (low parallelism) to five out of six integrated trait QTL (high parallelism). We suggest that pleiotropy and trait integration can affect estimates of parallel evolution, particularly within rapid radiations. We also observed increased adaptive introgression in shared QTL regions, suggesting that gene flow contributed to parallel evolution. Overall, our results suggest that the same genomic regions may contribute to parallel adaptation across integrated suites of craniofacial traits, rather than specific traits, and highlight the need for a more expansive definition of parallel evolution.

## Graphical abstract

Here, we mapped the genetic basis of 30 craniofacial and skeletal traits in two independent lake populations of novel trophic specialist pupfishes endemic to San Salvador Island in the Bahamas. These ecomorphs are scale‐eating and molluscivore specialists, respectively, with parallel phenotypes across several lake populations on the island. We found support for parallel genetic changes in both lakes for five out six significant QTL detected.

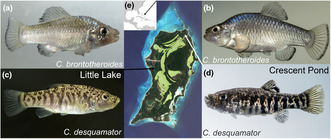

## 1 INTRODUCTION

Convergent evolution describes the independent evolution of similar phenotypes in response to similar selective pressures and provides strong support for ecological adaptation (Cerca, 2023). This includes both non‐parallel genetic changes, such as the evolution of antifreeze glycoproteins in icefishes or the “thunniform” body shape of lamnid sharks and tunas (Chen et al., 1997; Donley et al., 2004), and parallel genetic changes such as tetrodotoxin resistance in snakes and pufferfishes (Tarvin et al., 2023) or the evolution of voltage‐gated sodium channels in mormyrid and gymnotiform electric fishes (Feldman et al., 2009; Hopkins, 1995; Jost et al., 2008; Katz, 2006). Instances of convergence across independent lineages (i.e., across groups that lack a recent common ancestor and shared genetic backgrounds) provide the strongest evidence for adaptation; however, repeated evolution of similar phenotypes in response to similar selective pressures among lineages derived from the same ancestral population can also provide insight into the process of adaptation. Understanding this process, traditionally known as parallel evolution (Futuyman, 1986), is important because it can help to tease apart the contributions of natural selection and shared genetic constraints to similar phenotypes (Greenway et al., 2020; Schluter et al., 2004). Parallel phenotypic evolution can also occur via parallel or non‐parallel genetic changes (e.g., Chan et al., 2010; Cresko et al., 2004); however, non‐parallel genetic changes resulting in the same phenotype (e.g., Chan et al., 2010; Xie et al., 2019) provide weaker evidence for adaptation than convergence across independent lineages due their shared ancestral background. Despite substantial attention, the frequency and likelihood of parallel phenotypic evolution via parallel or non‐parallel genetic changes is still relatively unknown (Rosenblum et al., 2014; Stern, 2013; Stern & Orgogozo, 2008).

Many factors influence whether parallel phenotypic evolution in similar environments is produced by parallel or non‐parallel genetic mechanisms. First, recently diverged species exhibit increased probabilities of genetic parallelism when adapting to similar environments. Recently diverged taxa may inhabit similar environments more frequently or they may have similar genetic architecture, similar genetic variance–covariance matrices, or similar genetic backgrounds that produce similar epistatic interactions (Conte et al., 2012; Rosenblum et al., 2014). Second, any mechanism that allows the use of the same adaptive genetic mechanism should increase the likelihood of convergence via parallelism, including the availability of shared standing genetic variation and introgression (Rosenblum et al., 2014). For example, threespine sticklebacks colonized freshwater thousands of times and converged on similar phenotypes largely due to selection on an ancient shared pool of marine standing genetic variation (Feulner et al., 2013; Haenel et al., 2019; Jones et al., 2012; Nelson & Cresko, 2018); but see (Chan et al., 2010; Stuart et al., 2017). Similarly, increased adaptive introgression should also make genetic parallelism more likely (Grant et al., 2004; Hedrick, 2013; Morjan & Rieseberg, 2004; Taylor et al., 2020). Third, adaptive genetic variation with larger effect sizes and fewer pleiotropic effects should be reused more frequently across populations, particularly when a population is far from a new adaptive optimum (Linnen et al., 2013; Orr, 2006; Stern, 2013). Finally, de novo mutations, large mutational target sizes, and polygenic adaptive phenotypes are more likely to result in parallel phenotypic evolution via non‐parallel genetic pathways (Bolnick et al., 2018; Kowalko et al., 2013; Wittkopp et al., 2003); but see: (Chan et al., 2010; Colosimo et al., 2004; Xie et al., 2019).

Quantitative trait locus (QTL) mapping is often used to infer whether parallel or non‐parallel genetic changes underlie parallel phenotypes. However, many QTL studies only investigate a limited number of traits that are controlled by large effect loci, which may bias the literature toward supporting genetic parallelism (Conte et al., 2012). This bias may be exacerbated by the fact that in many QTL studies the genomic regions associated with a parallel phenotype are large, contain many genes, and their effects on phenotypic variance are overestimated in under‐powered studies (Beavis, 1998). These methodological and experimental limitations reduce confidence in the specific genomic regions associated with a parallel phenotype and, by extension, reduce confidence in whether parallel evolution was due to parallel or non‐parallel genetic changes. One possible solution is to compare the genomic regions associated with many different phenotypes across populations (Erickson et al., 2016). In this scenario, shared genomic regions across populations provide strong support for genetic parallelism, except in the likely rare instances of independent de novo mutations within the same region (O'Brown et al., 2015; Xie et al., 2019).

An adaptive radiation of pupfishes endemic to San Salvaodr Island (SSI) in the Bahamas is an excellent system for investigating the genetic underpinnings of parallel speciation because novel trophic specialists occur in sympatry across multiple hypersaline lake populations on the island (Hernandez et al., 2018; Martin et al., 2019; Martin & Wainwright, 2011, 2013b; Turner et al., 2008). The radiation includes three described pupfish species: a widespread generalist pupfish (*Cyprinodon variegatus*), an endemic scale‐eating (lepidophagous) pupfish (*C. desquamator*), and an endemic molluscivore (durophagous) pupfish (*C. brontotheroides*; Martin & Wainwright, 2013a). The molluscivore and scale‐eating pupfishes are endemic to SSI and occur in sympatry with one another and the generalist pupfish. There is also a fourth endemic species that occasionally feeds on scales, *C*. sp. “wide‐mouth,” not investigated here (Richards & Martin, 2021).

Trophic specialists show strong evidence of adaptive introgression among lakes, supporting their non‐independent origin (Martin & Feinstein, 2014; Patton et al., 2022; Richards et al., 2021), and have adapted to novel ecological niches among all Cyprinodontiform fishes (Martin & Wainwright, 2013c). Scale‐eaters across all lakes exhibit increased oral jaw size and reduced lower jaw angles during scale‐eating strikes which may play a critical role in scale‐biting performance during high‐speed strikes on their prey (St. John, Holzman, & Martin, 2020; Tan et al., 2023). Similarly, the snail‐eating pupfish exhibits a novel nasal protrusion which may improve oral snail‐shelling performance or result from sexual selection (Martin & Wainwright, 2013a; St. John, Dixon, & Martin, 2020). Furthermore, the nasal protrusion of the snail‐eating species varies substantially among lake populations (Hernandez et al., 2018; Martin & Feinstein, 2014). Despite the importance of these species‐specific traits, we still do not understand how their genetic architecture varies across lake populations.

There is some evidence to suggest that predominantly parallel genetic changes underlie specialist phenotypes on SSI. First, the SSI radiation is very young, diverging only about 10 kya (Hagey & Mylroie, 1995). Second, previous genomic analyses show that many of the alleles associated with trophic specialization arrived on SSI from Caribbean‐wide standing genetic variation within generalist pupfish populations, but there are also some de novo adaptive mutations associated with scale‐eating (Richards et al., 2021). Scale‐eaters form a monophyletic group, suggesting a shared genetic component to the scale‐eating phenotype across lakes (Martin & Feinstein, 2014; Richards & Martin, 2017). In contrast, molluscivores and generalists often genetically cluster together by lake instead of by species—suggesting that non‐parallel genetic changes could underlie parallel molluscivore phenotypes across lakes (Martin & Feinstein, 2014; Richards & Martin, 2017). Furthermore, previous studies have documented strong genetic divergence between scale‐eaters from Crescent Pond and all other populations of scale‐eater (McGirr & Martin, 2017, 2018b, 2019, 2020, 2021; Richards et al., 2021). Transcriptomic studies of developing embryos indicate both parallel and divergent differential gene expression in the specialists relative to the generalist species (McGirr & Martin, 2018a).

In this study, we compared QTL loci underlying 30 skeletal craniofacial and body traits in lab‐reared F2 intercrosses between trophic specialist pupfishes from Crescent Pond and Little Lake. Given the substantial similarity in trophic morphology of these dietary specialists across lakes on the island, particularly the scale‐eaters which are visibly indistinguishable between these two populations, and extensive evidence of gene flow and a shared genetic basis to these phenotypes across lakes (Martin & Feinstein, 2014; McGirr, 2020; Palominos et al., 2023; Richards et al., 2021; Richards & Martin, 2017), we predicted that the genetic architecture of trophic morphology would be highly parallel across lakes and driven by the same underlying adaptive loci shared among populations.

## 2 METHODS

### 2.1 Laboratory genetic cross

**FIGURE 1:**
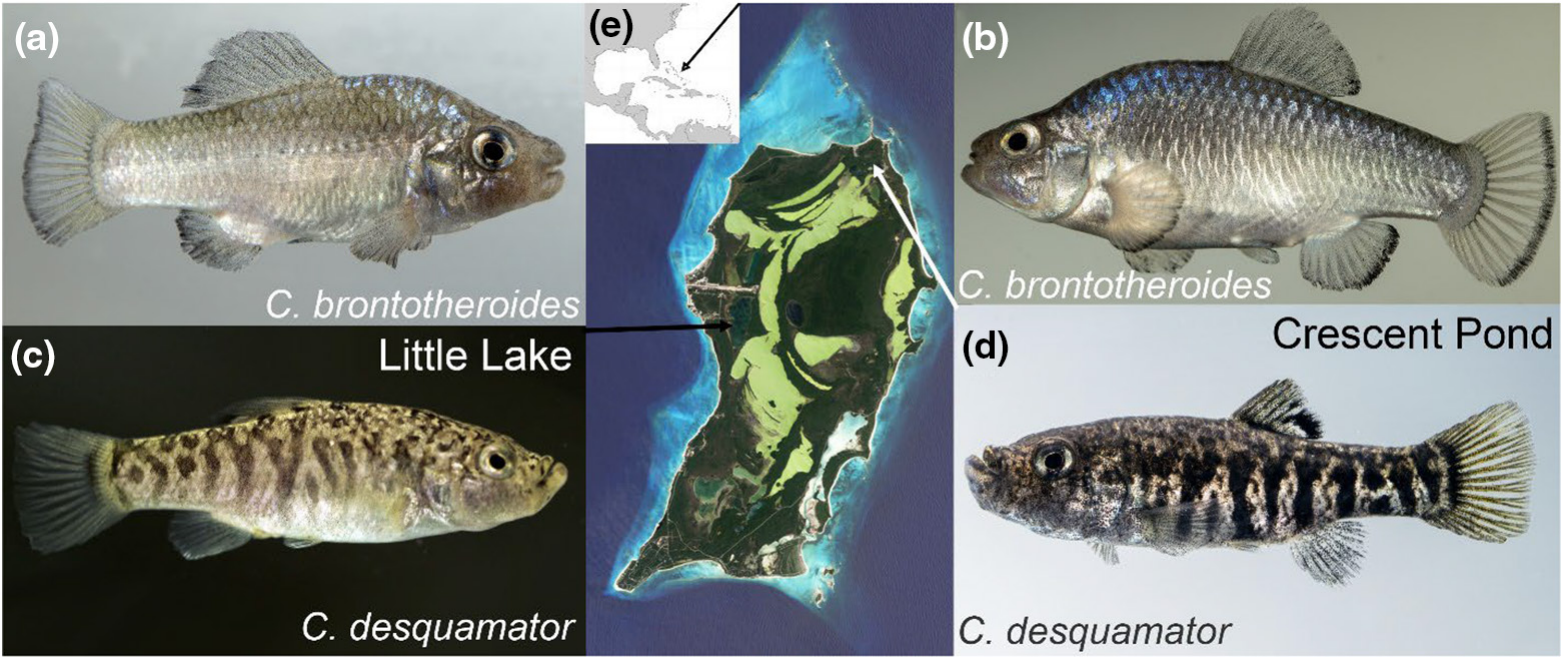
Two hybrid crosses from two lake populations on San Salvador Island, Bahamas. Lab‐reared molluscivore (*C. brontotheroides*) x scale‐eater (*C. desquamator*) crosses from Little Lake (a, c) and Crescent Pond (b, d). (e) Inset Caribbean map shows the location of San Salvador Island with collection sites for each cross indicated on the larger island map. Laboratory colonies were raised over multiple generations in a common garden laboratory environment, indicating that the differences in trophic morphology shown have a genetic basis. Representative photograph of Little Lake *C. desquamator* is from a closely connected lake population due to the lack of high resolution photographs of lab‐reared females from Little Lake.

We collected scale‐eating and molluscivore pupfishes from two different lake populations on San Salvador Island where generalist, molluscivore, and scale‐eater occur in sympatry: Crescent Pond and Little Lake, in 2011, 2013, 2014, and 2015 using seine nets or hand nets. We brought individuals back to the University of California, Davis, or the University of California, Berkeley, and a single wild‐caught scale‐eating female was allowed to breed freely with a single wild‐caught molluscivore male from the same lake resulting in two independent genetic crosses (Figure 1; one from each lake). At least four F1 offspring from each hybrid cross were crossed to produce an F2 intercross, resulting in 354 individuals from Crescent Pond and 287 individuals from Little Lake included in this study. All breeders and hybrids were maintained in 40‐L tanks at 5‐10 ppt salinity at the University of California, Davis, or the University of California, Berkeley, and reared in a common laboratory environment and common diet. We fed fry a diet of newly hatched *Artemia* nauplii for approximately 1 month post‐hatching, after which they were switched to the adult diet of frozen bloodworms and commercial pellet foods. We euthanized fish in an overdose of MS‐222 (Finquel, Inc.) according to the approved University of California, Davis Institutional Animal Care and Use Protocol #17455 or University of California, Berkeley IACUC Protocol AUP‐2015‐01‐7053, and stored specimens in 95%–100% ethanol.

### 2.2 Phenotyping: Sex and female mating preference

For individuals from Crescent Pond, we recorded their sex using their sexually dimorphic body and fin coloration. All species of male pupfish in this study develop blue iridescent coloration along their anterodorsal surface and a black marginal band along their caudal fin (Echelle & Echelle, 2020).

Once F2 hybrids reached sexual maturity, we performed mating assays using a subset of the hybrid females from Crescent Pond to estimate mating preferences for molluscivore or scale‐eating mates (*N* = 74). Prior to the mating assays, female fish were isolated for at least 12 hours and conditioned on frozen bloodworms with a 12:12 light: dark cycle. Mating assays occurred in three 1.1 m diameter kiddie pools (5–10 ppt salinity). Pools were covered with calcium carbonate gravel substrate approximating field conditions and divided in half. In each half, we placed three clear plastic 7.5‐L transparent containers in a row (Kritter Keepers brand), each containing a male housed individually to avoid aggression. Size‐matched adult scale‐eater males were placed on one side of each arena and molluscivore males on the other. Once the males were placed individually in clear boxes, a female F2 hybrid from Crescent Pond was introduced into the center of one of the three pools, chosen at random. We considered females acclimated to the pool once they had visited (within one body length) both rows of males, after which we started the seven‐minute trial period. During each trial, we recorded the amount of time a hybrid female spent on either the scale‐eater or molluscivore side of each divided pool. Each female was tested consecutively in all three pools, and we used the mean of her association time (scale‐eater association time / total association time during each 7‐minute trial) across all three pools for QTL analysis. Size‐matched males were periodically rotated into and rotated among kiddie pools during the approximately 12‐month testing period and the species on each side of the pool were periodically switched.

### 2.3 Phenotyping: Skeletal morphology

**FIGURE 2:**
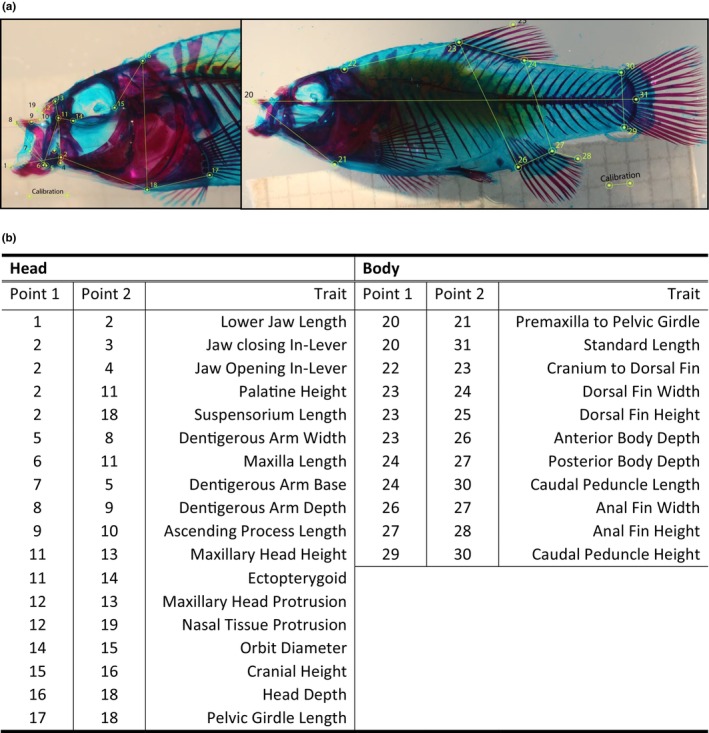
(a) Representative photographs of F2 intercross cleared and double‐stained specimen used for skeletal morphometrics. Points represent landmarks used to measure linear distances between skeletal traits. (b) Table containing the two landmarks that correspond to each trait.

To measure skeletal phenotypes in our F2 intercrosses, we cleared and double‐stained each specimen with alizarin red and alcian blue. Before clearing and staining, each fish was skinned and initially fixed in 95%–100% ethanol. We then placed specimens in 10% buffered formalin for at least 1 week and stained batches of individually labeled specimens following a modified version of Dingerkus and Uhler's (Dingerkus & Uhler, 1977) protocol. We suspended cleared and stained specimens in glycerin and photographed their left lateral side using a Canon EOS 60D digital SLR camera with a 60 mm macro lens. For each individual, we took two types of photographs: First, we took a whole‐body photograph to calculate fin and body measurements and second, a lateral skull image to calculate craniofacial measurements (Figure 2). We used DLTdv8 software (Hedrick, 2008) to digitize 11 landmarks on each whole‐body image and 19 landmarks on each lateral image following morphometric methods described in (Martin et al., 2017). For individuals from Crescent Pond, we also weighed the adductor mandibulae muscle mass. Each image included a standardized grid background which we used to calibrate and transform our measurements from pixels into millimeters. In total, we measured 354 individuals from Crescent Pond and 287 individuals from Little Lake. We used R to convert the 30 landmarks into linear distances. To reduce measurement error due to the lateral positioning of the specimens, we took the mean distances from the two clearest skull and whole‐body photographs for each individual when possible. If an individual did not have two clear photographs for each orientation, we measured the single clearest photograph. Finally, we size‐corrected each trait by using the residuals from a linear regression of the log‐transformed trait relative to log‐transformed standard length. We investigated whether size‐corrected traits varied between the two populations, but found no appreciable difference between them (Figure S1; MANOVA on PC1 and PC2, df = 28, approximate *F*‐value = 0.34, *p* = 1).

### 2.4 Genotyping

We genotyped hybrid individuals using reduced representation sequencing (ddRADseq and NextRAD), and we genotyped the original parents of each cross using whole‐genome sequencing. We used DNeasy Blood and Tissue Kits (Qiagen, Inc.) to extract DNA from the muscle tissue of each fish and quantified on a Qubit 3.0 fluorometer (Thermofisher Scientific, Inc.). Genomic libraries of the parents were prepared at the Vincent J. Coates Genomic Sequencing Center (QB3) using the automated Apollo 324 system (WaterGen Biosystems, Inc.). We used 150 paired‐end sequencing on an Illumina Hiseq4000 for these four parental samples.

For F2 hybrid individuals, we used a combination of reduced representation sequencing due to the serial nature of these laboratory crosses and our previously completed pilot QTL study of one F2 intercross from Crescent Pond (Martin et al., 2017). In addition to the 190 previously sequenced individuals from Crescent Pond used for this study, we included an additional 164 F2 individuals from Crescent Pond sequenced using double‐digest restriction site associated sequencing (ddRADseq) following similar library prep and sequencing methods described in (Martin et al., 2015, 2016, 2017). We prepared four indexed libraries each containing 96 barcoded individuals and sequenced these using 100 single‐end sequencing on two lanes of Illumina Hiseq4000 at the Vincent J. Coates Genomic Sequencing Center (QB3). We then sequenced all F2 individuals from Little Lake and resequenced a subset of Crescent Pond F2 low‐coverage individuals (*n* = 84), using NextRAD sequencing performed by SNPsaurus (SNPsaurus, LLC.) for library preparation and 150 single‐end sequencing on two lanes of Illumina Hiseq4000 at the University of Oregon sequencing core.

### 2.5 Calling variants

We called variants independently for each cross. First, we inspected raw read quality using FastQC (Babraham Bioinformatics Institute, v0.11.7) and trimmed reads to their appropriate length (100 bp for samples sequenced with ddRAD and 150 bp for samples sequenced with NextRAD) using TrimGalore! (v0.6.4). For samples that were sequenced using both ddRAD and NextRad methods, we concatenated trimmed raw reads into a single file. We next used bwa‐mem to map reads from all individuals in an intercross, both parents and offspring, to the *Cyprinodon brontotheroides* reference genome (v 1.0; total sequence length = 1,162,855,435 bp; number of scaffolds = 15,698, scaffold N50 = 32 Mbp; (Richards et al., 2021)). We identified duplicate reads using MarkDuplicates and created BAM indices using BuildBamIndex in the Picard package (http://picard.sourceforge.net (v.2.0.1)). Following the best practices guide from the Genome Analysis Toolkit (v 3.5; (Depristo et al., 2011)), we called and refined our single nucleotide polymorphism (SNP) variant data set using the program HaplotypeCaller. Pupfish lack high‐quality known variants because they are a non‐model organism; we therefore used the recommended hard filter criteria (QD < 2.0; FS < 60; MQRankSum < −12.5; ReadPosRankSum < −8; (Depristo et al., 2011; Marsden et al., 2014)) to filter our SNP variant dataset. Ultimately, we detected 13.7 million variants in our Crescent Pond dataset and 14.4 million variants in our Little Lake dataset.

We used the program STACKS (Catchen et al., 2013) to further filter our dataset and convert our vcf files into phenotype and genotype comma‐separated values files that could be imported into the Rqtl program in R. Specifically, we used the populations program to filter out variants that were not present in both the parental and F2 hybrid populations, and to filter out variants found in 10% or less of the population. From this filtering step, we retained 36,318 variants with 46.5 mean mappable F2 hybrids per site in Crescent Pond and 87,579 variants with 85.984 mean mappable F2 hybrids per site in Little Lake.

We further refined this dataset using the Rqtl (v1.46–2), and ASMap (v1.0–4) packages (Broman et al., 2003; Taylor & Butler, 2017). We started removed individuals that did not contain any filtered variants and any duplicate individuals. This reduced our Crescent Pond data set to 227 individuals, and our Little Lake data set to 281 individuals. Next, we filtered markers that had >0.98 or < 0.1 heterozygosity (Crescent Pond: markers = 15,247, Little Lake: markers = 14,661). This step also filtered out 13 individuals from Crescent Pond which only contained markers with >0.98 or <0.1 heterozygosity. Before constructing our genetic maps, we set aside markers that appeared to suffer from segregation distortion. We used the pullCross() function from the ASmap package to exclude markers in both data sets that were missing in >75% of individuals, departed from Mendelian ratios (1:2:1), or contained any co‐located markers for the initial construction of the linkage maps. This filtering retained more than twice the number of markers for Crescent Pond than Little Lake. We therefore used a stricter filtering threshold for missing data (i.e., removing markers with >72% missing data) for our Crescent Pond dataset to construct linkage maps of comparable sizes for downstream comparative analyses. At the end of this filtering process, the Crescent Pond dataset contained 214 individuals and 657 SNP markers and the Little Lake dataset contained 281 individuals with 490 SNP markers.

### 2.6 Linkage map construction

We used the mstmap.cross() function to form initial linkage groups and order markers, using the kosambi method for calculating genetic distances and a clustering threshold of *p* = 1 × 10^−14^ for Little Lake and *p* = 1 × 10^−20^ for Crescent Pond. After forming these initial linkage groups, we used the pushCross() function from the ASmap package to integrate previously set aside markers back into our map. We pushed markers back based on a segregation ratio of 3:4:3 and we pushed back any markers that had previously been designated as co‐located. This increased our map sizes to 817 markers for Crescent Pond and 580 markers for Little Lake. With these additional markers, we re‐estimated our linkage map using the est.rf() and formLinkageGroups() functions from the Rqtl package. We used a max recombination fraction of 0.35 and a minimum LOD threshold of 5 to estimate linkage groups for both data sets. We used the droponemarker() command from Rqtl with an error probability of .01 to identify and drop problematic markers from the genetic maps, including dropping linkage groups with three or fewer markers. Finally, we visually inspected our linkage groups using plotRF() from the Rqtl package and merged linkage groups which had been incorrectly split up using the mergeCross() function from the ASmap package. Ultimately, our final genetic maps included (1) Crescent Pond: 214 individuals, 743 markers, 24 linkage groups and (2) Little Lake: 281 individuals, 540 markers, and 24 linkage groups (Figure 2).

### 2.7 QTL analyses

We mapped QTL for 29 skeletal traits for both populations, and additional morphological (adductor mandibulae muscle mass) and behavioral traits (mate preference) for Crescent Pond. We used the Rqtl2 package (v0.22–11) to calculate genotype probabilities with a multipoint hidden Markov model using an error probability of 0.0001 and a Kosambi map function. We calculated kinship matrices to account for the relationship among individuals in two ways: (1) overall kinship, which represents the proportion of shared alleles between individuals, and (2) kinship calculated using the leave‐one‐chromosome‐out method (LOCO). We used the scan1() function to perform three separate genome scans using a single‐qtl model by (1) Haley–Knott regression, (2) a linear mixed model using the overall kinship matrix, and (3) a linear mixed model using the LOCO kinship matrix. For our Crescent Pond data set, we also included sex as an additive covariate. We assessed the significance of all three models using two significance thresholds *P* < .1 and *P* < .05 based on 1000 permutations each, using the scan1perm() function. As noted above, the scan1() function can use several different methods to determine if a region is significantly associated with a given phenotype (Broman et al., 2019; Haley & Knott, 1992; Yang et al., 2014; Yu et al., 2006); however, it is clear from previous theoretical work that many of these methods may suffer from type II error depending on the size of an organism's genome, the density of markers in a linkage map, or the complexity of the phenotypic traits being measured (Lander & Botstein, 1989; Risch, 1990). We therefore relaxed the *p*‐value cutoff from .05 to .1 to capture potentially important genomic regions. This relaxation is further supported by the LOD scores associated with regions significant at the *p* < .1 level because they all exceed the traditional threshold of 3 (Nyholt, 2000), the more conservative threshold of ~3.3 (Lander & Kruglyak, 1995; Nyholt, 2000), the suggestive threshold of 1.86 (Lander & Kruglyak, 1995), and are in line with estimates of significant LOD thresholds in previous studies (Erickson et al., 2016). All three of these methods detected similar QTLs and moving forward we only used the Haley–Knott regression method.

For each trait, we calculated the location of the maximum LOD score and used the fit1() function to re‐fit a single‐QTL model at this location. We used the newly calculated LOD score to estimate the proportion of variance explained by the QTL and to calculate a *p*‐value associated with each significant QTL (𝑥^2^ test). We also used the location of the maximum LOD score to calculate 95% Bayes credible intervals using the bayes_int() function from the Rqtl2 package. We note that the maximum LOD score associated with every trait across both ponds exceeded the suggestive threshold of 1.86 (Lander & Kruglyak, 1995). We used the find.markerpos() function from Rqtl to determine where markers in each linkage map fell within the reference genome. With this information, we were able to determine the scaffolds/positions from the reference genome that fell within the 95% credible intervals for each putative QTL. Finally, we used the maxmarg() function from the Rqtl2 package to find the genotype with the maximum marginal probability at the location of the maximum LOD. We used these genotypes to visualize the relationship between genotype and phenotypes.

### 2.8 Identifying adaptive alleles within QTL regions

For each scaffold that fell within a QTL's 95% credible interval, we calculated the minimum and maximum position for that scaffold (that was identified in the putative QTL region) and searched the *C. brontotheroides* reference genome for annotated genes within the region. We then compared this list to a previously published list of genes that (1) contained or were adjacent to (within 20 kbp) fixed or nearly fixed (*F*st > 0.95) SNPs between specialist species on SSI, and (2) showed significant evidence of a hard selective sweep in both the site frequency spectrum‐based method SweeD (Pavlidis et al., 2013) and the linkage‐disequilibrium‐based method OmegaPlus (Alachiotis et al., 2012). We hereafter refer to these loci as adaptive loci. We also noted whether adaptive loci within QTL regions were classified as de novo, introgressed, or as standing genetic variation on SSI (Richards et al., 2021). We used a bootstrap resampling method to determine whether the observed proportions of adaptive alleles originating from de novo, introgression, or standing genetic variation found within QTL 95% credible intervals were different than the proportions expected when drawn from the genome at random. We used the boot (v. 1.3–25) package (Buckland et al., 1998; Canty & Ripley, 2021) to resample our entire adaptive loci dataset (with replacement) 10,000 times. We then used the boot.ci() command from the boot package to calculated the 95% credible intervals around expected proportions of de novo, introgressed, and standing adaptive loci. We performed these calculations separately for scale‐eater and molluscivore adaptive loci.

## 3 RESULTS

### 3.1 Linkage map construction

**FIGURE 3:**
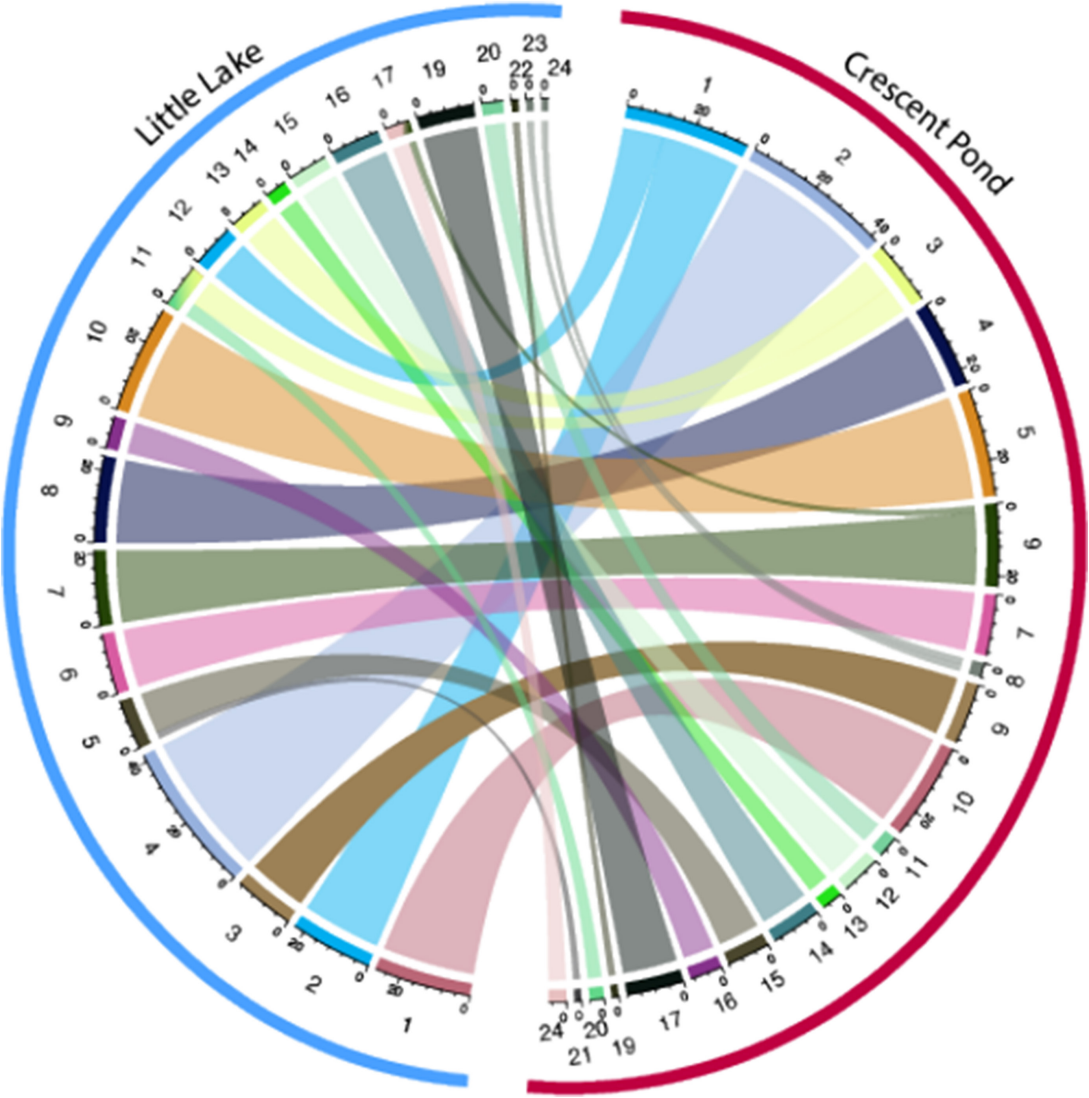
Circos plot depicting the relationship between the Crescent Pond (red) and Little Lake linkage maps (blue), which share 324 markers within 10 kbp of one another. Numbers surrounding each semi‐circle represent linkage group numbers in each lake. Markers that are shared across lakes are connected via the colored

We identified 24 linkage groups from 743 markers for Crescent Pond and 24 linkage groups from 540 markers for Little Lake (Figure S2). Previous karyotypes of *Cyprinodon* species estimated 24 diploid chromosomes, matching the linkage groups in this study (Liu & Echelle, 2013; Stevenson, 1981). The total map length for Crescent Pond was 7335 cM, and the total map length for Little Lake was 5330; the largest linkage groups for each map were 740 and 380 cM, respectively, and inter‐marker map distance did not exceed 20 cM in either map. To compare our maps and to determine if the same genomic regions were being reused across lakes, we identified where each marker was located in our reference genome. Overall, we found 324 markers in both maps that were within 10 Kbp of one another, indicating that 60% of the Little Lake map was also present in the Crescent Pond map and 44% of the Crescent Pond map was present in the Little Lake map (Figure 3).

### 3.2 Craniofacial QTL

We detected three significant craniofacial QTL in Crescent Pond and five significant craniofacial QTL in Little Lake (Table 1; Table 2). In Crescent Pond, we identified QTL associated with the depth of the dentigerous arm of the premaxilla, cranial height, and adductor mandibulae muscle mass. Cranial height in Crescent Pond mapped to linkage group (LG) 10. Dentigerous arm depth and adductor mandibulae muscle mass both mapped to LG 13, which also contained the max LOD scores for two additional jaw traits (jaw opening in‐lever and maxillary head height; Table 2). The 95% credible intervals for all these traits overlapped, suggesting that LG 13 may contain a single pleiotropic locus or many loci that affect all four traits.

**TABLE 1:** Maximum LOD scores for all 29 traits measured in Little Lake and Crescent Pond mapping crosses.

Trait	Population	Scaffold	Max LOD	Genome‐wide significance	*n*	PVE	x^2^ *p*‐value
Lower Jaw Length	Crescent Pond	53, 7087, 2336, 6275, 26, 7335	2.89		205	6.29	.0013
	Little Lake	24, 4028, 58, 16	3.30		228	6.45	.0005
Jaw closing In‐Lever	Crescent Pond	31, 4, 451, 19	3.60		204	7.81	.0002
	Little Lake	8, 9588, 8020	4.11	·	227	7.99	.0001
Jaw Opening In‐Lever	Crescent Pond	6086, 11	2.43		205	5.32	.0037
	Little Lake	43	2.98		227	5.87	.0010
Palatine Height	Crescent Pond	34, 22, 6304	2.90		205	6.31	.0013
	Little Lake	11	2.73		228	5.36	.0019
Suspensorium Length	Crescent Pond	46, 37, 31, 26, 60, 7556, 10,198, 22	3.54		204	7.68	.0003
	Little Lake	11	3.51		227	6.88	.0003
Dentigerous Arm Width	Crescent Pond	52, 13,137, 40	2.19		202	4.87	.0065
	Little Lake	24, 4028, 58, 16	4.05	·*	228	7.85	.0001
Maxilla Length	Crescent Pond	27, 593, 4, 31, 451, 19	2.67		204	5.85	.0021
	Little Lake	56	3.03		228	5.94	.0009
Dentigerous Arm Base	Crescent Pond	27, 593, 4, 31, 451, 19	2.98		205	6.47	.0011
	Little Lake	26	3.70		228	7.21	.0002
Dentigerous Arm Depth	Crescent Pond	6086, 11, 46	4.20	·*	205	9.00	.0001
	Little Lake	5	3.70	·	217	7.55	.0002
Ascending Process Length	Crescent Pond	27, 593, 4, 31, 451, 19	2.70		201	6.00	.0020
	Little Lake	46, 37	3.70		210	7.79	.0002
Maxillary Head Height	Crescent Pond	6086, 11, 46	2.33		205	5.11	.0046
	Little Lake	7, 30	2.19		228	4.33	.0064
Ectopterygoid	Crescent Pond	14, 9, 16, 5405, 11,419	2.81		205	6.11	.0016
	Little Lake	9	3.36		228	6.56	.0004
Maxillary Head Protrusion	Crescent Pond	58, 24, 41, 47	2.70		205	5.88	.0020
	Little Lake	7431, 53, 6275, 2336, 25	4.03	· *	228	7.82	.0001
Nasal Tissue Protrusion	Crescent Pond	46, 37, 31, 26, 60, 7556, 10,198, 22	2.25		205	4.93	.0056
	Little Lake	9	3.69		228	7.18	.0002
Orbit Diameter	Crescent Pond	9588, 8, 8020	2.34		205	5.13	.0045
	Little Lake	52, 40, 41	2.58		227	5.10	.0026
Cranial Height	Crescent Pond	33, 39	3.59	·	205	7.74	.0003
	Little Lake	33	3.94	·	224	7.78	.0001
Head Depth	Crescent Pond	16, 40	2.98		204	6.51	.0010
	Little Lake	52, 40, 41	2.71		223	5.45	.0019
Pelvic Girdle Length	Crescent Pond	31, 18, 15, 11,057, 55, 52	2.68		203	5.90	.0021
	Little Lake	27, 37, 7	2.87		226	5.68	.0014
Premaxilla Pelvic Girdle	Crescent Pond	37, 46, 7556, 10,198	3.15		202	6.92	.0007
	Little Lake	35, 38, 20, 8508, 10,278, 33	2.63		231	5.10	.0024
Standard Length (mm)	Crescent Pond	14, 9, 16, 5405, 11,419	2.90		204	6.34	.0013
	Little Lake	31, 46, 37	3.48		231	6.69	.0003
Cranium Dorsal Fin	Crescent Pond	6704, 52, 13,137, 40	2.84		205	6.18	.0014
	Little Lake	37, 22, 7556	3.45		231	6.65	.0004
Dorsal Fin Width	Crescent Pond	43, 26, 14,743	2.18		205	4.78	.0066
	Little Lake	842, 44, 1074, 6, 30	3.00		230	5.83	.0010
Dorsal Fin Height	Crescent Pond	18, 31, 15, 11,057, 55	2.84		203	6.23	.0015
	Little Lake	43	3.50		222	7.00	.0003
Anterior Body Depth	Crescent Pond	8, 8020	2.94		204	6.43	.0011
	Little Lake	6094, 5, 4	3.33		230	6.45	.0005
Posterior Body Depth	Crescent Pond	20, 471, 39, 8508, 33	2.86		203	6.27	.0014
	Little Lake	18, 15	3.02		228	5.92	.0009
Caudal Peduncle Length	Crescent Pond	31, 18, 15, 11,057, 55, 52	2.87		203	6.30	.0014
	Little Lake	24, 4028, 58, 16	2.16		230	4.23	.0070
Anal Fin Width	Crescent Pond	18, 15, 11,057, 55	2.89		201	6.41	.0013
	Little Lake	6, 842, 44, 1074, 30	2.40		229	4.71	.0040
Anal Fin Height	Crescent Pond	43, 26, 14,743	2.93		201	6.48	.0012
	Little Lake	8, 9588, 8020	3.15		229	6.14	.0007
Caudal Peduncle Height	Crescent Pond	53, 7087, 2336, 6275, 26, 7335	1.97		205	4.32	.0108
	Little Lake	47, 1962	3.32		230	6.44	.0005
Adductor	Crescent Pond	6086, 11	3.56	·	170	9.18	.0003
	Little Lake	‐	‐	‐	‐	‐	‐
Proportion Time Spent Near Scale‐Eater Mates	Crescent Pond	58, 24, 41, 47	2.05		74	12.00	.0089
	Little Lake	‐	‐	‐	‐	‐	‐

*Note*: A genome scan with a single‐QTL model by Haley–Knott regression was used to identify the position with the highest LOD score, 95% Bayesian credible intervals, and the genome‐wide significance level for each trait (*p* < .1: ·; *p* < .05: *). We also report the scaffold numbers of genomic regions that fell within the 95% credible intervals associated with the maximum LOD position for each trait, the number of individuals phenotyped, percent variance explained (PVE) by the max LOD region, and the uncorrected *p*‐value associated with each max LOD region.

**TABLE 2:** Position of maximum LOD score and 95% credible intervals for each trait in the Little Lake linkage map and the Crescent Pond linkage map.

Little Lake						Crescent pond					
	Trait	Sig.	LG	Position genome‐wide max LOD score	95% CI		Trait	Sig.	LG	Position genome‐wide max LOD score	95% CI
	Cranial Height	*	1	259	(250, 270)		Suspensorium Length		1	566	(20, 730)
	Premaxilla to Pelvic Girdle		1	146	(0, 350)		Nasal Tissue Protrusion		1	570	(0, 740)
	Cranium to Dorsal Fin		2	303	(160, 380)		Premaxilla to Pelvic Girdle		1	568	(310, 600)
	Lower Jaw Length		3	9	(0, 340)		Ectopterygoid		3	272	(0, 560)
	Dentigerous Arm Width	*	3	9	(0, 340)		Standard Length (mm)		3	50	(40, 500)
	Caudal Peduncle Length		3	168	(0, 340)		Dentigerous Arm Width		4	317	(40, 510)
	Dorsal Find width		4	187	(10, 310)		Cranium to Dorsal Fin		4	89	(30, 510)
	Anal Fin Width		4	14	(0, 280)		Lower Jaw Length		5	136	(0, 470)
	Dentigerous Arm Depth	*	6	79	(20, 90)		Caudal Peduncle height		5	381	(0, 470)
	Anterior Body Depth		6	289	(0, 300)		Jaw Closing In‐Lever		6	380	(150, 410)
	Orbit Diameter		8	266	(0, 290)		Maxilla Length		6	468	(0, 480)
	Head Depth		8	206	(0, 290)		Dentigerous Arm Base		6	107	(0, 480)
	Jaw Closing In‐Lever	*	9	54	(40, 90)		Ascending Process Length		6	106	(0, 470)
	Anal Fin Height		9	100	(70, 240)		Pelvic Girdle Length		8	370	(20, 435)
	Maxillary Head Protrusion	*	10	35	(0, 260)		Dorsal Fin Height		8	91	(0, 380)
	Ascending Process Length		12	119	(90, 150)		Caudal Peduncle Length		8	258	(30, 425)
	Standard Length (mm)		12	200	(50, 210)		Anal Fin Width		8	190	(110, 400)
	Ectopterygoid		13	170	(150, 180)		Maxillary Head Protrusion		9	300	(0, 350)
	Nasal Tissue Protrusion		13	193	(20, 200)		Proportion Time Spent Near Scale‐Eater Males		9	166	(50, 340)
	Palatine Height		14	147	(110, 210)		Cranial Height	*	10	204	(130, 340)
	Suspensorium Length		14	153	(70, 180)		Posterior Body Depth		10	270	(0, 330)
	Jaw Opening In‐Lever		16	58	(40, 140)		Palatine Height		11	70	(0, 310)
	Dorsal Fin Height		16	52	(40, 60)		Head Depth		12	111	(100, 280)
	Pelvic Girdle Length		17	50	(10, 160)		Opening In‐Lever		13	10	(0, 90)
	Maxillary Head Height		18	122	(30, 160)		Dentigerous Arm Depth	*	13	2	(0, 250)
	Caudal Peduncle Height		19	44	(20, 90)		Maxillary Head Height		13	170	(0, 280)
	Dentigerous Arm Base		21	74	(0, 100)		Adductor Mandibulae Mass	*	13	2	(0, 70)
	Maxilla Length		22	40	(20, 50)		Dorsal Fin Width		14	305	(30, 330)
	Posterior Body Depth		24	30	(10, 30)		Anal Fin Width		14	330	(280, 330)
							Orbit Diameter		16	107	(0, 190)
							Anterior Body Depth		16	170	(10, 220)

*Note*: Colors represent corresponding linkage groups across lakes. Asterisks represent traits that were marginally significant at the *p* < .1 level in the genome scan.

In Little Lake, we detected significant QTL associated with jaw closing in‐lever (i.e., height of the coronoid process on the articular: LG9), width and depth of the dentigerous arm of the premaxilla (LG3 and LG6), maxillary head protrusion (LG10), and cranial height (LG1; Tables 1 and 2). The 95% credible interval for dentigerous arm width on LG3 also contained the max LOD score for lower jaw length, suggesting that either a single pleiotropic locus or a cluster of loci in this region may be controlling both traits.

### 3.3 Candidate genes and adaptive loci within QTL regions

#### 3.3.1 Cranial height: Parallel QTL in both lakes

**FIGURE 4:**
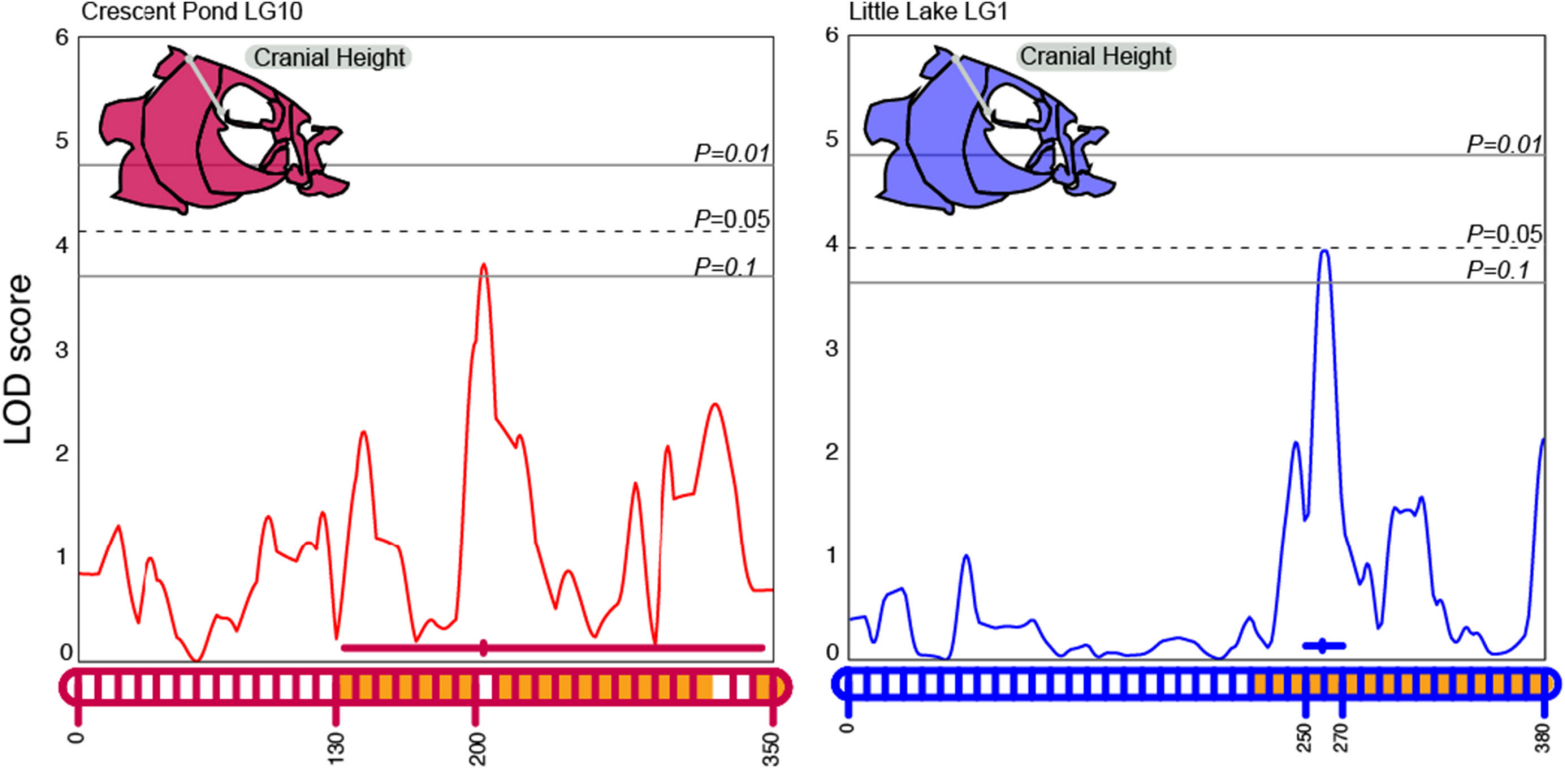
LOD profile for cranial height in Crescent Pond (red) and Little Lake (blue) F2 hybrids. LOD profiles were estimated by a Haley–Knott regression and are plotted relative to the position along the implicated linkage group (LG 10 for Crescent Pond, LG 1 for Little Lake) which are represented along the X‐axis. Genome‐wide significance levels of *p* = .1, .05, and .01 are shown by the gray horizontal lines. Linkage groups along the X‐axis also show the position of maximum LOD along with 95% Bayesian credible intervals. The orange fill color within the linkage groups corresponds to overlapping regions of scaffold 33 between crosses.

**FIGURE 5:**
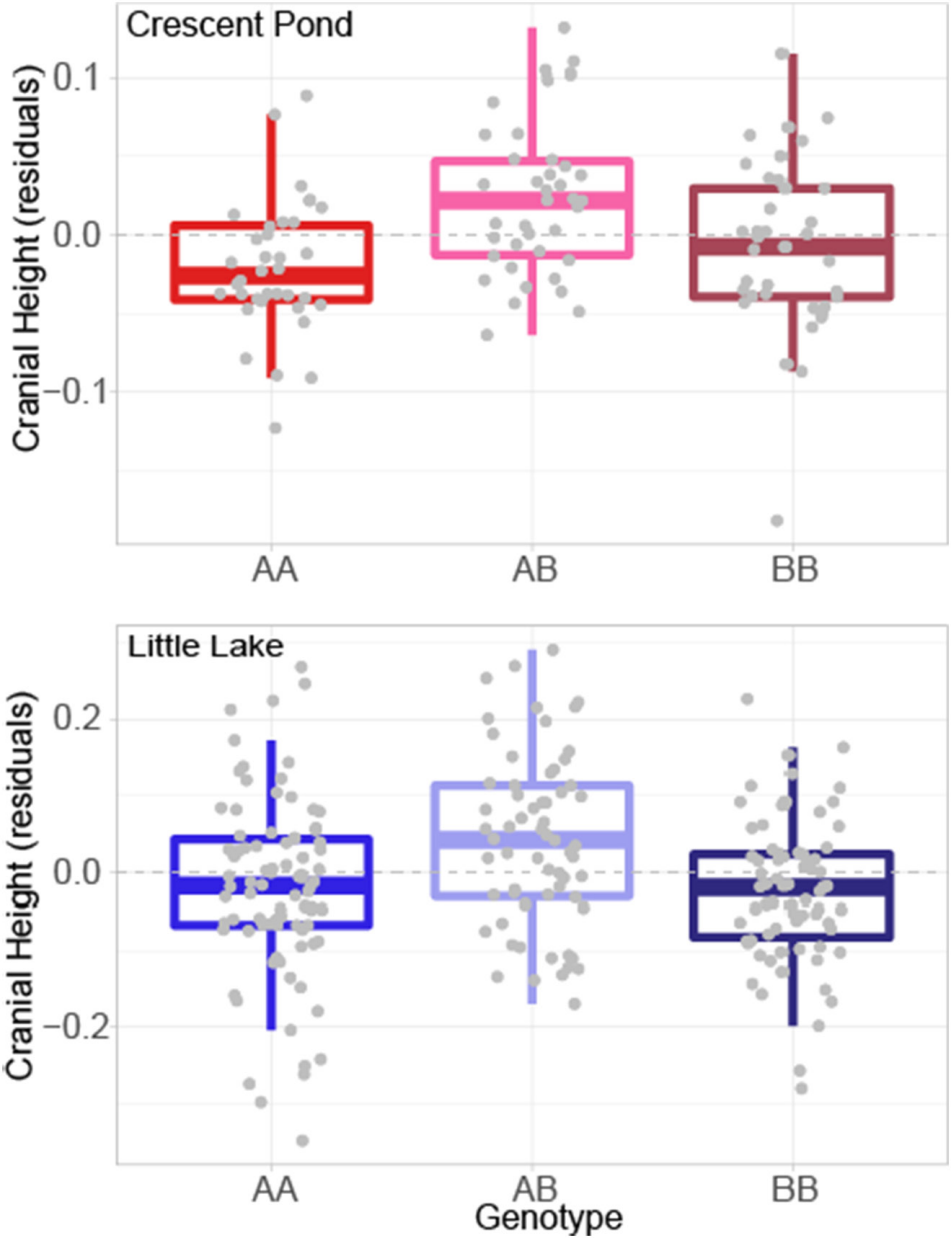
Cranial height phenotypes (size‐corrected residuals) for each genotype in Crescent Pond (red) and Little Lake (blue). Both lakes show that heterozygotes (AB) exhibit slighter greater cranial heights than homozygous genotypes, consistent with overdominance. Boxes indicate median, first and third quartile, and interquartile range.

Cranial height was the only trait with statistically significant or marginally significant QTL in both lakes (Figure 4, *p* < .1). While the QTL occurred on different linkage groups between maps, we found a high degree of synteny between these linkage groups indicating that the QTL is located in the same genomic region in both lakes (Table 2; Figure 3). We also found the same pattern of genetic architecture consistent with overdominance in both lake crosses: Heterozygotes showed slightly greater cranial height relative to homozygous individuals (Figure 5).

We found 44 genes within scaffold 33 that fell partially or fully within the 95% credible intervals of the QTL in both lakes (Table 1; Table S1). Only three of these genes showed evidence of hard selective sweeps within 20 kb: *wdr31*, *bri3bp*, and *gnaq* (Table 3). G*naq* is well known to be associated with craniofacial development and Sturge–Weber syndrome in humans (Hall et al., 2007; Martins et al., 2017; Shirley et al., 2013) and is differentially expressed between our specialist species in developing larvae (McGirr & Martin, 2020).

**TABLE 3:** Number of adaptive alleles and any genes within 20 kbp found in trait QTL with maximum LOD scores for both lakes.

Traits	Gene	Molluscivore		Scale‐eater		
		SGV	Intro.	SGV	Intro.	de novo
Cranial Height*	*bri3bp*	‐	26	28	‐	‐
	*gnaq*	9	‐	9	‐	‐
	*wdr31*	18	2	20	‐	‐
	Unannotated Regions	1	‐	11	‐	‐
Dentigerous Arm Width* Female mate preference^†^ Maxillary Head Protrusion^†^	*cyp26b1*	‐	8	8	‐	‐
	*dysf*	‐	‐	1	‐	‐
	Unannotated Regions	‐	67	216	‐	1
Dentigerous Arm Depth*	Unannotated Regions	‐	‐	1	‐	‐
Maxillary Head Protrusion* Lower Jaw Length^†^ Caudal Peduncle Height^†^	*cox6b1*	8	‐	8	‐	‐
	*cyp21a2*	‐	‐	2	‐	‐
	*eva1b*	‐	‐	2	‐	‐
	*fhod3*	‐	‐	2	‐	‐
	*galnt1*	‐	‐	‐	17	‐
	*glipr2*	‐	‐	3	‐	‐
	*hdac9b*	‐	‐	‐	1	‐
	*mag*	‐	‐	2	‐	‐
	*map7d1*	25	‐	25	‐	‐
	*mindy3*	‐	‐	8	‐	‐
	*nacad*	‐	‐	2	‐	‐
	*pxn1*	‐	‐	1	‐	‐
	*rasip1*	13	‐	13	‐	‐
	*slc2a3*	15	‐	15	‐	‐
	*steap4*	‐	‐	‐	26	‐
	*tbrg4*	‐	‐	2	‐	‐
	*them4*	‐	‐	5	‐	‐
	*tnc*	‐	‐	1	‐	‐
	*twist1*	‐	‐	‐	‐	1
	*zhx2*	5	‐	6	‐	‐
	*znf628*	5	‐	6	‐	‐
	Unannotated Regions	29	68	93	64	‐
Jaw closing In‐Lever* Orbit Diameter^†^ Anterior Body Depth^†^	*galr2*	‐	‐	‐	2	‐
	*map2k6*	‐	‐	‐	3	‐
Dentigerous Arm Depth* Adductor Mandibulae Masst* Palatine Height^†^ Suspensorium Length^†^	*atp8a2*	92	‐	92	‐	‐
	*cd226*	6	‐	6	‐	1
	*cdk8*	‐	‐	1	‐	‐
	*cmbl*	‐	‐	4	‐	7
	*crispld1*	‐	‐	7	‐	‐
	*dok6*	‐	‐	50	‐	‐
	*fbxl7*	‐	‐	6	‐	‐
	*hnf4g*	‐	‐	1	‐	‐
	*med1*	‐	‐	26	‐	‐
	*mtrr*	‐	‐	2	‐	‐
	*ncoa2*	7	‐	‐	4	‐
	*prlh*	‐	‐	12	6	‐
	*rnf6*	‐	‐	4	‐	‐

	*shisa2*	18	‐	38	‐	‐
	*slc51a*	‐	‐	22	‐	7
	*spice1*	4	‐	2	‐	‐
	*ube2w*	‐	48	‐	‐	‐
	*zfhx4*	‐	‐	‐	‐	1
Unannotated Regions	34	34	131	3	1

*Note*: Adaptive alleles were categorized as either standing genetic variation (SGV), introgression (Intro.), or de novo mutations, and were estimated independently for molluscivores and scale‐eaters in a previous study (Richards et al., 2021). Asterisks represent traits that were significant at the *p* < .1 level in the genome‐wide scan, while crosses show traits that corresponded to the same locations in the alternate lake.

#### 3.3.2 Dentigerous arm width: Parallel QTL for a correlated craniofacial trait

We found that regions on scaffolds 58 and 24 were associated with a significant QTL for dentigerous arm width in Little Lake and also contained the max LOD scores for maxillary head protrusion and female mate preference in Crescent Pond (Tables 1 and 2). We found 161 genes within these shared regions, but only 2 genes, *dysf* and *cyp26b1*, which contained adaptive loci within 20 kbp (Table 3). The *dysf* gene provides instructions for making a protein called dysferlin, which is found in the sarcolemma membrane that surrounds muscle fibers (Liu et al., 1998). This could indicate a role for muscle development in affecting skeletal development of the maxilla and premaxilla.

#### 3.3.3 Maxillary head protrusion: Parallel QTL for a correlated craniofacial trait

**FIGURE 6:**
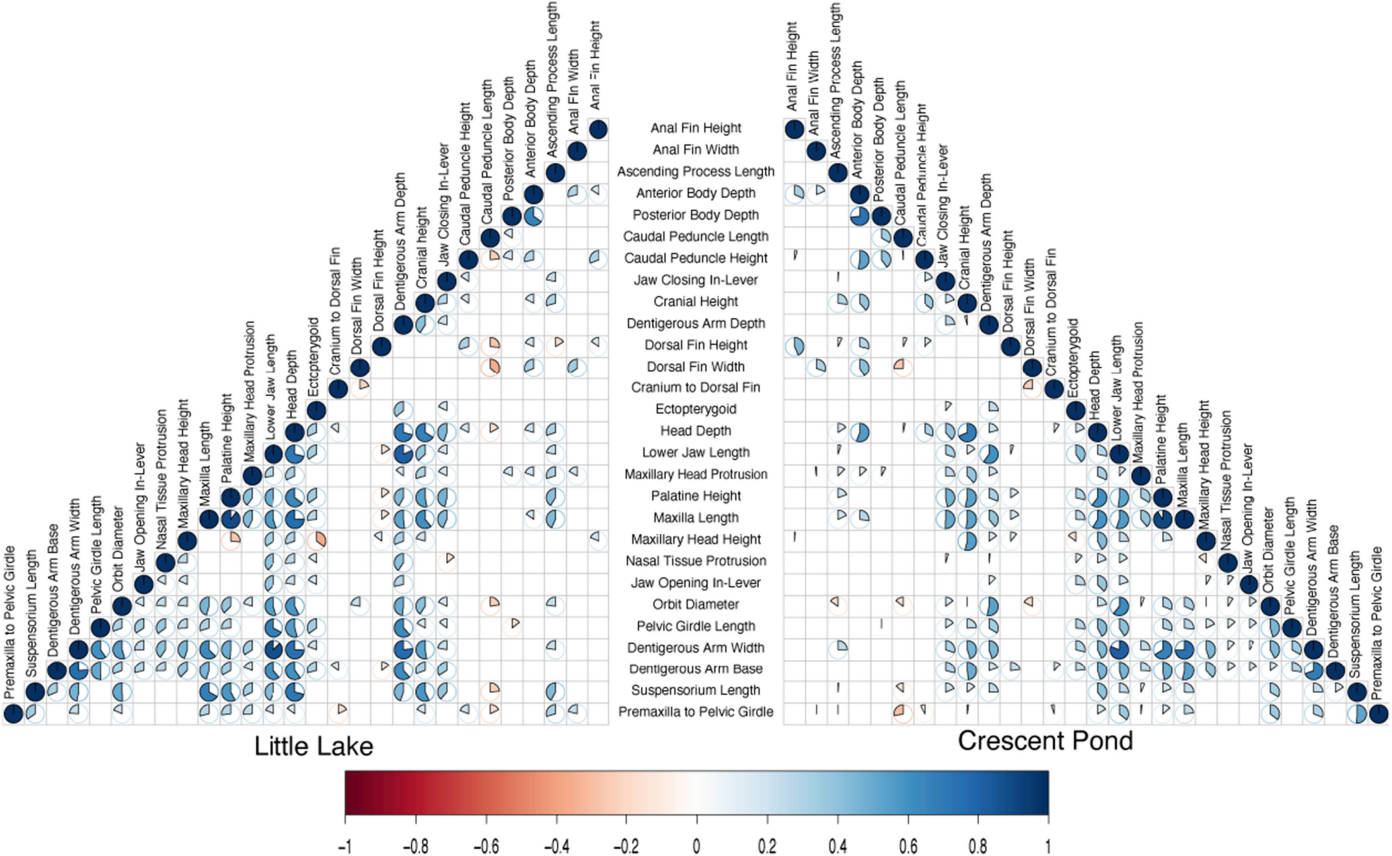
Correlation matrices depicting the relationship between phenotypic traits in Little Lake and Crescent Pond. Visualized pie charts represent relationships that are significant at the *p* < .05 level. Red pie charts represent negative relationships and blue pie charts represent positive relationships.

Maxillary head protrusion in Little Lake mapped to a QTL region on LG10 which corresponds to the max LOD scores for both lower jaw length and caudal peduncle height in Crescent Pond (Table 2; Figure 6). Across lakes, all three traits were associated with scaffolds 53, 2336, and 6275. We found 528 genes partially or fully within these shared regions, but only 21 of these genes contained adaptive alleles within 20 kbp (Table 3). One of these genes, *twist1*, contains a non‐synonymous substitution fixed in scale‐eating pupfish on San Salvador Island, Bahamas (Richards et al., 2021). *Twist1* is a transcription factor associated with palate development and oral jaw size in model organisms and tumor metastasis (Fan et al., 2021; Lee et al., 2014; Parsons et al., 2014; Teng et al., 2018).

#### 3.3.4 Jaw closing in‐lever: Parallel QTL for a correlated craniofacial trait

The QTL for jaw closing in‐lever was associated with LG 9 in Little Lake, which corresponds to the max LOD scores for orbit diameter and anterior body depth in Crescent Pond (Table 2; Figure 3). Scaffolds 8 and 8020 were associated with all three of these traits. We found 13 genes which partially or completely fell within these shared regions, and only two genes, *map2k6* and *galr2*, which contained adaptive alleles within 20 kbp (Table 3). *Galr2* was also previously detected within a significant QTL for lower jaw length in pupfish (Martin et al., 2017) and verified to play a novel craniofacial development function in this system through differential spatiotemporal patterns of expression in the oral jaws (Palominos et al., 2023).

#### 3.3.5 Dentigerous arm depth and adductor Mandibulae muscle mass: Parallel QTL for a correlated craniofacial trait

In Crescent Pond, the QTL for dentigerous arm depth and adductor mandibulae muscle mass mapped to the exact same location on LG 13 (95% CI dentigerous arm depth (0, 250), adductor mandibulae muscle mass (0,70)). This linkage group corresponds to LG14 in Little Lake, which contains the max LOD scores for both palatine height and suspensorium length (Table 3). We found 52 genes that overlapped between these regions, 18 of which contained adaptive loci. Furthermore, three of the genes—*ube2w, ncoa2, and prlh*—contained adaptive alleles that introgressed from Laguna Bavaro in the Dominican Republic to molluscivore pupfish (*ube2w*), from Lake Cunningham, New Providence Island to scale‐eating pupfish (*ncoa2*), or from North Carolina, USA to scale‐eating pupfish (*prlh*). We also found four genes that contained adaptive loci within 20 kbp that arose from de novo mutations: *cd226, cmbl, slc51a*, and *zfhx*; however, only one adaptive allele in *slc51a* is found within a coding region.

#### 3.3.6 Dentigerous arm depth: Non‐parallel QTL

Finally, the QTL for dentigerous arm depth in Little Lake was associated with LG 6, which corresponded to LG 7 in Crescent Pond; however, no traits from Crescent Pond mapped to this linkage group (Table 2; Figure 6). Instead, dentigerous arm depth in Crescent Pond was associated with LG 13 and did not share any similar genomic regions with those associated with dentigerous arm depth in Little Lake. We found 80 genes completely or partially within the 95% credible region for this QTL in Little Lake (Table S1), but none contained adaptive alleles based on our criteria. In fact, only a single adaptive allele was found in this QTL region, but it was in an unannotated region of the genome (Table 3).

### 3.4 Adaptive introgression within craniofacial QTL

Adaptive alleles originating from standing genetic variation across the Caribbean were most common within shared QTL regions between lakes (86.03% within scale‐eater populations, and 53.32% within molluscivore populations; Table 3). However, observed proportions within shared QTL were significantly less than expected by chance (scale‐eater expected 95% CI: (88.33%–90.37%), molluscivore expected 95% CI: (62%–67%;10,000 bootstrapped iterations)). Instead, we found more introgressed scale‐eater and molluscivore adaptive variants in shared QTL regions than expected by chance (Scale‐eater observed: 12.13% introgressed, scale‐eater expected 95% CI: (7.96%–9.88%); molluscivore observed: 46.67% introgressed, molluscivore expected 95% CI: (32.22%–37.06%)). Finally, we found that about 1.83% of adaptive alleles within overlapping regions between lakes originated from de novo mutations in scale‐eaters, however, this fell within the predicted null range (95% CI: (1.29%–2.17%)).

## 4 DISCUSSION

### 4.1 Parallel genetic changes underlie craniofacial divergence across populations

We found evidence supporting both parallel and non‐parallel genetic changes in an adaptive radiation of trophic specialist pupfishes. One QTL for cranial height was detected in both lakes mapping to the same genomic region with the same pattern of genetic dominance, supporting parallel evolution. Conversely, one QTL for premaxilla dentigerous arm depth was in both lakes, but it mapped to different locations, supporting non‐parallel evolution. We detected three additional QTL for craniofacial traits (dentigerous arm width, jaw closing in‐lever, maxillary head protrusion) in the Little Lake population that were not significant in Crescent Pond. However, all three genomic regions associated with these traits in Little Lake mapped to the maximum LOD score for the same set of craniofacial traits in Crescent Pond. Rather than assume independent QTL for each of these traits (non‐parallel evolution), we conclude that the same genomic regions are being reused in each lake and have pleiotropic effects on an integrated suite of craniofacial traits. Therefore, we found that 5 out of the 6 significant QTLs were reused in some way across lakes, suggesting that mostly parallel genetic changes underlie adaptive phenotypes in the San Salvador Island pupfish radiation, consistent with their history of adaptive introgression and gene flow among lakes (Martin et al., 2019; Martin & Feinstein, 2014; Richards et al., 2021).

### 4.2 High level of QTL reuse across populations

Overall, we found that 5 out of 6 significant QTL corresponded to parallel genetic changes—either affecting the same phenotypic trait or a tightly correlated craniofacial trait—across populations. The presence of both non‐parallel and parallel genetic changes leading to convergent phenotypes across lakes has been documented previously. For example, Colosimo et al. (2004) investigated the genetic basis of armor plate morphology in two independent threespine stickleback populations and found a single large effect locus on LG 4 in the two populations. However, they also noted a potential difference in the dominance relationships of alleles across ponds at this location and found additional differences in modifier QTLs between populations, suggesting that both parallel and non‐parallel genetic changes could lead to the loss of armor plating. Similarly, Erickson et al. (2016) found evidence for both parallel (43% of QTL regions overlapped between at least two populations) and non‐parallel (57% of QTL regions were found in only a single population) evolution for 36 skeletal phenotypes in three independent threespine stickleback populations. However, our findings suggest that pupfish exhibit a much higher proportion of parallel evolution than previously documented in stickleback. In fact, Conte et al. (2012) estimated that the probability of convergence via gene reuse is only 32%–55%—substantially lower than our results reported here— although this may be underestimated (Stern, 2013).

The increased proportion of parallel evolution estimated in this study results from our relaxed thresholds for detecting and categorizing shared QTL regions. Previous QTL studies have typically searched for evidence of parallel evolution by only looking for one‐to‐one mapping in which the same genomic regions are associated with the same trait across populations at a genome‐wide level of significance in each (Colosimo et al., 2004; Conte et al., 2012). While this method provides the most clear‐cut examples of parallel evolution, we argue that it vastly underestimates its frequency in nature. For example, this method would not consider reuse of the same genomic regions for integrated morphological traits as parallel evolution, a pattern observed in this study and in Erickson et al. (2016). Furthermore, the strict one‐to‐one trait QTL significance method for detecting parallel evolution does not include consideration of the hierarchy and diversity of convergence and parallel evolution, which can span morphological traits, ecotypes, performance, or even fitness (James et al., 2020; Rosenblum et al., 2014; Stern, 2013). Ultimately, we argue that our method of quantifying parallel evolution provides a more accurate view of the process and better captures the frequency of reuse of adaptive genetic variation in nature, given the ubiquity of pleiotropy (Cerca et al., 2023; Rowley et al., 2022; Sivakumaran et al., 2011; Solovieff et al., 2013) and gene flow and introgression among rapidly radiating species in nature (Gillespie et al., 2020; Martin & Richards, 2019; Poelstra et al., 2018; Richards et al., 2018, 2019).

### 4.3 Few QTL may affect many highly integrated craniofacial traits

There are several processes that may cause the same genomic regions to be associated with different traits between lakes. First, these genomic regions may be highly pleiotropic and affect several traits simultaneously. For example, Albert et al. (Albert et al., 2007) found that on average a single QTL affected 3.5 phenotypic traits in an analysis of 54 body traits in threespine stickleback. Wagner et al. (Wagner et al., 2008) found a similar pattern in QTL analyses of 70 skeletal traits in mice, where a single QTL affected on average 7.8 phenotypic traits (the maximum being 30). We also recently discovered a new function for *galr2* in craniofacial development using the pupfish system (Palominos et al., 2023), a gene which was previously only known to be involved in behavior and appetite (Lang et al., 2007; Mitsukawa et al., 2008), adding additional pleiotropic functions all relevant to adaptive axes for scale‐eating pupfish.

Alternatively, a single QTL region may contain many tightly linked causal variants that are responsible for variation in many traits. Correlated phenotypic traits are generally assumed to have a shared genetic basis, but it is often difficult to determine if this is due to pleiotropy or tight linkage between genomic regions (Gardner & Latta, 2007; Lynch & Walsh, 1998; Paaby & Rockman, 2013).

Finally, it may be that differences in methodology or sample sizes between lakes enable us to detect significant QTL for some traits in one lake and not the other. Our analyses of Little Lake allowed us to detect significant QTL for effect sizes greater than 6.54 PVE at 80% power, but we could only detect significant QTL for effect sizes greater than 8.41 PVE at 80% power in Crescent Pond due to our lower sample size for this cross (Sen et al., 2007). However, this level of power is typical in many non‐model QTL studies (Ashton et al., 2017) and was still quite similar between lakes in this study. Alternatively, the ability to detect a significant QTL in one lake but not the other may be further explained by our use of different sequencing methods between populations. However, we searched for regions within 10 kbp of one another between linkage maps to provide confidence that if we detected a significant QTL in one lake and not the other that it was not simply because that genomic region was not sequenced. For example, in Little Lake we detected a significant QTL associated with dentigerous arm depth on LG 6 but did not find any traits associated with this region in Crescent Pond.

### 4.4 Identifying candidate adaptive variants within QTL

Multiple mapping populations across lakes may also be particularly useful for identifying candidate causal variants. We found that one out of our six unique QTL regions mapped to the same genomic location across lakes and was associated with the same phenotypic trait—cranial height (Figure 3). In Crescent Pond, we found that a region of 110 cM was associated with this trait (LG10, position: 204, 95% CI (130–340)), which contained 426 genes. However, when we compared this region to the region independently identified in our Little Lake analysis, we found that the overlapping region was reduced to a 20 cM region (LG1, position: 259, 95% CI (250–270)) containing only 44 genes—a reduction of more than 80%. We found a similar pattern in the additional four QTL regions that mapped to the same genomic location across maps but were associated with different phenotypic traits and observed an average 56% reduction in region size. Erickson et al. (2016) used a similar method for identifying candidate QTL regions across three hybrid populations of stickleback and found that 43% of identified QTL regions were shared across two or more populations; however, they did not investigate whether these QTL regions completely or partially overlapped.

We also searched for adaptive SNPs within QTL regions that were identified in a previous study as (1) nearly fixed between species (*F*_st_ > 0.95) and (2) within a hard selective sweep (Richards et al., 2021). Overall, we found 789 genes within shared QTL regions across lakes; 45 of these genes were within 2 kb of adaptive variants (5.7%). For example, a variant in *twist1* was found within the region associated with maxillary head protrusion in Little Lake and lower jaw length and caudal peduncle height in Crescent Pond. *Twist1* is associated with palate and jaw development (Parsons et al., 2014; Teng et al., 2018), and previous genome‐wide association scans in pupfish showed that a region containing *twist1* was significantly associated with oral jaw size in the system (Richards et al., 2021). Similarly, we found that adaptive SNPs associated with *galr2* fell within a QTL region associated with jaw closing in‐lever (height of the articular coronoid process) in Little Lake and orbit diameter and anterior body depth in Crescent Pond; scaffolds 8 and 8020. Previous QTL mapping studies, gene expression studies, genome‐wide association analyses, and in situ hybridization and chemical inhibition experiments provide evidence that *galr2* functions in oral jaw development in pupfish (Martin et al., 2017; McGirr & Martin, 2017; Palominos et al., 2023; Richards et al., 2021).

### 4.5 Increased use of introgressed adaptive variants in QTL regions

We found more adaptive introgression from both scale‐eater (observed: 12.13% introgressed, expected 95% CI: (7.96%–9.88%)) and molluscivore populations within shared QTL regions than expected by chance (observed: 46.67%, expected 95% CI: (32.22%–37.06%)). This supports the prediction that introgressed variation should underlie parallel genetic changes (Stern, 2013; Thompson et al., 2019). Finally, we found that only 1.83% of adaptive alleles within shared QTL regions across both lakes originated from de novo mutations on San Salvador Island. While this percentage did not differ significantly from the expected estimates (expected 95% CI: 1.3%–2.17%), it does not eliminate the possibility that de novo mutations play an important adaptive role in pupfish evolution.

## 5 CONCLUSION

In conclusion, we found that a single QTL was responsible for variation in cranial height in both populations, and additional four shared QTLs were responsible for variation in different craniofacial traits across lakes, suggesting that parallel genetic changes underlie integrated suites of craniofacial traits on San Salvador Island. Adaptive alleles were more commonly found within shared QTL than expected by chance and were more likely to originate from introgression. We argue that fully measuring the extent of parallel evolution in nature (i.e., the reuse of shared genetic loci) requires a broader search for loci that may affect a suite of integrated traits and may not be above the significance threshold in all populations investigated.

## AUTHOR CONTRIBUTIONS

**Michelle E. St. John:** Formal analysis (equal); methodology (equal); visualization (equal); writing – original draft (equal). **Julia C. Dunker:** Investigation (equal); methodology (equal); writing – review and editing (equal). **Emilie J. Richards:** Formal analysis (equal); methodology (equal); writing – review and editing (equal). **Stephanie Romero:** Investigation (equal); methodology (equal). **Christopher H. Martin:** Conceptualization (lead); data curation (lead); funding acquisition (lead); investigation (lead); methodology (lead); project administration (lead); resources (lead); supervision (lead); validation (equal); writing – review and editing (lead).

## CONFLICT OF INTEREST STATEMENT

The authors declare no conflict of interest.

## Supporting information


Data S1.

## Data Availability

Genomes are archived at the National Center for Biotechnology Information BioProject Database (Accessions: PRJNA690558; PRJNA394148, PRJNA391309; and PRJNA305422). All phenotype and genotype data and all R scripts used for analyses are provided as supplemental material.
