# Supplementary material for: Parallel evolution of integrated craniofacial traits in trophic specialist pupfishes
Source: Ecol Evol. 2024 Jul 7;14(7):e11640. doi: 10.1002/ece3.11640 (PMC11228360; doi:10.1002/ece3.11640)
Supplement: Supplementary file 1 — Data S1. [file ECE3-14-e11640-s001.zip › ece311640-sup-0001-Supinfo.docx]

**Supplemental Figures and Tables:**

**Figure S1** Principal component analysis depicting phenotypic variation across 28 size corrected skeletal traits for Crescent Pond F2 hybrids (red circles) and Little Lake F2 hybrids (blue triangles). Skeletal traits were calculated as the mean from two lateral photographs from each individual. There was no significant difference between Crescent Pond and Little Lake individuals (MANOVA, df = 28, approximate F-value= 0.34, P = 1).

**
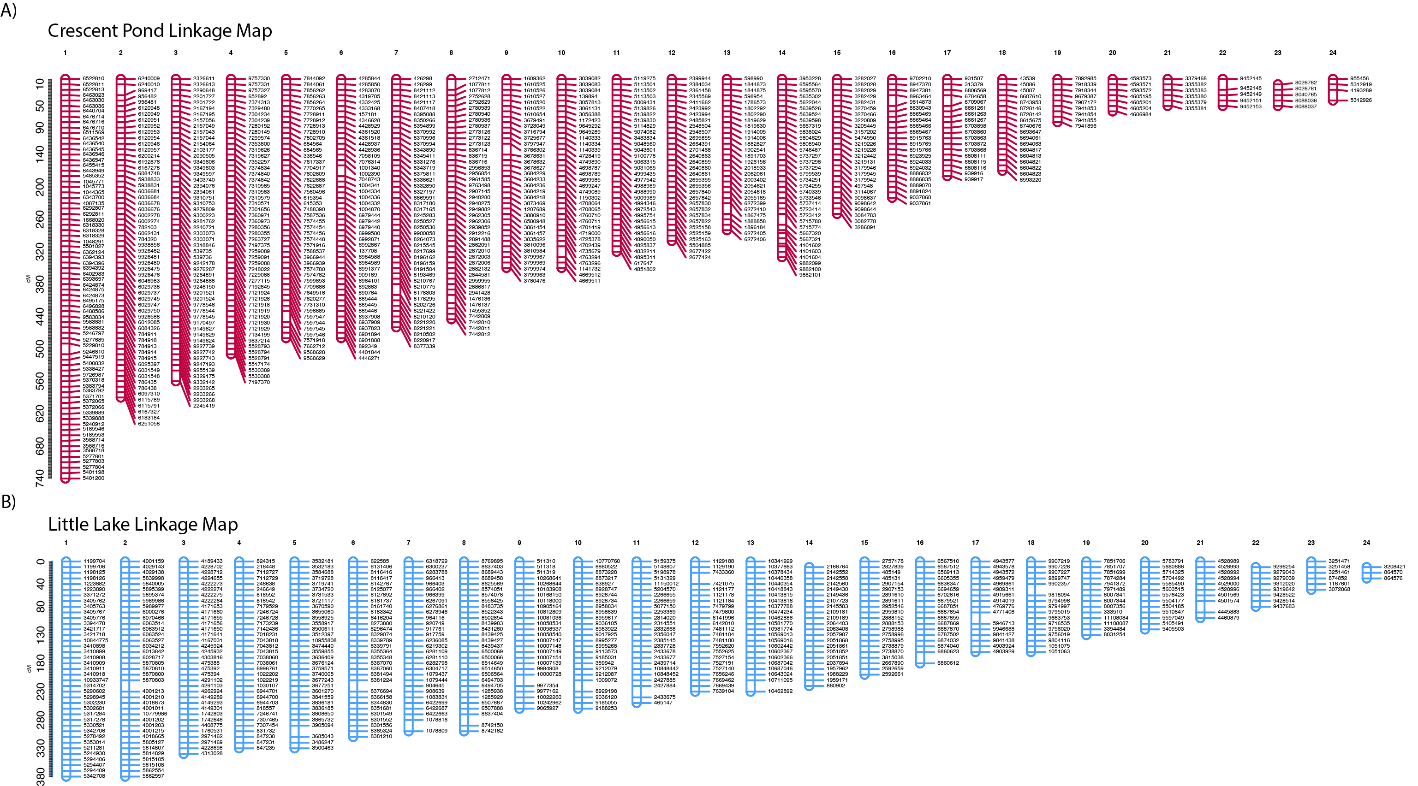
**

**Figure S2** Linkage maps for A) Crescent Pond and B) Little Lake crosses. The Crescent Pond linkage map was estimated from 743 markers and the Little Lake linkage map was estimated from 540 markers. Both maps were generated from crosses between a scale-eater (C. desquamator) and molluscivore (C. brontotheroides) from the respective lakes.

**Gene Table Supplement:**

**Supplemental Table 1** List of genes that fall within or partially within significant QTL regions.

| **Trait** | **Gene** | **Scaffold** | **Population** |
| --- | --- | --- | --- |
| Adductor Mandibulae Height | abca4 | HiC_scaffold_11 | CP |
| Adductor Mandibulae Height | agrp | HiC_scaffold_11 | CP |
| Adductor Mandibulae Height | ahrr | HiC_scaffold_11 | CP |
| Adductor Mandibulae Height | arhgap29 | HiC_scaffold_11 | CP |
| Adductor Mandibulae Height | b2m | HiC_scaffold_11 | CP |
| Adductor Mandibulae Height | bco1 | HiC_scaffold_11 | CP |
| Adductor Mandibulae Height | bloc1s5 | HiC_scaffold_11 | CP |
| Adductor Mandibulae Height | brpf3 | HiC_scaffold_11 | CP |
| Adductor Mandibulae Height | ccdc63 | HiC_scaffold_11 | CP |
| Adductor Mandibulae Height | cebpe | HiC_scaffold_11 | CP |
| Adductor Mandibulae Height | cngb3 | HiC_scaffold_11 | CP |
| Adductor Mandibulae Height | col11a1 | HiC_scaffold_11 | CP |
| Adductor Mandibulae Height | cpne3 | HiC_scaffold_11 | CP |
| Adductor Mandibulae Height | dcaf11 | HiC_scaffold_11 | CP |
| Adductor Mandibulae Height | dph2 | HiC_scaffold_11 | CP |
| Adductor Mandibulae Height | eef1e1 | HiC_scaffold_11 | CP |
| Adductor Mandibulae Height | emp3 | HiC_scaffold_11 | CP |
| Adductor Mandibulae Height | fam168b | HiC_scaffold_11 | CP |
| Adductor Mandibulae Height | fam83e | HiC_scaffold_11 | CP |
| Adductor Mandibulae Height | fen1 | HiC_scaffold_11 | CP |
| Adductor Mandibulae Height | fitm1 | HiC_scaffold_11 | CP |
| Adductor Mandibulae Height | foxj3 | HiC_scaffold_11 | CP |
| Adductor Mandibulae Height | garem1 | HiC_scaffold_11 | CP |
| Adductor Mandibulae Height | glyctk | HiC_scaffold_11 | CP |
| Adductor Mandibulae Height | hbp1 | HiC_scaffold_11 | CP |
| Adductor Mandibulae Height | hmcn2 | HiC_scaffold_11 | CP |
| Adductor Mandibulae Height | kazn | HiC_scaffold_11 | CP |
| Adductor Mandibulae Height | kazna | HiC_scaffold_11 | CP |
| Adductor Mandibulae Height | kbp | HiC_scaffold_11 | CP |
| Adductor Mandibulae Height | kif20a | HiC_scaffold_11 | CP |
| Adductor Mandibulae Height | mag | HiC_scaffold_11 | CP |
| Adductor Mandibulae Height | mak | HiC_scaffold_11 | CP |
| Adductor Mandibulae Height | mcur1 | HiC_scaffold_11 | CP |
| Adductor Mandibulae Height | mon1b | HiC_scaffold_11 | CP |
| Adductor Mandibulae Height | myh6 | HiC_scaffold_11 | CP |
| Adductor Mandibulae Height | myh7 | HiC_scaffold_11 | CP |
| Adductor Mandibulae Height | ngdn | HiC_scaffold_11 | CP |
| Adductor Mandibulae Height | nrros | HiC_scaffold_11 | CP |
| Adductor Mandibulae Height | ntng1 | HiC_scaffold_11 | CP |
| Adductor Mandibulae Height | olfm3 | HiC_scaffold_11 | CP |
| Adductor Mandibulae Height | pabpn1 | HiC_scaffold_11 | CP |
| Adductor Mandibulae Height | pck2 | HiC_scaffold_11 | CP |
| Adductor Mandibulae Height | pdcd6 | HiC_scaffold_11 | CP |
| Adductor Mandibulae Height | phldb2 | HiC_scaffold_11 | CP |
| Adductor Mandibulae Height | pigm | HiC_scaffold_11 | CP |
| Adductor Mandibulae Height | pkia | HiC_scaffold_11 | CP |
| Adductor Mandibulae Height | plch2 | HiC_scaffold_11 | CP |
| Adductor Mandibulae Height | plcxd2 | HiC_scaffold_11 | CP |
| Adductor Mandibulae Height | pomp | HiC_scaffold_11 | CP |
| Adductor Mandibulae Height | ppcs | HiC_scaffold_11 | CP |
| Adductor Mandibulae Height | prdm2 | HiC_scaffold_11 | CP |
| Adductor Mandibulae Height | prkdc | HiC_scaffold_11 | CP |
| Adductor Mandibulae Height | prmt6 | HiC_scaffold_11 | CP |
| Adductor Mandibulae Height | psme1 | HiC_scaffold_11 | CP |
| Adductor Mandibulae Height | ramp3 | HiC_scaffold_11 | CP |
| Adductor Mandibulae Height | rec8 | HiC_scaffold_11 | CP |
| Adductor Mandibulae Height | rgs9bp-b | HiC_scaffold_11 | CP |
| Adductor Mandibulae Height | rmdn1 | HiC_scaffold_11 | CP |
| Adductor Mandibulae Height | rnpc3 | HiC_scaffold_11 | CP |
| Adductor Mandibulae Height | rps20 | HiC_scaffold_11 | CP |
| Adductor Mandibulae Height | serpinb1 | HiC_scaffold_11 | CP |
| Adductor Mandibulae Height | serpinb10 | HiC_scaffold_11 | CP |
| Adductor Mandibulae Height | serpinb1b | HiC_scaffold_11 | CP |
| Adductor Mandibulae Height | serpinb6 | HiC_scaffold_11 | CP |
| Adductor Mandibulae Height | sh3glb1 | HiC_scaffold_11 | CP |
| Adductor Mandibulae Height | siglec1 | HiC_scaffold_11 | CP |
| Adductor Mandibulae Height | siglec10 | HiC_scaffold_11 | CP |
| Adductor Mandibulae Height | siglec13 | HiC_scaffold_11 | CP |
| Adductor Mandibulae Height | siglec14 | HiC_scaffold_11 | CP |
| Adductor Mandibulae Height | siglec9 | HiC_scaffold_11 | CP |
| Adductor Mandibulae Height | ski | HiC_scaffold_11 | CP |
| Adductor Mandibulae Height | slc22a17 | HiC_scaffold_11 | CP |
| Adductor Mandibulae Height | slc35b3 | HiC_scaffold_11 | CP |
| Adductor Mandibulae Height | snx16 | HiC_scaffold_11 | CP |
| Adductor Mandibulae Height | tecr | HiC_scaffold_11 | CP |
| Adductor Mandibulae Height | tfap2a | HiC_scaffold_11 | CP |
| Adductor Mandibulae Height | thap6 | HiC_scaffold_11 | CP |
| Adductor Mandibulae Height | thtpa | HiC_scaffold_11 | CP |
| Adductor Mandibulae Height | tm9sf1 | HiC_scaffold_11 | CP |
| Adductor Mandibulae Height | tmem14c | HiC_scaffold_11 | CP |
| Adductor Mandibulae Height | tmem51 | HiC_scaffold_11 | CP |
| Adductor Mandibulae Height | tmem56-b | HiC_scaffold_11 | CP |
| Adductor Mandibulae Height | txndc5 | HiC_scaffold_11 | CP |
| Adductor Mandibulae Height | utp3 | HiC_scaffold_11 | CP |
| Adductor Mandibulae Height | vav3 | HiC_scaffold_11 | CP |
| Adductor Mandibulae Height | wdr19 | HiC_scaffold_11 | CP |
| Adductor Mandibulae Height | wdr37 | HiC_scaffold_11 | CP |
| Adductor Mandibulae Height | wwp1 | HiC_scaffold_11 | CP |
| Adductor Mandibulae Height | zc2hc1a | HiC_scaffold_11 | CP |
| Adductor Mandibulae Height | zfhx4 | HiC_scaffold_11 | CP |
| Cranial Height | aak1 | HiC_scaffold_33 | CP |
| Cranial Height | acacb | HiC_scaffold_33 | CP |
| Cranial Height | acdh-11 | HiC_scaffold_33 | CP |
| Cranial Height | adra1a | HiC_scaffold_33 | CP |
| Cranial Height | adrb4c | HiC_scaffold_33 | CP |
| Cranial Height | aebp1 | HiC_scaffold_33 | CP |
| Cranial Height | aifm3 | HiC_scaffold_33 | CP |
| Cranial Height | akr1a1a | HiC_scaffold_33 | CP |
| Cranial Height | aldh3b1 | HiC_scaffold_33 | CP |
| Cranial Height | amy2 | HiC_scaffold_33 | CP |
| Cranial Height | ank1 | HiC_scaffold_33 | CP |
| Cranial Height | ank1 | HiC_scaffold_33 | LL |
| Cranial Height | ankrd13a | HiC_scaffold_33 | CP |
| Cranial Height | ankrd39 | HiC_scaffold_33 | CP |
| Cranial Height | antxr1 | HiC_scaffold_33 | CP |
| Cranial Height | aopep | HiC_scaffold_39 | CP |
| Cranial Height | ap1b1 | HiC_scaffold_33 | CP |
| Cranial Height | ap1b1 | HiC_scaffold_33 | LL |
| Cranial Height | aqp3 | HiC_scaffold_33 | CP |
| Cranial Height | arl6ip4 | HiC_scaffold_33 | CP |
| Cranial Height | arl6ip4 | HiC_scaffold_33 | LL |
| Cranial Height | arrdc3 | HiC_scaffold_33 | CP |
| Cranial Height | arsk | HiC_scaffold_33 | CP |
| Cranial Height | asb6 | HiC_scaffold_33 | CP |
| Cranial Height | atp6v0a2 | HiC_scaffold_33 | CP |
| Cranial Height | atp6v0a2 | HiC_scaffold_33 | LL |
| Cranial Height | bag4 | HiC_scaffold_33 | CP |
| Cranial Height | bco2 | HiC_scaffold_33 | CP |
| Cranial Height | bin3 | HiC_scaffold_33 | CP |
| Cranial Height | bmp1 | HiC_scaffold_33 | CP |
| Cranial Height | bri3bp | HiC_scaffold_33 | CP |
| Cranial Height | bri3bp | HiC_scaffold_33 | LL |
| Cranial Height | c2cd4cc2cd4_family | HiC_scaffold_33 | CP |
| Cranial Height | c2cd4cc2cd4_family | HiC_scaffold_33 | LL |
| Cranial Height | c9orf78 | HiC_scaffold_33 | CP |
| Cranial Height | carnmt1 | HiC_scaffold_33 | CP |
| Cranial Height | ccdc117 | HiC_scaffold_33 | CP |
| Cranial Height | ccdc117 | HiC_scaffold_33 | LL |
| Cranial Height | ccdc157 | HiC_scaffold_33 | CP |
| Cranial Height | ccdc92 | HiC_scaffold_33 | CP |
| Cranial Height | ccnh | HiC_scaffold_33 | CP |
| Cranial Height | cemip2 | HiC_scaffold_33 | CP |
| Cranial Height | ciao1a | HiC_scaffold_33 | CP |
| Cranial Height | cldn22 | HiC_scaffold_33 | CP |
| Cranial Height | clip1 | HiC_scaffold_33 | CP |
| Cranial Height | cmlkr1 | HiC_scaffold_33 | CP |
| Cranial Height | coe2 | HiC_scaffold_33 | CP |
| Cranial Height | coe2 | HiC_scaffold_33 | LL |
| Cranial Height | cox7c | HiC_scaffold_33 | CP |
| Cranial Height | crat | HiC_scaffold_33 | CP |
| Cranial Height | ctrc | HiC_scaffold_33 | CP |
| Cranial Height | dao | HiC_scaffold_33 | CP |
| Cranial Height | dbn1 | HiC_scaffold_33 | CP |
| Cranial Height | dbnl | HiC_scaffold_33 | CP |
| Cranial Height | ddr2 | HiC_scaffold_33 | CP |
| Cranial Height | dguok | HiC_scaffold_33 | CP |
| Cranial Height | dguok | HiC_scaffold_33 | LL |
| Cranial Height | disp3 | HiC_scaffold_33 | CP |
| Cranial Height | dnajb5 | HiC_scaffold_33 | CP |
| Cranial Height | dnm1l | HiC_scaffold_33 | CP |
| Cranial Height | dpysl2 | HiC_scaffold_33 | CP |
| Cranial Height | dusp18 | HiC_scaffold_33 | CP |
| Cranial Height | dusp26 | HiC_scaffold_33 | CP |
| Cranial Height | ehd1 | HiC_scaffold_33 | CP |
| Cranial Height | eif4ebp1 | HiC_scaffold_33 | CP |
| Cranial Height | eif4ebp1 | HiC_scaffold_33 | LL |
| Cranial Height | elac1 | HiC_scaffold_39 | CP |
| Cranial Height | elmod3 | HiC_scaffold_33 | CP |
| Cranial Height | elovl7 | HiC_scaffold_39 | CP |
| Cranial Height | emid1 | HiC_scaffold_33 | CP |
| Cranial Height | emid1 | HiC_scaffold_33 | LL |
| Cranial Height | epx | HiC_scaffold_33 | CP |
| Cranial Height | epx | HiC_scaffold_33 | LL |
| Cranial Height | erap2 | HiC_scaffold_33 | CP |
| Cranial Height | ercc8 | HiC_scaffold_39 | CP |
| Cranial Height | es1 | HiC_scaffold_33 | CP |
| Cranial Height | ewsr1 | HiC_scaffold_33 | CP |
| Cranial Height | ewsr1 | HiC_scaffold_33 | LL |
| Cranial Height | f2r | HiC_scaffold_33 | CP |
| Cranial Height | fabp1 | HiC_scaffold_39 | CP |
| Cranial Height | fam173b | HiC_scaffold_39 | CP |
| Cranial Height | fam219a | HiC_scaffold_33 | CP |
| Cranial Height | fam222a | HiC_scaffold_33 | CP |
| Cranial Height | fancg | HiC_scaffold_33 | CP |
| Cranial Height | fgfr1a | HiC_scaffold_33 | CP |
| Cranial Height | ficd | HiC_scaffold_33 | CP |
| Cranial Height | foxb2 | HiC_scaffold_33 | CP |
| Cranial Height | foxb2 | HiC_scaffold_33 | LL |
| Cranial Height | foxd5-a | HiC_scaffold_39 | CP |
| Cranial Height | foxn4 | HiC_scaffold_33 | CP |
| Cranial Height | fzd10-a | HiC_scaffold_33 | CP |
| Cranial Height | gal3st1 | HiC_scaffold_33 | CP |
| Cranial Height | gas2l1 | HiC_scaffold_33 | CP |
| Cranial Height | gas2l1 | HiC_scaffold_33 | LL |
| Cranial Height | gatc | HiC_scaffold_33 | CP |
| Cranial Height | gcnt1 | HiC_scaffold_33 | CP |
| Cranial Height | gfra2 | HiC_scaffold_33 | CP |
| Cranial Height | gimap2 | HiC_scaffold_33 | CP |
| Cranial Height | gimap4 | HiC_scaffold_33 | CP |
| Cranial Height | gimap7 | HiC_scaffold_33 | CP |
| Cranial Height | gimap8 | HiC_scaffold_33 | CP |
| Cranial Height | gins4 | HiC_scaffold_33 | CP |
| Cranial Height | git2 | HiC_scaffold_33 | CP |
| Cranial Height | gltp | HiC_scaffold_33 | CP |
| Cranial Height | gna14 | HiC_scaffold_33 | CP |
| Cranial Height | gna14 | HiC_scaffold_33 | LL |
| Cranial Height | gnaq | HiC_scaffold_33 | CP |
| Cranial Height | gnaq | HiC_scaffold_33 | LL |
| Cranial Height | gnrh1 | HiC_scaffold_33 | CP |
| Cranial Height | gpat4 | HiC_scaffold_33 | CP |
| Cranial Height | gramd2b | HiC_scaffold_33 | CP |
| Cranial Height | grk5 | HiC_scaffold_33 | CP |
| Cranial Height | gtf2h3 | HiC_scaffold_33 | CP |
| Cranial Height | hapln1 | HiC_scaffold_33 | CP |
| Cranial Height | hcar2 | HiC_scaffold_33 | CP |
| Cranial Height | hcar2 | HiC_scaffold_33 | LL |
| Cranial Height | hip1r | HiC_scaffold_33 | CP |
| Cranial Height | hip1r | HiC_scaffold_33 | LL |
| Cranial Height | homer1 | HiC_scaffold_33 | CP |
| Cranial Height | hspb11 | HiC_scaffold_33 | CP |
| Cranial Height | ier5l | HiC_scaffold_33 | CP |
| Cranial Height | igsf9 | HiC_scaffold_33 | CP |
| Cranial Height | ine | HiC_scaffold_33 | CP |
| Cranial Height | ine | HiC_scaffold_33 | LL |
| Cranial Height | iqgap1 | HiC_scaffold_33 | CP |
| Cranial Height | iscu | HiC_scaffold_33 | CP |
| Cranial Height | jmy | HiC_scaffold_33 | CP |
| Cranial Height | kansl3 | HiC_scaffold_33 | CP |
| Cranial Height | kctd10 | HiC_scaffold_33 | CP |
| Cranial Height | kctd9 | HiC_scaffold_33 | CP |
| Cranial Height | kiaa0825 | HiC_scaffold_33 | CP |
| Cranial Height | kif24 | HiC_scaffold_33 | CP |
| Cranial Height | kin14e | HiC_scaffold_33 | CP |
| Cranial Height | klf9 | HiC_scaffold_33 | CP |
| Cranial Height | klhl10 | HiC_scaffold_33 | CP |
| Cranial Height | klhl10 | HiC_scaffold_33 | LL |
| Cranial Height | kmt5aa | HiC_scaffold_33 | CP |
| Cranial Height | kmt5aa | HiC_scaffold_33 | LL |
| Cranial Height | kntc1 | HiC_scaffold_33 | CP |
| Cranial Height | koza | HiC_scaffold_33 | CP |
| Cranial Height | lgi3 | HiC_scaffold_33 | CP |
| Cranial Height | limk2 | HiC_scaffold_33 | CP |
| Cranial Height | limk2 | HiC_scaffold_33 | LL |
| Cranial Height | lix1 | HiC_scaffold_33 | CP |
| Cranial Height | lox | HiC_scaffold_33 | CP |
| Cranial Height | loxhd1 | HiC_scaffold_39 | CP |
| Cranial Height | lsm1 | HiC_scaffold_33 | CP |
| Cranial Height | lysmd3 | HiC_scaffold_33 | CP |
| Cranial Height | lztr1 | HiC_scaffold_33 | CP |
| Cranial Height | lzts1 | HiC_scaffold_33 | CP |
| Cranial Height | mapk4 | HiC_scaffold_39 | CP |
| Cranial Height | mblac2 | HiC_scaffold_33 | CP |
| Cranial Height | mctp1 | HiC_scaffold_33 | CP |
| Cranial Height | me2 | HiC_scaffold_39 | CP |
| Cranial Height | med22 | HiC_scaffold_33 | CP |
| Cranial Height | mfsd3 | HiC_scaffold_33 | CP |
| Cranial Height | mier3 | HiC_scaffold_39 | CP |
| Cranial Height | mlxip | HiC_scaffold_33 | CP |
| Cranial Height | mmab | HiC_scaffold_33 | CP |
| Cranial Height | mn1 | HiC_scaffold_33 | CP |
| Cranial Height | mob1a | HiC_scaffold_33 | CP |
| Cranial Height | mob1a | HiC_scaffold_33 | LL |
| Cranial Height | morc2a | HiC_scaffold_33 | CP |
| Cranial Height | mspa | HiC_scaffold_33 | CP |
| Cranial Height | mthfd2 | HiC_scaffold_33 | CP |
| Cranial Height | mthfd2 | HiC_scaffold_33 | LL |
| Cranial Height | mtx3 | HiC_scaffold_33 | CP |
| Cranial Height | mvk | HiC_scaffold_33 | CP |
| Cranial Height | myo1h | HiC_scaffold_33 | CP |
| Cranial Height | ncs1 | HiC_scaffold_33 | CP |
| Cranial Height | ndufaf2 | HiC_scaffold_39 | CP |
| Cranial Height | nefh | HiC_scaffold_33 | CP |
| Cranial Height | nefl | HiC_scaffold_33 | CP |
| Cranial Height | neurl3 | HiC_scaffold_33 | CP |
| Cranial Height | nfu1 | HiC_scaffold_33 | CP |
| Cranial Height | nkx2-6 | HiC_scaffold_33 | CP |
| Cranial Height | nkx6-3 | HiC_scaffold_33 | CP |
| Cranial Height | nodal | HiC_scaffold_33 | CP |
| Cranial Height | nodal | HiC_scaffold_33 | LL |
| Cranial Height | nol6 | HiC_scaffold_33 | CP |
| Cranial Height | nono | HiC_scaffold_33 | CP |
| Cranial Height | npc1l1 | HiC_scaffold_33 | CP |
| Cranial Height | npy1r | HiC_scaffold_33 | CP |
| Cranial Height | omg | HiC_scaffold_33 | CP |
| Cranial Height | osbp2 | HiC_scaffold_33 | CP |
| Cranial Height | osbp2 | HiC_scaffold_33 | LL |
| Cranial Height | p2rx2 | HiC_scaffold_33 | CP |
| Cranial Height | p2ry14 | HiC_scaffold_33 | CP |
| Cranial Height | p2ry14 | HiC_scaffold_33 | LL |
| Cranial Height | pcsk5 | HiC_scaffold_33 | CP |
| Cranial Height | pde4d | HiC_scaffold_39 | CP |
| Cranial Height | pdlim2 | HiC_scaffold_33 | CP |
| Cranial Height | pebp4 | HiC_scaffold_33 | CP |
| Cranial Height | pes1 | HiC_scaffold_33 | CP |
| Cranial Height | pgbd3 | HiC_scaffold_33 | CP |
| Cranial Height | pgbd3 | HiC_scaffold_33 | LL |
| Cranial Height | pgm5 | HiC_scaffold_39 | CP |
| Cranial Height | phyhip | HiC_scaffold_33 | CP |
| Cranial Height | pik3ip1 | HiC_scaffold_33 | CP |
| Cranial Height | pik3ip1 | HiC_scaffold_33 | LL |
| Cranial Height | pip5k1b | HiC_scaffold_33 | CP |
| Cranial Height | pitpnb | HiC_scaffold_33 | CP |
| Cranial Height | pitpnm2 | HiC_scaffold_33 | CP |
| Cranial Height | pitpnm2 | HiC_scaffold_33 | LL |
| Cranial Height | pla2g3 | HiC_scaffold_33 | CP |
| Cranial Height | plcl1 | HiC_scaffold_33 | CP |
| Cranial Height | plekha2 | HiC_scaffold_33 | CP |
| Cranial Height | plk2 | HiC_scaffold_39 | CP |
| Cranial Height | pole | HiC_scaffold_33 | CP |
| Cranial Height | polr3d | HiC_scaffold_33 | CP |
| Cranial Height | polr3g | HiC_scaffold_33 | CP |
| Cranial Height | ppp1r3c | HiC_scaffold_33 | CP |
| Cranial Height | prlhr | HiC_scaffold_33 | CP |
| Cranial Height | prune2 | HiC_scaffold_33 | CP |
| Cranial Height | prune2 | HiC_scaffold_33 | LL |
| Cranial Height | psap | HiC_scaffold_33 | CP |
| Cranial Height | psap | HiC_scaffold_33 | LL |
| Cranial Height | psbp1 | HiC_scaffold_33 | CP |
| Cranial Height | psmd9 | HiC_scaffold_33 | CP |
| Cranial Height | ptch1 | HiC_scaffold_39 | CP |
| Cranial Height | ptger4 | HiC_scaffold_39 | CP |
| Cranial Height | pxmp2 | HiC_scaffold_33 | CP |
| Cranial Height | rab11fip1 | HiC_scaffold_33 | CP |
| Cranial Height | rab3c | HiC_scaffold_39 | CP |
| Cranial Height | rabgef1 | HiC_scaffold_33 | CP |
| Cranial Height | rasa1 | HiC_scaffold_33 | CP |
| Cranial Height | rasl10b | HiC_scaffold_33 | CP |
| Cranial Height | rasl10b | HiC_scaffold_33 | LL |
| Cranial Height | rfesd | HiC_scaffold_33 | CP |
| Cranial Height | rflna | HiC_scaffold_33 | CP |
| Cranial Height | rgmb | HiC_scaffold_33 | CP |
| Cranial Height | rhbdd3 | HiC_scaffold_33 | CP |
| Cranial Height | rhbdd3 | HiC_scaffold_33 | LL |
| Cranial Height | rilpl1 | HiC_scaffold_33 | CP |
| Cranial Height | rilpl1 | HiC_scaffold_33 | LL |
| Cranial Height | rilpl2 | HiC_scaffold_33 | CP |
| Cranial Height | rilpl2 | HiC_scaffold_33 | LL |
| Cranial Height | rimbp2 | HiC_scaffold_33 | CP |
| Cranial Height | riok2 | HiC_scaffold_33 | CP |
| Cranial Height | rnf214 | HiC_scaffold_33 | CP |
| Cranial Height | rnf223 | HiC_scaffold_33 | CP |
| Cranial Height | rorb | HiC_scaffold_33 | CP |
| Cranial Height | rph3a | HiC_scaffold_33 | CP |
| Cranial Height | rsrc2 | HiC_scaffold_33 | CP |
| Cranial Height | rtkn | HiC_scaffold_33 | CP |
| Cranial Height | sart3 | HiC_scaffold_33 | CP |
| Cranial Height | sds | HiC_scaffold_33 | CP |
| Cranial Height | seca | HiC_scaffold_33 | CP |
| Cranial Height | setbp1 | HiC_scaffold_33 | CP |
| Cranial Height | sgsm1 | HiC_scaffold_33 | CP |
| Cranial Height | slc15a4 | HiC_scaffold_33 | CP |
| Cranial Height | slc25a37 | HiC_scaffold_33 | CP |
| Cranial Height | slc2a11 | HiC_scaffold_33 | CP |
| Cranial Height | slc2a8 | HiC_scaffold_33 | CP |
| Cranial Height | slc6a4 | HiC_scaffold_33 | CP |
| Cranial Height | slc7a4 | HiC_scaffold_33 | CP |
| Cranial Height | slc8b1 | HiC_scaffold_33 | CP |
| Cranial Height | slf1 | HiC_scaffold_33 | CP |
| Cranial Height | smim15 | HiC_scaffold_39 | CP |
| Cranial Height | smn1 | HiC_scaffold_33 | CP |
| Cranial Height | smtn | HiC_scaffold_33 | CP |
| Cranial Height | smtn | HiC_scaffold_33 | LL |
| Cranial Height | smtnl1 | HiC_scaffold_33 | CP |
| Cranial Height | smtnl1 | HiC_scaffold_33 | LL |
| Cranial Height | smyd1 | HiC_scaffold_39 | CP |
| Cranial Height | snrnp200 | HiC_scaffold_33 | CP |
| Cranial Height | snx2 | HiC_scaffold_33 | CP |
| Cranial Height | sorbs3 | HiC_scaffold_33 | CP |
| Cranial Height | srrd | HiC_scaffold_33 | CP |
| Cranial Height | ssbp2 | HiC_scaffold_33 | CP |
| Cranial Height | ssh1 | HiC_scaffold_33 | CP |
| Cranial Height | star | HiC_scaffold_33 | CP |
| Cranial Height | stx2 | HiC_scaffold_33 | CP |
| Cranial Height | sv2c | HiC_scaffold_33 | CP |
| Cranial Height | svop | HiC_scaffold_33 | CP |
| Cranial Height | tacc1 | HiC_scaffold_33 | CP |
| Cranial Height | tbx5 | HiC_scaffold_33 | CP |
| Cranial Height | tcf7l1a | HiC_scaffold_33 | CP |
| Cranial Height | tchp | HiC_scaffold_33 | CP |
| Cranial Height | tcn2 | HiC_scaffold_33 | CP |
| Cranial Height | tcn2 | HiC_scaffold_33 | LL |
| Cranial Height | tctn2 | HiC_scaffold_33 | CP |
| Cranial Height | tctn2 | HiC_scaffold_33 | LL |
| Cranial Height | tent2 | HiC_scaffold_33 | CP |
| Cranial Height | tfip11 | HiC_scaffold_33 | CP |
| Cranial Height | thbs4b | HiC_scaffold_33 | CP |
| Cranial Height | thoc5 | HiC_scaffold_33 | CP |
| Cranial Height | tmem119 | HiC_scaffold_33 | CP |
| Cranial Height | tmem127 | HiC_scaffold_33 | CP |
| Cranial Height | tmem132c | HiC_scaffold_33 | CP |
| Cranial Height | tmem132d | HiC_scaffold_33 | CP |
| Cranial Height | tmem161b | HiC_scaffold_33 | CP |
| Cranial Height | tmem167a | HiC_scaffold_33 | CP |
| Cranial Height | tmem230 | HiC_scaffold_33 | CP |
| Cranial Height | tmem248 | HiC_scaffold_33 | CP |
| Cranial Height | tnks | HiC_scaffold_33 | CP |
| Cranial Height | tpcn1 | HiC_scaffold_33 | CP |
| Cranial Height | trafd1 | HiC_scaffold_33 | CP |
| Cranial Height | trafd1 | HiC_scaffold_33 | LL |
| Cranial Height | trpm3 | HiC_scaffold_33 | CP |
| Cranial Height | trpm6 | HiC_scaffold_33 | CP |
| Cranial Height | tspan36 | HiC_scaffold_39 | CP |
| Cranial Height | ttc28 | HiC_scaffold_33 | CP |
| Cranial Height | ttc37 | HiC_scaffold_33 | CP |
| Cranial Height | tutl | HiC_scaffold_33 | CP |
| Cranial Height | ube3b | HiC_scaffold_33 | CP |
| Cranial Height | ubl4aa | HiC_scaffold_33 | CP |
| Cranial Height | ulk1 | HiC_scaffold_33 | CP |
| Cranial Height | unc45b | HiC_scaffold_33 | CP |
| Cranial Height | usp30 | HiC_scaffold_33 | CP |
| Cranial Height | usp39 | HiC_scaffold_33 | CP |
| Cranial Height | usp39 | HiC_scaffold_33 | LL |
| Cranial Height | vcp | HiC_scaffold_33 | CP |
| Cranial Height | vegt | HiC_scaffold_33 | CP |
| Cranial Height | vps13c | HiC_scaffold_33 | CP |
| Cranial Height | vps33a | HiC_scaffold_33 | CP |
| Cranial Height | wdr31 | HiC_scaffold_33 | CP |
| Cranial Height | wdr31 | HiC_scaffold_33 | LL |
| Cranial Height | wdr66 | HiC_scaffold_33 | CP |
| Cranial Height | wscd2 | HiC_scaffold_33 | CP |
| Cranial Height | xrcc4 | HiC_scaffold_33 | CP |
| Cranial Height | zfand5 | HiC_scaffold_33 | CP |
| Cranial Height | znf180 | HiC_scaffold_33 | CP |
| Cranial Height | znf366 | HiC_scaffold_39 | CP |
| Cranial Height | znf608 | HiC_scaffold_33 | CP |
| Dentigerous Arm Depth | abca4 | HiC_scaffold_11 | CP |
| Dentigerous Arm Depth | abhd10 | HiC_scaffold_11 | CP |
| Dentigerous Arm Depth | abi1 | HiC_scaffold_11 | CP |
| Dentigerous Arm Depth | acad11 | HiC_scaffold_11 | CP |
| Dentigerous Arm Depth | acbd5a | HiC_scaffold_11 | CP |
| Dentigerous Arm Depth | ackr4 | HiC_scaffold_11 | CP |
| Dentigerous Arm Depth | adamts12 | HiC_scaffold_5 | LL |
| Dentigerous Arm Depth | adamts7 | HiC_scaffold_5 | LL |
| Dentigerous Arm Depth | adgrg4 | HiC_scaffold_11 | CP |
| Dentigerous Arm Depth | agrp | HiC_scaffold_11 | CP |
| Dentigerous Arm Depth | agtr1 | HiC_scaffold_11 | CP |
| Dentigerous Arm Depth | ahrr | HiC_scaffold_11 | CP |
| Dentigerous Arm Depth | akap13 | HiC_scaffold_5 | LL |
| Dentigerous Arm Depth | amer2 | HiC_scaffold_11 | CP |
| Dentigerous Arm Depth | ankh | HiC_scaffold_11 | CP |
| Dentigerous Arm Depth | ankrd33b | HiC_scaffold_11 | CP |
| Dentigerous Arm Depth | ano9 | HiC_scaffold_5 | LL |
| Dentigerous Arm Depth | ap2a2 | HiC_scaffold_5 | LL |
| Dentigerous Arm Depth | apod | HiC_scaffold_11 | CP |
| Dentigerous Arm Depth | arfgef1 | HiC_scaffold_11 | CP |
| Dentigerous Arm Depth | arhgap21 | HiC_scaffold_11 | CP |
| Dentigerous Arm Depth | arhgap29 | HiC_scaffold_11 | CP |
| Dentigerous Arm Depth | armc1 | HiC_scaffold_11 | CP |
| Dentigerous Arm Depth | arpp19 | HiC_scaffold_5 | LL |
| Dentigerous Arm Depth | arx | HiC_scaffold_11 | CP |
| Dentigerous Arm Depth | asap1 | HiC_scaffold_11 | CP |
| Dentigerous Arm Depth | atp8a2 | HiC_scaffold_11 | CP |
| Dentigerous Arm Depth | b2m | HiC_scaffold_11 | CP |
| Dentigerous Arm Depth | b4galt1 | HiC_scaffold_11 | CP |
| Dentigerous Arm Depth | bbs4 | HiC_scaffold_5 | LL |
| Dentigerous Arm Depth | bco1 | HiC_scaffold_11 | CP |
| Dentigerous Arm Depth | bdh1 | HiC_scaffold_11 | CP |
| Dentigerous Arm Depth | bhlhe22 | HiC_scaffold_11 | CP |
| Dentigerous Arm Depth | bloc1s5 | HiC_scaffold_11 | CP |
| Dentigerous Arm Depth | boc | HiC_scaffold_11 | CP |
| Dentigerous Arm Depth | brpf3 | HiC_scaffold_11 | CP |
| Dentigerous Arm Depth | c1qtnf9 | HiC_scaffold_11 | CP |
| Dentigerous Arm Depth | c8g | HiC_scaffold_11 | CP |
| Dentigerous Arm Depth | ca1 | HiC_scaffold_11 | CP |
| Dentigerous Arm Depth | cacnb2 | HiC_scaffold_11 | CP |
| Dentigerous Arm Depth | calml4 | HiC_scaffold_5 | LL |
| Dentigerous Arm Depth | caprin2 | HiC_scaffold_11 | CP |
| Dentigerous Arm Depth | cbln2 | HiC_scaffold_11 | CP |
| Dentigerous Arm Depth | ccdc106 | HiC_scaffold_11 | CP |
| Dentigerous Arm Depth | ccdc58 | HiC_scaffold_11 | CP |
| Dentigerous Arm Depth | ccdc63 | HiC_scaffold_11 | CP |
| Dentigerous Arm Depth | ccl20 | HiC_scaffold_11 | CP |
| Dentigerous Arm Depth | ccne1 | HiC_scaffold_5 | LL |
| Dentigerous Arm Depth | ccr1 | HiC_scaffold_11 | CP |
| Dentigerous Arm Depth | cct5 | HiC_scaffold_11 | CP |
| Dentigerous Arm Depth | cd226 | HiC_scaffold_11 | CP |
| Dentigerous Arm Depth | cd276 | HiC_scaffold_5 | LL |
| Dentigerous Arm Depth | cd38 | HiC_scaffold_11 | CP |
| Dentigerous Arm Depth | cd81 | HiC_scaffold_5 | LL |
| Dentigerous Arm Depth | cdh10 | HiC_scaffold_11 | CP |
| Dentigerous Arm Depth | cdh12 | HiC_scaffold_11 | CP |
| Dentigerous Arm Depth | cdh18 | HiC_scaffold_11 | CP |
| Dentigerous Arm Depth | cdh20 | HiC_scaffold_11 | CP |
| Dentigerous Arm Depth | cdh6 | HiC_scaffold_11 | CP |
| Dentigerous Arm Depth | cdh7 | HiC_scaffold_11 | CP |
| Dentigerous Arm Depth | cdk13 | HiC_scaffold_11 | CP |
| Dentigerous Arm Depth | cdk8 | HiC_scaffold_11 | CP |
| Dentigerous Arm Depth | cdv3 | HiC_scaffold_11 | CP |
| Dentigerous Arm Depth | cebpe | HiC_scaffold_11 | CP |
| Dentigerous Arm Depth | cela2a | HiC_scaffold_11 | CP |
| Dentigerous Arm Depth | chmp4c | HiC_scaffold_11 | CP |
| Dentigerous Arm Depth | chmp5 | HiC_scaffold_11 | CP |
| Dentigerous Arm Depth | chrna7 | HiC_scaffold_5 | LL |
| Dentigerous Arm Depth | chst2 | HiC_scaffold_11 | CP |
| Dentigerous Arm Depth | cldn15 | HiC_scaffold_5 | LL |
| Dentigerous Arm Depth | cln6 | HiC_scaffold_5 | LL |
| Dentigerous Arm Depth | clul1 | HiC_scaffold_11 | CP |
| Dentigerous Arm Depth | cmbl | HiC_scaffold_11 | CP |
| Dentigerous Arm Depth | cngb3 | HiC_scaffold_11 | CP |
| Dentigerous Arm Depth | col11a1 | HiC_scaffold_11 | CP |
| Dentigerous Arm Depth | colec12 | HiC_scaffold_11 | CP |
| Dentigerous Arm Depth | cops5 | HiC_scaffold_11 | CP |
| Dentigerous Arm Depth | cpa6 | HiC_scaffold_11 | CP |
| Dentigerous Arm Depth | cpb1 | HiC_scaffold_11 | CP |
| Dentigerous Arm Depth | cpeb1 | HiC_scaffold_5 | LL |
| Dentigerous Arm Depth | cpne3 | HiC_scaffold_11 | CP |
| Dentigerous Arm Depth | crh | HiC_scaffold_11 | CP |
| Dentigerous Arm Depth | crispld1 | HiC_scaffold_11 | CP |
| Dentigerous Arm Depth | cry-dash | HiC_scaffold_11 | CP |
| Dentigerous Arm Depth | csnk1g1 | HiC_scaffold_5 | LL |
| Dentigerous Arm Depth | csrnp1 | HiC_scaffold_11 | CP |
| Dentigerous Arm Depth | cstb | HiC_scaffold_11 | CP |
| Dentigerous Arm Depth | dcaf11 | HiC_scaffold_11 | CP |
| Dentigerous Arm Depth | dhcr7 | HiC_scaffold_5 | LL |
| Dentigerous Arm Depth | dlec1 | HiC_scaffold_11 | CP |
| Dentigerous Arm Depth | dnajb6 | HiC_scaffold_11 | CP |
| Dentigerous Arm Depth | dnajc13 | HiC_scaffold_11 | CP |
| Dentigerous Arm Depth | dok6 | HiC_scaffold_11 | CP |
| Dentigerous Arm Depth | dph2 | HiC_scaffold_11 | CP |
| Dentigerous Arm Depth | dpp6 | HiC_scaffold_11 | CP |
| Dentigerous Arm Depth | drd3 | HiC_scaffold_11 | CP |
| Dentigerous Arm Depth | drosha | HiC_scaffold_11 | CP |
| Dentigerous Arm Depth | dsel | HiC_scaffold_11 | CP |
| Dentigerous Arm Depth | dusp28 | HiC_scaffold_5 | LL |
| Dentigerous Arm Depth | eef1e1 | HiC_scaffold_11 | CP |
| Dentigerous Arm Depth | ell2 | HiC_scaffold_5 | LL |
| Dentigerous Arm Depth | eloc | HiC_scaffold_11 | CP |
| Dentigerous Arm Depth | emc9 | HiC_scaffold_11 | CP |
| Dentigerous Arm Depth | emilin2 | HiC_scaffold_11 | CP |
| Dentigerous Arm Depth | emp3 | HiC_scaffold_11 | CP |
| Dentigerous Arm Depth | erya | HiC_scaffold_11 | CP |
| Dentigerous Arm Depth | esyt2 | HiC_scaffold_11 | CP |
| Dentigerous Arm Depth | eya1 | HiC_scaffold_11 | CP |
| Dentigerous Arm Depth | f13a1 | HiC_scaffold_11 | CP |
| Dentigerous Arm Depth | f13e9.13 | HiC_scaffold_5 | LL |
| Dentigerous Arm Depth | fam168b | HiC_scaffold_11 | CP |
| Dentigerous Arm Depth | fam214a | HiC_scaffold_5 | LL |
| Dentigerous Arm Depth | fam49b | HiC_scaffold_11 | CP |
| Dentigerous Arm Depth | fam83e | HiC_scaffold_11 | CP |
| Dentigerous Arm Depth | fastkd3 | HiC_scaffold_11 | CP |
| Dentigerous Arm Depth | fbxl7 | HiC_scaffold_11 | CP |
| Dentigerous Arm Depth | fen1 | HiC_scaffold_11 | CP |
| Dentigerous Arm Depth | fitm1 | HiC_scaffold_11 | CP |
| Dentigerous Arm Depth | flt3 | HiC_scaffold_11 | CP |
| Dentigerous Arm Depth | foxh1 | HiC_scaffold_11 | CP |
| Dentigerous Arm Depth | foxj3 | HiC_scaffold_11 | CP |
| Dentigerous Arm Depth | gabarapl2 | HiC_scaffold_5 | LL |
| Dentigerous Arm Depth | gad2 | HiC_scaffold_11 | CP |
| Dentigerous Arm Depth | garem1 | HiC_scaffold_11 | CP |
| Dentigerous Arm Depth | gars | HiC_scaffold_11 | CP |
| Dentigerous Arm Depth | gdap1 | HiC_scaffold_11 | CP |
| Dentigerous Arm Depth | ggh | HiC_scaffold_11 | CP |
| Dentigerous Arm Depth | gimap4 | HiC_scaffold_11 | CP |
| Dentigerous Arm Depth | gli3 | HiC_scaffold_11 | CP |
| Dentigerous Arm Depth | glyctk | HiC_scaffold_11 | CP |
| Dentigerous Arm Depth | gnb5b | HiC_scaffold_5 | LL |
| Dentigerous Arm Depth | gnrhr2 | HiC_scaffold_5 | LL |
| Dentigerous Arm Depth | gorasp1 | HiC_scaffold_11 | CP |
| Dentigerous Arm Depth | gpr12 | HiC_scaffold_11 | CP |
| Dentigerous Arm Depth | gpr141 | HiC_scaffold_11 | CP |
| Dentigerous Arm Depth | gpr17 | HiC_scaffold_11 | CP |
| Dentigerous Arm Depth | gpt2l | HiC_scaffold_11 | CP |
| Dentigerous Arm Depth | gramd1c | HiC_scaffold_11 | CP |
| Dentigerous Arm Depth | gramd2a | HiC_scaffold_5 | LL |
| Dentigerous Arm Depth | gtf3a | HiC_scaffold_11 | CP |
| Dentigerous Arm Depth | hacd1 | HiC_scaffold_11 | CP |
| Dentigerous Arm Depth | hbp1 | HiC_scaffold_11 | CP |
| Dentigerous Arm Depth | hgd | HiC_scaffold_11 | CP |
| Dentigerous Arm Depth | hhatl | HiC_scaffold_11 | CP |
| Dentigerous Arm Depth | hmcn2 | HiC_scaffold_11 | CP |
| Dentigerous Arm Depth | hnf4g | HiC_scaffold_11 | CP |
| Dentigerous Arm Depth | idh2 | HiC_scaffold_5 | LL |
| Dentigerous Arm Depth | il20rb | HiC_scaffold_5 | LL |
| Dentigerous Arm Depth | impa1 | HiC_scaffold_11 | CP |
| Dentigerous Arm Depth | insig1 | HiC_scaffold_11 | CP |
| Dentigerous Arm Depth | insy1 | HiC_scaffold_5 | LL |
| Dentigerous Arm Depth | itga11 | HiC_scaffold_5 | LL |
| Dentigerous Arm Depth | jph1 | HiC_scaffold_11 | CP |
| Dentigerous Arm Depth | kazn | HiC_scaffold_11 | CP |
| Dentigerous Arm Depth | kazna | HiC_scaffold_11 | CP |
| Dentigerous Arm Depth | kbp | HiC_scaffold_11 | CP |
| Dentigerous Arm Depth | kbtbd2 | HiC_scaffold_11 | CP |
| Dentigerous Arm Depth | kcnb2 | HiC_scaffold_11 | CP |
| Dentigerous Arm Depth | kif13b | HiC_scaffold_5 | LL |
| Dentigerous Arm Depth | kif20a | HiC_scaffold_11 | CP |
| Dentigerous Arm Depth | klhl40b | HiC_scaffold_11 | CP |
| Dentigerous Arm Depth | kpna1 | HiC_scaffold_11 | CP |
| Dentigerous Arm Depth | limd2 | HiC_scaffold_11 | CP |
| Dentigerous Arm Depth | lnx2 | HiC_scaffold_11 | CP |
| Dentigerous Arm Depth | loxl1 | HiC_scaffold_5 | LL |
| Dentigerous Arm Depth | lpin2 | HiC_scaffold_11 | CP |
| Dentigerous Arm Depth | lsm5 | HiC_scaffold_11 | CP |
| Dentigerous Arm Depth | lypla1 | HiC_scaffold_11 | CP |
| Dentigerous Arm Depth | lztfl1 | HiC_scaffold_11 | CP |
| Dentigerous Arm Depth | maf1 | HiC_scaffold_11 | CP |
| Dentigerous Arm Depth | mag | HiC_scaffold_11 | CP |
| Dentigerous Arm Depth | mak | HiC_scaffold_11 | CP |
| Dentigerous Arm Depth | map3k15 | HiC_scaffold_11 | CP |
| Dentigerous Arm Depth | mastl | HiC_scaffold_11 | CP |
| Dentigerous Arm Depth | mc4r | HiC_scaffold_11 | CP |
| Dentigerous Arm Depth | mcl1 | HiC_scaffold_5 | LL |
| Dentigerous Arm Depth | mcur1 | HiC_scaffold_11 | CP |
| Dentigerous Arm Depth | med1 | HiC_scaffold_11 | CP |
| Dentigerous Arm Depth | mesd | HiC_scaffold_5 | LL |
| Dentigerous Arm Depth | mettl4 | HiC_scaffold_11 | CP |
| Dentigerous Arm Depth | mllt10 | HiC_scaffold_11 | CP |
| Dentigerous Arm Depth | mon1b | HiC_scaffold_11 | CP |
| Dentigerous Arm Depth | mrc1 | HiC_scaffold_11 | CP |
| Dentigerous Arm Depth | mrpl15 | HiC_scaffold_11 | CP |
| Dentigerous Arm Depth | mrpl46 | HiC_scaffold_5 | LL |
| Dentigerous Arm Depth | msc | HiC_scaffold_11 | CP |
| Dentigerous Arm Depth | msrb2 | HiC_scaffold_11 | CP |
| Dentigerous Arm Depth | mtfr1 | HiC_scaffold_11 | CP |
| Dentigerous Arm Depth | mtmr6 | HiC_scaffold_11 | CP |
| Dentigerous Arm Depth | mtrr | HiC_scaffold_11 | CP |
| Dentigerous Arm Depth | mtss1l | HiC_scaffold_5 | LL |
| Dentigerous Arm Depth | mup20 | HiC_scaffold_11 | CP |
| Dentigerous Arm Depth | mybl1 | HiC_scaffold_11 | CP |
| Dentigerous Arm Depth | myd88 | HiC_scaffold_11 | CP |
| Dentigerous Arm Depth | myh6 | HiC_scaffold_11 | CP |
| Dentigerous Arm Depth | myh7 | HiC_scaffold_11 | CP |
| Dentigerous Arm Depth | myo5a | HiC_scaffold_5 | LL |
| Dentigerous Arm Depth | myo9a | HiC_scaffold_5 | LL |
| Dentigerous Arm Depth | naa50 | HiC_scaffold_11 | CP |
| Dentigerous Arm Depth | ncapg2 | HiC_scaffold_11 | CP |
| Dentigerous Arm Depth | nck1 | HiC_scaffold_11 | CP |
| Dentigerous Arm Depth | ncoa2 | HiC_scaffold_11 | CP |
| Dentigerous Arm Depth | neto1 | HiC_scaffold_11 | CP |
| Dentigerous Arm Depth | nfi1 | HiC_scaffold_11 | CP |
| Dentigerous Arm Depth | nfx1 | HiC_scaffold_11 | CP |
| Dentigerous Arm Depth | ngdn | HiC_scaffold_11 | CP |
| Dentigerous Arm Depth | nlrc3 | HiC_scaffold_11 | CP |
| Dentigerous Arm Depth | nlrp1 | HiC_scaffold_11 | CP |
| Dentigerous Arm Depth | nlrp12 | HiC_scaffold_5 | LL |
| Dentigerous Arm Depth | nom1 | HiC_scaffold_11 | CP |
| Dentigerous Arm Depth | nrn1 | HiC_scaffold_11 | CP |
| Dentigerous Arm Depth | nrros | HiC_scaffold_11 | CP |
| Dentigerous Arm Depth | ntng1 | HiC_scaffold_11 | CP |
| Dentigerous Arm Depth | ntrk3 | HiC_scaffold_5 | LL |
| Dentigerous Arm Depth | nup58 | HiC_scaffold_11 | CP |
| Dentigerous Arm Depth | olfm3 | HiC_scaffold_11 | CP |
| Dentigerous Arm Depth | onecut1 | HiC_scaffold_5 | LL |
| Dentigerous Arm Depth | oplah | HiC_scaffold_11 | CP |
| Dentigerous Arm Depth | oprk1 | HiC_scaffold_11 | CP |
| Dentigerous Arm Depth | otol1 | HiC_scaffold_5 | LL |
| Dentigerous Arm Depth | otulin | HiC_scaffold_11 | CP |
| Dentigerous Arm Depth | oxsr1 | HiC_scaffold_11 | CP |
| Dentigerous Arm Depth | pabpn1 | HiC_scaffold_11 | CP |
| Dentigerous Arm Depth | pan3 | HiC_scaffold_11 | CP |
| Dentigerous Arm Depth | pck2 | HiC_scaffold_11 | CP |
| Dentigerous Arm Depth | pcolce2 | HiC_scaffold_11 | CP |
| Dentigerous Arm Depth | pdcd6 | HiC_scaffold_11 | CP |
| Dentigerous Arm Depth | pde7a | HiC_scaffold_11 | CP |
| Dentigerous Arm Depth | pdia4 | HiC_scaffold_11 | CP |
| Dentigerous Arm Depth | pdk3 | HiC_scaffold_11 | CP |
| Dentigerous Arm Depth | pdpr | HiC_scaffold_5 | LL |
| Dentigerous Arm Depth | pdx1 | HiC_scaffold_11 | CP |
| Dentigerous Arm Depth | pex2 | HiC_scaffold_11 | CP |
| Dentigerous Arm Depth | pgbd2 | HiC_scaffold_11 | CP |
| Dentigerous Arm Depth | phex | HiC_scaffold_11 | CP |
| Dentigerous Arm Depth | phldb2 | HiC_scaffold_11 | CP |
| Dentigerous Arm Depth | pi15a | HiC_scaffold_11 | CP |
| Dentigerous Arm Depth | pigm | HiC_scaffold_11 | CP |
| Dentigerous Arm Depth | pign | HiC_scaffold_11 | CP |
| Dentigerous Arm Depth | pim2 | HiC_scaffold_11 | CP |
| Dentigerous Arm Depth | pkia | HiC_scaffold_11 | CP |
| Dentigerous Arm Depth | pkp3 | HiC_scaffold_5 | LL |
| Dentigerous Arm Depth | pks15/1 | HiC_scaffold_11 | CP |
| Dentigerous Arm Depth | plcd1 | HiC_scaffold_11 | CP |
| Dentigerous Arm Depth | plch2 | HiC_scaffold_11 | CP |
| Dentigerous Arm Depth | plcxd2 | HiC_scaffold_11 | CP |
| Dentigerous Arm Depth | plod2 | HiC_scaffold_11 | CP |
| Dentigerous Arm Depth | plscr2 | HiC_scaffold_11 | CP |
| Dentigerous Arm Depth | pnoc | HiC_scaffold_5 | LL |
| Dentigerous Arm Depth | pola1 | HiC_scaffold_11 | CP |
| Dentigerous Arm Depth | polr1d | HiC_scaffold_11 | CP |
| Dentigerous Arm Depth | pomp | HiC_scaffold_11 | CP |
| Dentigerous Arm Depth | pop4 | HiC_scaffold_5 | LL |
| Dentigerous Arm Depth | pou6f2 | HiC_scaffold_11 | CP |
| Dentigerous Arm Depth | ppcs | HiC_scaffold_11 | CP |
| Dentigerous Arm Depth | ppp1r16a | HiC_scaffold_11 | CP |
| Dentigerous Arm Depth | ppp1r42 | HiC_scaffold_11 | CP |
| Dentigerous Arm Depth | prdm14 | HiC_scaffold_11 | CP |
| Dentigerous Arm Depth | prdm2 | HiC_scaffold_11 | CP |
| Dentigerous Arm Depth | prex2 | HiC_scaffold_11 | CP |
| Dentigerous Arm Depth | prkdc | HiC_scaffold_11 | CP |
| Dentigerous Arm Depth | prlh | HiC_scaffold_11 | CP |
| Dentigerous Arm Depth | prmt6 | HiC_scaffold_11 | CP |
| Dentigerous Arm Depth | proc | HiC_scaffold_5 | LL |
| Dentigerous Arm Depth | prpf4b | HiC_scaffold_11 | CP |
| Dentigerous Arm Depth | prtfdc1 | HiC_scaffold_11 | CP |
| Dentigerous Arm Depth | psma4 | HiC_scaffold_5 | LL |
| Dentigerous Arm Depth | psme1 | HiC_scaffold_11 | CP |
| Dentigerous Arm Depth | psme2 | HiC_scaffold_11 | CP |
| Dentigerous Arm Depth | ptprn2 | HiC_scaffold_11 | CP |
| Dentigerous Arm Depth | puf60 | HiC_scaffold_11 | CP |
| Dentigerous Arm Depth | qtrt2 | HiC_scaffold_11 | CP |
| Dentigerous Arm Depth | rala | HiC_scaffold_11 | CP |
| Dentigerous Arm Depth | ralyl | HiC_scaffold_11 | CP |
| Dentigerous Arm Depth | ramp3 | HiC_scaffold_11 | CP |
| Dentigerous Arm Depth | rbm33 | HiC_scaffold_11 | CP |
| Dentigerous Arm Depth | rbpms2 | HiC_scaffold_5 | LL |
| Dentigerous Arm Depth | rdh10a | HiC_scaffold_11 | CP |
| Dentigerous Arm Depth | rdh12 | HiC_scaffold_11 | CP |
| Dentigerous Arm Depth | rec8 | HiC_scaffold_11 | CP |
| Dentigerous Arm Depth | relch | HiC_scaffold_11 | CP |
| Dentigerous Arm Depth | rgs20 | HiC_scaffold_11 | CP |
| Dentigerous Arm Depth | rgs9bp-b | HiC_scaffold_11 | CP |
| Dentigerous Arm Depth | rmdn1 | HiC_scaffold_11 | CP |
| Dentigerous Arm Depth | rnf152 | HiC_scaffold_11 | CP |
| Dentigerous Arm Depth | rnf6 | HiC_scaffold_11 | CP |
| Dentigerous Arm Depth | rnh1 | HiC_scaffold_11 | CP |
| Dentigerous Arm Depth | rnh1 | HiC_scaffold_5 | LL |
| Dentigerous Arm Depth | rnpc3 | HiC_scaffold_11 | CP |
| Dentigerous Arm Depth | rp1 | HiC_scaffold_11 | CP |
| Dentigerous Arm Depth | rpl21 | HiC_scaffold_11 | CP |
| Dentigerous Arm Depth | rpl7 | HiC_scaffold_11 | CP |
| Dentigerous Arm Depth | rps17 | HiC_scaffold_5 | LL |
| Dentigerous Arm Depth | rps20 | HiC_scaffold_11 | CP |
| Dentigerous Arm Depth | rrs1 | HiC_scaffold_11 | CP |
| Dentigerous Arm Depth | rxfp3 | HiC_scaffold_5 | LL |
| Dentigerous Arm Depth | sag | HiC_scaffold_5 | LL |
| Dentigerous Arm Depth | sbspon | HiC_scaffold_11 | CP |
| Dentigerous Arm Depth | scamp2 | HiC_scaffold_5 | LL |
| Dentigerous Arm Depth | scamp5-a | HiC_scaffold_5 | LL |
| Dentigerous Arm Depth | scrib | HiC_scaffold_11 | CP |
| Dentigerous Arm Depth | sec22c | HiC_scaffold_11 | CP |
| Dentigerous Arm Depth | sec61g | HiC_scaffold_11 | CP |
| Dentigerous Arm Depth | sema4b | HiC_scaffold_5 | LL |
| Dentigerous Arm Depth | sema5a | HiC_scaffold_11 | CP |
| Dentigerous Arm Depth | senp8 | HiC_scaffold_5 | LL |
| Dentigerous Arm Depth | serpinb1 | HiC_scaffold_11 | CP |
| Dentigerous Arm Depth | serpinb10 | HiC_scaffold_11 | CP |
| Dentigerous Arm Depth | serpinb1b | HiC_scaffold_11 | CP |
| Dentigerous Arm Depth | serpinb6 | HiC_scaffold_11 | CP |
| Dentigerous Arm Depth | sgk3 | HiC_scaffold_11 | CP |
| Dentigerous Arm Depth | sh3glb1 | HiC_scaffold_11 | CP |
| Dentigerous Arm Depth | sh3kbp1 | HiC_scaffold_11 | CP |
| Dentigerous Arm Depth | shhb | HiC_scaffold_11 | CP |
| Dentigerous Arm Depth | shisa2 | HiC_scaffold_11 | CP |
| Dentigerous Arm Depth | si:ch211-238a12.2 | HiC_scaffold_5 | LL |
| Dentigerous Arm Depth | siglec1 | HiC_scaffold_11 | CP |
| Dentigerous Arm Depth | siglec10 | HiC_scaffold_11 | CP |
| Dentigerous Arm Depth | siglec13 | HiC_scaffold_11 | CP |
| Dentigerous Arm Depth | siglec14 | HiC_scaffold_11 | CP |
| Dentigerous Arm Depth | siglec9 | HiC_scaffold_11 | CP |
| Dentigerous Arm Depth | ski | HiC_scaffold_11 | CP |
| Dentigerous Arm Depth | skida1 | HiC_scaffold_11 | CP |
| Dentigerous Arm Depth | slc22a13 | HiC_scaffold_11 | CP |
| Dentigerous Arm Depth | slc22a17 | HiC_scaffold_11 | CP |
| Dentigerous Arm Depth | slc35b3 | HiC_scaffold_11 | CP |
| Dentigerous Arm Depth | slc35g2 | HiC_scaffold_11 | CP |
| Dentigerous Arm Depth | slc39a12 | HiC_scaffold_11 | CP |
| Dentigerous Arm Depth | slc4a2 | HiC_scaffold_11 | CP |
| Dentigerous Arm Depth | slc51a | HiC_scaffold_11 | CP |
| Dentigerous Arm Depth | slco5a1 | HiC_scaffold_11 | CP |
| Dentigerous Arm Depth | smarcd3 | HiC_scaffold_11 | CP |
| Dentigerous Arm Depth | smchd1 | HiC_scaffold_11 | CP |
| Dentigerous Arm Depth | snx1 | HiC_scaffold_5 | LL |
| Dentigerous Arm Depth | snx16 | HiC_scaffold_11 | CP |
| Dentigerous Arm Depth | socs6 | HiC_scaffold_11 | CP |
| Dentigerous Arm Depth | sox17a | HiC_scaffold_11 | CP |
| Dentigerous Arm Depth | spag16 | HiC_scaffold_11 | CP |
| Dentigerous Arm Depth | spata13 | HiC_scaffold_11 | CP |
| Dentigerous Arm Depth | spice1 | HiC_scaffold_11 | CP |
| Dentigerous Arm Depth | sppl2a | HiC_scaffold_5 | LL |
| Dentigerous Arm Depth | st14 | HiC_scaffold_11 | CP |
| Dentigerous Arm Depth | stard5 | HiC_scaffold_5 | LL |
| Dentigerous Arm Depth | sun3 | HiC_scaffold_5 | LL |
| Dentigerous Arm Depth | sv2b | HiC_scaffold_5 | LL |
| Dentigerous Arm Depth | tagln3 | HiC_scaffold_11 | CP |
| Dentigerous Arm Depth | tcf24 | HiC_scaffold_11 | CP |
| Dentigerous Arm Depth | tecr | HiC_scaffold_11 | CP |
| Dentigerous Arm Depth | terf1 | HiC_scaffold_11 | CP |
| Dentigerous Arm Depth | tfap2a | HiC_scaffold_11 | CP |
| Dentigerous Arm Depth | tfrc | HiC_scaffold_11 | CP |
| Dentigerous Arm Depth | thap6 | HiC_scaffold_11 | CP |
| Dentigerous Arm Depth | thtpa | HiC_scaffold_11 | CP |
| Dentigerous Arm Depth | tlnrd1 | HiC_scaffold_5 | LL |
| Dentigerous Arm Depth | tm9sf1 | HiC_scaffold_11 | CP |
| Dentigerous Arm Depth | tmem14c | HiC_scaffold_11 | CP |
| Dentigerous Arm Depth | tmem236 | HiC_scaffold_11 | CP |
| Dentigerous Arm Depth | tmem51 | HiC_scaffold_11 | CP |
| Dentigerous Arm Depth | tmem56-b | HiC_scaffold_11 | CP |
| Dentigerous Arm Depth | tmprss7 | HiC_scaffold_11 | CP |
| Dentigerous Arm Depth | tnk2 | HiC_scaffold_11 | CP |
| Dentigerous Arm Depth | topbp1-a | HiC_scaffold_11 | CP |
| Dentigerous Arm Depth | tph1 | HiC_scaffold_5 | LL |
| Dentigerous Arm Depth | tram1l1 | HiC_scaffold_11 | CP |
| Dentigerous Arm Depth | trim55 | HiC_scaffold_11 | CP |
| Dentigerous Arm Depth | trim69 | HiC_scaffold_5 | LL |
| Dentigerous Arm Depth | trip4 | HiC_scaffold_5 | LL |
| Dentigerous Arm Depth | trp53inp1 | HiC_scaffold_5 | LL |
| Dentigerous Arm Depth | trpa1 | HiC_scaffold_11 | CP |
| Dentigerous Arm Depth | trpc1 | HiC_scaffold_11 | CP |
| Dentigerous Arm Depth | trpm7 | HiC_scaffold_5 | LL |
| Dentigerous Arm Depth | tshz3 | HiC_scaffold_5 | LL |
| Dentigerous Arm Depth | tssc4 | HiC_scaffold_5 | LL |
| Dentigerous Arm Depth | tssk1b | HiC_scaffold_11 | CP |
| Dentigerous Arm Depth | tstd3 | HiC_scaffold_11 | CP |
| Dentigerous Arm Depth | txndc5 | HiC_scaffold_11 | CP |
| Dentigerous Arm Depth | tyms | HiC_scaffold_11 | CP |
| Dentigerous Arm Depth | u2surp | HiC_scaffold_11 | CP |
| Dentigerous Arm Depth | ube2w | HiC_scaffold_11 | CP |
| Dentigerous Arm Depth | ube3c | HiC_scaffold_11 | CP |
| Dentigerous Arm Depth | ubl7 | HiC_scaffold_5 | LL |
| Dentigerous Arm Depth | urad | HiC_scaffold_11 | CP |
| Dentigerous Arm Depth | usf3 | HiC_scaffold_11 | CP |
| Dentigerous Arm Depth | usp12 | HiC_scaffold_11 | CP |
| Dentigerous Arm Depth | utp3 | HiC_scaffold_11 | CP |
| Dentigerous Arm Depth | vav3 | HiC_scaffold_11 | CP |
| Dentigerous Arm Depth | vcpip1 | HiC_scaffold_5 | LL |
| Dentigerous Arm Depth | vil1 | HiC_scaffold_11 | CP |
| Dentigerous Arm Depth | vipr1 | HiC_scaffold_11 | CP |
| Dentigerous Arm Depth | vps35 | HiC_scaffold_5 | LL |
| Dentigerous Arm Depth | vstm2a | HiC_scaffold_11 | CP |
| Dentigerous Arm Depth | wasf3 | HiC_scaffold_11 | CP |
| Dentigerous Arm Depth | wdr19 | HiC_scaffold_11 | CP |
| Dentigerous Arm Depth | wdr37 | HiC_scaffold_11 | CP |
| Dentigerous Arm Depth | wdr60 | HiC_scaffold_11 | CP |
| Dentigerous Arm Depth | wwp1 | HiC_scaffold_11 | CP |
| Dentigerous Arm Depth | xcc-b100_1894 | HiC_scaffold_11 | CP |
| Dentigerous Arm Depth | xkr9 | HiC_scaffold_11 | CP |
| Dentigerous Arm Depth | yes1 | HiC_scaffold_11 | CP |
| Dentigerous Arm Depth | yme1l1 | HiC_scaffold_11 | CP |
| Dentigerous Arm Depth | ythdf2 | HiC_scaffold_11 | CP |
| Dentigerous Arm Depth | zc2hc1a | HiC_scaffold_11 | CP |
| Dentigerous Arm Depth | zdhhc23 | HiC_scaffold_11 | CP |
| Dentigerous Arm Depth | zfand1 | HiC_scaffold_11 | CP |
| Dentigerous Arm Depth | zfhx4 | HiC_scaffold_11 | CP |
| Dentigerous Arm Depth | zic1 | HiC_scaffold_11 | CP |
| Dentigerous Arm Depth | zkscan5 | HiC_scaffold_5 | LL |
| Dentigerous Arm Depth | znf235 | HiC_scaffold_11 | CP |
| Dentigerous Arm Depth | znf25 | HiC_scaffold_5 | LL |
| Dentigerous Arm Depth | znf45 | HiC_scaffold_5 | LL |
| Dentigerous Arm Depth | znf507 | HiC_scaffold_5 | LL |
| Dentigerous Arm Depth | znf569 | HiC_scaffold_5 | LL |
| Dentigerous Arm Depth | znf609 | HiC_scaffold_5 | LL |
| Dentigerous Arm Depth | znf652 | HiC_scaffold_11 | CP |
| Dentigerous Arm Depth | znf710 | HiC_scaffold_5 | LL |
| Dentigerous Arm Width | abca1 | HiC_scaffold_24 | LL |
| Dentigerous Arm Width | abca4 | HiC_scaffold_24 | LL |
| Dentigerous Arm Width | abca7 | HiC_scaffold_24 | LL |
| Dentigerous Arm Width | abr | HiC_scaffold_24 | LL |
| Dentigerous Arm Width | acadvl | HiC_scaffold_24 | LL |
| Dentigerous Arm Width | acan | HiC_scaffold_24 | LL |
| Dentigerous Arm Width | acbp4 | HiC_scaffold_24 | LL |
| Dentigerous Arm Width | acy3.2 | HiC_scaffold_24 | LL |
| Dentigerous Arm Width | adamtsl1 | HiC_scaffold_24 | LL |
| Dentigerous Arm Width | adcy2 | HiC_scaffold_24 | LL |
| Dentigerous Arm Width | adgra3 | HiC_scaffold_24 | LL |
| Dentigerous Arm Width | adgrl3 | HiC_scaffold_24 | LL |
| Dentigerous Arm Width | agfg1 | HiC_scaffold_24 | LL |
| Dentigerous Arm Width | alpk1 | HiC_scaffold_58 | LL |
| Dentigerous Arm Width | ami | HiC_scaffold_24 | LL |
| Dentigerous Arm Width | arap2 | HiC_scaffold_24 | LL |
| Dentigerous Arm Width | arhgef11 | HiC_scaffold_24 | LL |
| Dentigerous Arm Width | arl2 | HiC_scaffold_24 | LL |
| Dentigerous Arm Width | atp6ap1 | HiC_scaffold_24 | LL |
| Dentigerous Arm Width | b3gat3 | HiC_scaffold_24 | LL |
| Dentigerous Arm Width | bad | HiC_scaffold_24 | LL |
| Dentigerous Arm Width | bank1 | HiC_scaffold_24 | LL |
| Dentigerous Arm Width | bcl6b | HiC_scaffold_24 | LL |
| Dentigerous Arm Width | brms1la | HiC_scaffold_24 | LL |
| Dentigerous Arm Width | btn2a1 | HiC_scaffold_24 | LL |
| Dentigerous Arm Width | btn2a2 | HiC_scaffold_24 | LL |
| Dentigerous Arm Width | c1ql4 | HiC_scaffold_24 | LL |
| Dentigerous Arm Width | cabp4 | HiC_scaffold_24 | LL |
| Dentigerous Arm Width | capg | HiC_scaffold_24 | LL |
| Dentigerous Arm Width | card6 | HiC_scaffold_24 | LL |
| Dentigerous Arm Width | cbln1 | HiC_scaffold_24 | LL |
| Dentigerous Arm Width | ccdc149b | HiC_scaffold_24 | LL |
| Dentigerous Arm Width | cct7 | HiC_scaffold_24 | LL |
| Dentigerous Arm Width | cd48 | HiC_scaffold_24 | LL |
| Dentigerous Arm Width | cdca9 | HiC_scaffold_24 | LL |
| Dentigerous Arm Width | chordc1 | HiC_scaffold_24 | LL |
| Dentigerous Arm Width | chrnb1 | HiC_scaffold_24 | LL |
| Dentigerous Arm Width | chst12 | HiC_scaffold_24 | LL |
| Dentigerous Arm Width | clcn5 | HiC_scaffold_24 | LL |
| Dentigerous Arm Width | cldn7a | HiC_scaffold_24 | LL |
| Dentigerous Arm Width | cldnd1 | HiC_scaffold_24 | LL |
| Dentigerous Arm Width | clec10a | HiC_scaffold_24 | LL |
| Dentigerous Arm Width | clec12b | HiC_scaffold_24 | LL |
| Dentigerous Arm Width | clec20a | HiC_scaffold_24 | LL |
| Dentigerous Arm Width | cmas | HiC_scaffold_24 | LL |
| Dentigerous Arm Width | cnpy3 | HiC_scaffold_24 | LL |
| Dentigerous Arm Width | coro1b | HiC_scaffold_24 | LL |
| Dentigerous Arm Width | cpras1 | HiC_scaffold_24 | LL |
| Dentigerous Arm Width | cpz | HiC_scaffold_24 | LL |
| Dentigerous Arm Width | ctdnep1a | HiC_scaffold_24 | LL |
| Dentigerous Arm Width | cyld | HiC_scaffold_24 | LL |
| Dentigerous Arm Width | cyp26b1 | HiC_scaffold_24 | LL |
| Dentigerous Arm Width | dctn1 | HiC_scaffold_24 | LL |
| Dentigerous Arm Width | dctn6 | HiC_scaffold_24 | LL |
| Dentigerous Arm Width | ddit4l | HiC_scaffold_24 | LL |
| Dentigerous Arm Width | dennd4c | HiC_scaffold_24 | LL |
| Dentigerous Arm Width | dgkd | HiC_scaffold_24 | LL |
| Dentigerous Arm Width | dmrta1 | HiC_scaffold_24 | LL |
| Dentigerous Arm Width | dnai2 | HiC_scaffold_24 | LL |
| Dentigerous Arm Width | dok1 | HiC_scaffold_24 | LL |
| Dentigerous Arm Width | dok7 | HiC_scaffold_24 | LL |
| Dentigerous Arm Width | dtx4 | HiC_scaffold_24 | LL |
| Dentigerous Arm Width | dysf | HiC_scaffold_24 | LL |
| Dentigerous Arm Width | eif5a | HiC_scaffold_24 | LL |
| Dentigerous Arm Width | elavl2 | HiC_scaffold_24 | LL |
| Dentigerous Arm Width | elp5 | HiC_scaffold_24 | LL |
| Dentigerous Arm Width | emc4 | HiC_scaffold_24 | LL |
| Dentigerous Arm Width | endod1 | HiC_scaffold_24 | LL |
| Dentigerous Arm Width | epd | HiC_scaffold_58 | LL |
| Dentigerous Arm Width | epd2 | HiC_scaffold_58 | LL |
| Dentigerous Arm Width | epo | HiC_scaffold_24 | LL |
| Dentigerous Arm Width | ern1 | HiC_scaffold_24 | LL |
| Dentigerous Arm Width | etnppl | HiC_scaffold_24 | LL |
| Dentigerous Arm Width | fabp2 | HiC_scaffold_24 | LL |
| Dentigerous Arm Width | fcgr2 | HiC_scaffold_24 | LL |
| Dentigerous Arm Width | fn1 | HiC_scaffold_24 | LL |
| Dentigerous Arm Width | fxr1 | HiC_scaffold_58 | LL |
| Dentigerous Arm Width | g0s2 | HiC_scaffold_24 | LL |
| Dentigerous Arm Width | gab1 | HiC_scaffold_24 | LL |
| Dentigerous Arm Width | gabarap | HiC_scaffold_24 | LL |
| Dentigerous Arm Width | gba3 | HiC_scaffold_24 | LL |
| Dentigerous Arm Width | gdi1 | HiC_scaffold_24 | LL |
| Dentigerous Arm Width | gimap3 | HiC_scaffold_24 | LL |
| Dentigerous Arm Width | gimap4 | HiC_scaffold_24 | LL |
| Dentigerous Arm Width | gimap5 | HiC_scaffold_24 | LL |
| Dentigerous Arm Width | gimap6 | HiC_scaffold_24 | LL |
| Dentigerous Arm Width | gimap7 | HiC_scaffold_24 | LL |
| Dentigerous Arm Width | gimap8 | HiC_scaffold_24 | LL |
| Dentigerous Arm Width | gpha2 | HiC_scaffold_24 | LL |
| Dentigerous Arm Width | gpr12 | HiC_scaffold_24 | LL |
| Dentigerous Arm Width | gpr26 | HiC_scaffold_24 | LL |
| Dentigerous Arm Width | gpr4 | HiC_scaffold_24 | LL |
| Dentigerous Arm Width | gps2 | HiC_scaffold_24 | LL |
| Dentigerous Arm Width | gvin1 | HiC_scaffold_24 | LL |
| Dentigerous Arm Width | haus4 | HiC_scaffold_58 | LL |
| Dentigerous Arm Width | hdlbp | HiC_scaffold_24 | LL |
| Dentigerous Arm Width | hgfac | HiC_scaffold_24 | LL |
| Dentigerous Arm Width | hmx1 | HiC_scaffold_24 | LL |
| Dentigerous Arm Width | hmx2 | HiC_scaffold_24 | LL |
| Dentigerous Arm Width | hnrnpc | HiC_scaffold_24 | LL |
| Dentigerous Arm Width | hspa12b | HiC_scaffold_24 | LL |
| Dentigerous Arm Width | htr2a | HiC_scaffold_24 | LL |
| Dentigerous Arm Width | irs1-b | HiC_scaffold_24 | LL |
| Dentigerous Arm Width | itih6 | HiC_scaffold_24 | LL |
| Dentigerous Arm Width | kcnip4 | HiC_scaffold_24 | LL |
| Dentigerous Arm Width | kdm6b | HiC_scaffold_24 | LL |
| Dentigerous Arm Width | kirrel1 | HiC_scaffold_58 | LL |
| Dentigerous Arm Width | klhl33 | HiC_scaffold_24 | LL |
| Dentigerous Arm Width | lgi2 | HiC_scaffold_24 | LL |
| Dentigerous Arm Width | lpcat4 | HiC_scaffold_24 | LL |
| Dentigerous Arm Width | lrfn2 | HiC_scaffold_24 | LL |
| Dentigerous Arm Width | ltb4r | HiC_scaffold_24 | LL |
| Dentigerous Arm Width | ltb4r2 | HiC_scaffold_24 | LL |
| Dentigerous Arm Width | lurap1l | HiC_scaffold_24 | LL |
| Dentigerous Arm Width | majin | HiC_scaffold_24 | LL |
| Dentigerous Arm Width | mark2 | HiC_scaffold_24 | LL |
| Dentigerous Arm Width | mb21d2 | HiC_scaffold_24 | LL |
| Dentigerous Arm Width | mpdz | HiC_scaffold_24 | LL |
| Dentigerous Arm Width | mrc1 | HiC_scaffold_24 | LL |
| Dentigerous Arm Width | mrc2 | HiC_scaffold_24 | LL |
| Dentigerous Arm Width | mrpl48 | HiC_scaffold_58 | LL |
| Dentigerous Arm Width | msantd1 | HiC_scaffold_24 | LL |
| Dentigerous Arm Width | msmeg_2408 | HiC_scaffold_24 | LL |
| Dentigerous Arm Width | mus81 | HiC_scaffold_24 | LL |
| Dentigerous Arm Width | myadm | HiC_scaffold_24 | LL |
| Dentigerous Arm Width | myoz2 | HiC_scaffold_24 | LL |
| Dentigerous Arm Width | n4bp1 | HiC_scaffold_24 | LL |
| Dentigerous Arm Width | naa40 | HiC_scaffold_24 | LL |
| Dentigerous Arm Width | nagk | HiC_scaffold_24 | LL |
| Dentigerous Arm Width | ndrg2 | HiC_scaffold_24 | LL |
| Dentigerous Arm Width | ndufs2 | HiC_scaffold_58 | LL |
| Dentigerous Arm Width | nectin4 | HiC_scaffold_58 | LL |
| Dentigerous Arm Width | neurl4 | HiC_scaffold_24 | LL |
| Dentigerous Arm Width | nfib | HiC_scaffold_24 | LL |
| Dentigerous Arm Width | nlgn4x | HiC_scaffold_24 | LL |
| Dentigerous Arm Width | nlrc3 | HiC_scaffold_24 | LL |
| Dentigerous Arm Width | nlrp1 | HiC_scaffold_24 | LL |
| Dentigerous Arm Width | nwd2 | HiC_scaffold_24 | LL |
| Dentigerous Arm Width | obscn | HiC_scaffold_24 | LL |
| Dentigerous Arm Width | oga | HiC_scaffold_24 | LL |
| Dentigerous Arm Width | or131-2 | HiC_scaffold_24 | LL |
| Dentigerous Arm Width | osbp | HiC_scaffold_24 | LL |
| Dentigerous Arm Width | ostc | HiC_scaffold_24 | LL |
| Dentigerous Arm Width | otub1 | HiC_scaffold_58 | LL |
| Dentigerous Arm Width | ovol1 | HiC_scaffold_24 | LL |
| Dentigerous Arm Width | p2ry1 | HiC_scaffold_24 | LL |
| Dentigerous Arm Width | paip2b | HiC_scaffold_24 | LL |
| Dentigerous Arm Width | parp14 | HiC_scaffold_24 | LL |
| Dentigerous Arm Width | parp15 | HiC_scaffold_24 | LL |
| Dentigerous Arm Width | parp9 | HiC_scaffold_24 | LL |
| Dentigerous Arm Width | pcdh7 | HiC_scaffold_24 | LL |
| Dentigerous Arm Width | pced1a | HiC_scaffold_24 | LL |
| Dentigerous Arm Width | pcolce2 | HiC_scaffold_24 | LL |
| Dentigerous Arm Width | pea15 | HiC_scaffold_58 | LL |
| Dentigerous Arm Width | per1 | HiC_scaffold_24 | LL |
| Dentigerous Arm Width | pfkfb1 | HiC_scaffold_24 | LL |
| Dentigerous Arm Width | phf23b | HiC_scaffold_24 | LL |
| Dentigerous Arm Width | pla2r1 | HiC_scaffold_24 | LL |
| Dentigerous Arm Width | plac8l1 | HiC_scaffold_24 | LL |
| Dentigerous Arm Width | plin2 | HiC_scaffold_24 | LL |
| Dentigerous Arm Width | plscr2 | HiC_scaffold_24 | LL |
| Dentigerous Arm Width | polr2a | HiC_scaffold_24 | LL |
| Dentigerous Arm Width | pop7 | HiC_scaffold_24 | LL |
| Dentigerous Arm Width | ppargc1a | HiC_scaffold_24 | LL |
| Dentigerous Arm Width | ppp1r14b | HiC_scaffold_24 | LL |
| Dentigerous Arm Width | ppp2r5b | HiC_scaffold_24 | LL |
| Dentigerous Arm Width | ppp3ca | HiC_scaffold_24 | LL |
| Dentigerous Arm Width | prox1 | HiC_scaffold_24 | LL |
| Dentigerous Arm Width | prss27 | HiC_scaffold_24 | LL |
| Dentigerous Arm Width | prss8 | HiC_scaffold_24 | LL |
| Dentigerous Arm Width | ptprd | HiC_scaffold_24 | LL |
| Dentigerous Arm Width | rab38 | HiC_scaffold_24 | LL |
| Dentigerous Arm Width | rab39b | HiC_scaffold_24 | LL |
| Dentigerous Arm Width | rasgrp2-b | HiC_scaffold_58 | LL |
| Dentigerous Arm Width | rbm4b | HiC_scaffold_58 | LL |
| Dentigerous Arm Width | rbpms | HiC_scaffold_24 | LL |
| Dentigerous Arm Width | rcor2 | HiC_scaffold_24 | LL |
| Dentigerous Arm Width | ripk4 | HiC_scaffold_24 | LL |
| Dentigerous Arm Width | rnf183 | HiC_scaffold_24 | LL |
| Dentigerous Arm Width | rnf223 | HiC_scaffold_24 | LL |
| Dentigerous Arm Width | rpl34 | HiC_scaffold_24 | LL |
| Dentigerous Arm Width | sec24d | HiC_scaffold_24 | LL |
| Dentigerous Arm Width | sema4f | HiC_scaffold_24 | LL |
| Dentigerous Arm Width | sgcz | HiC_scaffold_24 | LL |
| Dentigerous Arm Width | shbg | HiC_scaffold_24 | LL |
| Dentigerous Arm Width | slamf9 | HiC_scaffold_24 | LL |
| Dentigerous Arm Width | slc12a3 | HiC_scaffold_24 | LL |
| Dentigerous Arm Width | slc12a6 | HiC_scaffold_24 | LL |
| Dentigerous Arm Width | slc14a2 | HiC_scaffold_24 | LL |
| Dentigerous Arm Width | slc16a13 | HiC_scaffold_24 | LL |
| Dentigerous Arm Width | slc2a4 | HiC_scaffold_24 | LL |
| Dentigerous Arm Width | slc8a1 | HiC_scaffold_24 | LL |
| Dentigerous Arm Width | sned1 | HiC_scaffold_24 | LL |
| Dentigerous Arm Width | snx15 | HiC_scaffold_24 | LL |
| Dentigerous Arm Width | spag17 | HiC_scaffold_24 | LL |
| Dentigerous Arm Width | stk26 | HiC_scaffold_24 | LL |
| Dentigerous Arm Width | supt16h | HiC_scaffold_24 | LL |
| Dentigerous Arm Width | synpo2 | HiC_scaffold_24 | LL |
| Dentigerous Arm Width | syt4 | HiC_scaffold_24 | LL |
| Dentigerous Arm Width | taf8 | HiC_scaffold_24 | LL |
| Dentigerous Arm Width | tdrd7b | HiC_scaffold_24 | LL |
| Dentigerous Arm Width | tgas006m08.1 | HiC_scaffold_24 | LL |
| Dentigerous Arm Width | tkfc | HiC_scaffold_24 | LL |
| Dentigerous Arm Width | tmem151b | HiC_scaffold_24 | LL |
| Dentigerous Arm Width | tmem179b | HiC_scaffold_24 | LL |
| Dentigerous Arm Width | tmem55bb | HiC_scaffold_24 | LL |
| Dentigerous Arm Width | tmem88 | HiC_scaffold_24 | LL |
| Dentigerous Arm Width | tmprss15 | HiC_scaffold_24 | LL |
| Dentigerous Arm Width | tnc | HiC_scaffold_24 | LL |
| Dentigerous Arm Width | tnfsf10 | HiC_scaffold_24 | LL |
| Dentigerous Arm Width | tnk2 | HiC_scaffold_24 | LL |
| Dentigerous Arm Width | tox4-b | HiC_scaffold_24 | LL |
| Dentigerous Arm Width | tp53 | HiC_scaffold_24 | LL |
| Dentigerous Arm Width | trbv2 | HiC_scaffold_58 | LL |
| Dentigerous Arm Width | trim27 | HiC_scaffold_24 | LL |
| Dentigerous Arm Width | trim39 | HiC_scaffold_24 | LL |
| Dentigerous Arm Width | trip6 | HiC_scaffold_24 | LL |
| Dentigerous Arm Width | trmt44 | HiC_scaffold_24 | LL |
| Dentigerous Arm Width | tyrp1 | HiC_scaffold_24 | LL |
| Dentigerous Arm Width | ufsp1 | HiC_scaffold_24 | LL |
| Dentigerous Arm Width | ugt2b20 | HiC_scaffold_24 | LL |
| Dentigerous Arm Width | ugt2c1 | HiC_scaffold_24 | LL |
| Dentigerous Arm Width | urgcp | HiC_scaffold_24 | LL |
| Dentigerous Arm Width | vangl2 | HiC_scaffold_24 | LL |
| Dentigerous Arm Width | vbp1 | HiC_scaffold_24 | LL |
| Dentigerous Arm Width | wasf3 | HiC_scaffold_24 | LL |
| Dentigerous Arm Width | ybx1 | HiC_scaffold_24 | LL |
| Dentigerous Arm Width | zbtb38 | HiC_scaffold_24 | LL |
| Dentigerous Arm Width | zdhhc21 | HiC_scaffold_24 | LL |
| Dentigerous Arm Width | zdhhc3 | HiC_scaffold_24 | LL |
| Dentigerous Arm Width | znf638 | HiC_scaffold_24 | LL |
| Jaw closing In-Lever | a1cf | HiC_scaffold_8 | LL |
| Jaw closing In-Lever | abcc3 | HiC_scaffold_8 | LL |
| Jaw closing In-Lever | acadsb | HiC_scaffold_8 | LL |
| Jaw closing In-Lever | adam12 | HiC_scaffold_8 | LL |
| Jaw closing In-Lever | adap1 | HiC_scaffold_8 | LL |
| Jaw closing In-Lever | ado | HiC_scaffold_8 | LL |
| Jaw closing In-Lever | amdhd2 | HiC_scaffold_8 | LL |
| Jaw closing In-Lever | antxr1 | HiC_scaffold_8 | LL |
| Jaw closing In-Lever | aqp8 | HiC_scaffold_8 | LL |
| Jaw closing In-Lever | arf1 | HiC_scaffold_8 | LL |
| Jaw closing In-Lever | arhgap17 | HiC_scaffold_8 | LL |
| Jaw closing In-Lever | arhgap24 | HiC_scaffold_8 | LL |
| Jaw closing In-Lever | asb12 | HiC_scaffold_8 | LL |
| Jaw closing In-Lever | atp6v0a1 | HiC_scaffold_8 | LL |
| Jaw closing In-Lever | atpaf2 | HiC_scaffold_8 | LL |
| Jaw closing In-Lever | baiap2l1 | HiC_scaffold_8 | LL |
| Jaw closing In-Lever | bbs1 | HiC_scaffold_8 | LL |
| Jaw closing In-Lever | bccip | HiC_scaffold_8 | LL |
| Jaw closing In-Lever | bms1 | HiC_scaffold_8 | LL |
| Jaw closing In-Lever | bricd5 | HiC_scaffold_8 | LL |
| Jaw closing In-Lever | btbd17 | HiC_scaffold_8 | LL |
| Jaw closing In-Lever | bub3 | HiC_scaffold_8 | LL |
| Jaw closing In-Lever | cacna1g | HiC_scaffold_8 | LL |
| Jaw closing In-Lever | cavin1 | HiC_scaffold_8 | LL |
| Jaw closing In-Lever | cbx7 | HiC_scaffold_8 | LL |
| Jaw closing In-Lever | cd163 | HiC_scaffold_8 | LL |
| Jaw closing In-Lever | cdr2l | HiC_scaffold_8 | LL |
| Jaw closing In-Lever | chadl | HiC_scaffold_8 | LL |
| Jaw closing In-Lever | chrm3 | HiC_scaffold_8 | LL |
| Jaw closing In-Lever | chst15 | HiC_scaffold_8 | LL |
| Jaw closing In-Lever | coe3 | HiC_scaffold_8 | LL |
| Jaw closing In-Lever | col14a1 | HiC_scaffold_8 | LL |
| Jaw closing In-Lever | cox19 | HiC_scaffold_8 | LL |
| Jaw closing In-Lever | cpped1 | HiC_scaffold_8 | LL |
| Jaw closing In-Lever | cpxm2 | HiC_scaffold_8 | LL |
| Jaw closing In-Lever | cxcr6 | HiC_scaffold_8 | LL |
| Jaw closing In-Lever | cybc1 | HiC_scaffold_8 | LL |
| Jaw closing In-Lever | d7ertd443e | HiC_scaffold_8 | LL |
| Jaw closing In-Lever | dhrs7ca | HiC_scaffold_8 | LL |
| Jaw closing In-Lever | dnah9 | HiC_scaffold_8 | LL |
| Jaw closing In-Lever | dpysl2 | HiC_scaffold_8 | LL |
| Jaw closing In-Lever | egr2b | HiC_scaffold_8 | LL |
| Jaw closing In-Lever | elovl6 | HiC_scaffold_8 | LL |
| Jaw closing In-Lever | endod1 | HiC_scaffold_8 | LL |
| Jaw closing In-Lever | ep300 | HiC_scaffold_8 | LL |
| Jaw closing In-Lever | ercc4 | HiC_scaffold_8 | LL |
| Jaw closing In-Lever | exoc6 | HiC_scaffold_8 | LL |
| Jaw closing In-Lever | fads6 | HiC_scaffold_8 | LL |
| Jaw closing In-Lever | fam13a | HiC_scaffold_8 | LL |
| Jaw closing In-Lever | fam171a2 | HiC_scaffold_8 | LL |
| Jaw closing In-Lever | fam53b | HiC_scaffold_8 | LL |
| Jaw closing In-Lever | fasn | HiC_scaffold_8 | LL |
| Jaw closing In-Lever | fdxr | HiC_scaffold_8 | LL |
| Jaw closing In-Lever | fmnl1 | HiC_scaffold_8 | LL |
| Jaw closing In-Lever | foxj1b | HiC_scaffold_8 | LL |
| Jaw closing In-Lever | foxk2 | HiC_scaffold_8 | LL |
| Jaw closing In-Lever | foxl1 | HiC_scaffold_8 | LL |
| Jaw closing In-Lever | frmpd2 | HiC_scaffold_8 | LL |
| Jaw closing In-Lever | galk1 | HiC_scaffold_8 | LL |
| Jaw closing In-Lever | galr2 | HiC_scaffold_8 | LL |
| Jaw closing In-Lever | gas7 | HiC_scaffold_8 | LL |
| Jaw closing In-Lever | gdf10 | HiC_scaffold_8 | LL |
| Jaw closing In-Lever | get4 | HiC_scaffold_8 | LL |
| Jaw closing In-Lever | gid4 | HiC_scaffold_8 | LL |
| Jaw closing In-Lever | gimap4 | HiC_scaffold_8 | LL |
| Jaw closing In-Lever | glp2r | HiC_scaffold_8 | LL |
| Jaw closing In-Lever | gpr142 | HiC_scaffold_8 | LL |
| Jaw closing In-Lever | gpr26 | HiC_scaffold_8 | LL |
| Jaw closing In-Lever | gprc5c | HiC_scaffold_8 | LL |
| Jaw closing In-Lever | grb10 | HiC_scaffold_8 | LL |
| Jaw closing In-Lever | grid2ip | HiC_scaffold_8 | LL |
| Jaw closing In-Lever | grn | HiC_scaffold_8 | LL |
| Jaw closing In-Lever | gsg1l | HiC_scaffold_8 | LL |
| Jaw closing In-Lever | hba1 | HiC_scaffold_8 | LL |
| Jaw closing In-Lever | hbb1 | HiC_scaffold_8 | LL |
| Jaw closing In-Lever | hexd | HiC_scaffold_8 | LL |
| Jaw closing In-Lever | hhex | HiC_scaffold_8 | LL |
| Jaw closing In-Lever | hid1 | HiC_scaffold_8 | LL |
| Jaw closing In-Lever | hmx3b | HiC_scaffold_8 | LL |
| Jaw closing In-Lever | hpdl | HiC_scaffold_8 | LL |
| Jaw closing In-Lever | hs3st3a1 | HiC_scaffold_8 | LL |
| Jaw closing In-Lever | hs3st3b1 | HiC_scaffold_8 | LL |
| Jaw closing In-Lever | itgb4 | HiC_scaffold_8 | LL |
| Jaw closing In-Lever | jakmip3 | HiC_scaffold_8 | LL |
| Jaw closing In-Lever | jmjd8 | HiC_scaffold_8 | LL |
| Jaw closing In-Lever | kcnj16 | HiC_scaffold_8 | LL |
| Jaw closing In-Lever | kcnj2 | HiC_scaffold_8 | LL |
| Jaw closing In-Lever | kdelr2 | HiC_scaffold_8 | LL |
| Jaw closing In-Lever | kif20b | HiC_scaffold_8 | LL |
| Jaw closing In-Lever | lcmt1 | HiC_scaffold_8 | LL |
| Jaw closing In-Lever | lect2 | HiC_scaffold_8 | LL |
| Jaw closing In-Lever | lhpp | HiC_scaffold_8 | LL |
| Jaw closing In-Lever | llgl2 | HiC_scaffold_8 | LL |
| Jaw closing In-Lever | lmf1 | HiC_scaffold_8 | LL |
| Jaw closing In-Lever | lrrc45 | HiC_scaffold_8 | LL |
| Jaw closing In-Lever | map2k4 | HiC_scaffold_8 | LL |
| Jaw closing In-Lever | map2k6 | HiC_scaffold_8 | LL |
| Jaw closing In-Lever | map3k14 | HiC_scaffold_8 | LL |
| Jaw closing In-Lever | mapk8b | HiC_scaffold_8 | LL |
| Jaw closing In-Lever | mbtd1 | HiC_scaffold_8 | LL |
| Jaw closing In-Lever | meiob | HiC_scaffold_8 | LL |
| Jaw closing In-Lever | mettl9 | HiC_scaffold_8 | LL |
| Jaw closing In-Lever | mfap4 | HiC_scaffold_8 | LL |
| Jaw closing In-Lever | mlst8 | HiC_scaffold_8 | LL |
| Jaw closing In-Lever | mmp21 | HiC_scaffold_8 | LL |
| Jaw closing In-Lever | mms19 | HiC_scaffold_8 | LL |
| Jaw closing In-Lever | mprip | HiC_scaffold_8 | LL |
| Jaw closing In-Lever | mrpl27 | HiC_scaffold_8 | LL |
| Jaw closing In-Lever | mrpl38 | HiC_scaffold_8 | LL |
| Jaw closing In-Lever | mrtfa | HiC_scaffold_8 | LL |
| Jaw closing In-Lever | mrtfb | HiC_scaffold_8 | LL |
| Jaw closing In-Lever | mtfr1l | HiC_scaffold_8 | LL |
| Jaw closing In-Lever | mtr | HiC_scaffold_8 | LL |
| Jaw closing In-Lever | myh16 | HiC_scaffold_8 | LL |
| Jaw closing In-Lever | myh7 | HiC_scaffold_8 | LL |
| Jaw closing In-Lever | myo15a | HiC_scaffold_8 | LL |
| Jaw closing In-Lever | myo15b | HiC_scaffold_8 | LL |
| Jaw closing In-Lever | myocd | HiC_scaffold_8 | LL |
| Jaw closing In-Lever | narf | HiC_scaffold_8 | LL |
| Jaw closing In-Lever | ndufaf4 | HiC_scaffold_8 | LL |
| Jaw closing In-Lever | nme2 | HiC_scaffold_8 | LL |
| Jaw closing In-Lever | noxo1 | HiC_scaffold_8 | LL |
| Jaw closing In-Lever | nploc4 | HiC_scaffold_8 | LL |
| Jaw closing In-Lever | nptx2 | HiC_scaffold_8 | LL |
| Jaw closing In-Lever | nrbf2 | HiC_scaffold_8 | LL |
| Jaw closing In-Lever | nt5m | HiC_scaffold_8 | LL |
| Jaw closing In-Lever | oat | HiC_scaffold_8 | LL |
| Jaw closing In-Lever | pgp | HiC_scaffold_8 | LL |
| Jaw closing In-Lever | phf5a | HiC_scaffold_8 | LL |
| Jaw closing In-Lever | phyhipl | HiC_scaffold_8 | LL |
| Jaw closing In-Lever | pim3 | HiC_scaffold_8 | LL |
| Jaw closing In-Lever | plau | HiC_scaffold_8 | LL |
| Jaw closing In-Lever | plcd3a | HiC_scaffold_8 | LL |
| Jaw closing In-Lever | plcxd1 | HiC_scaffold_8 | LL |
| Jaw closing In-Lever | polr3d | HiC_scaffold_8 | LL |
| Jaw closing In-Lever | ppp1r3cb | HiC_scaffold_8 | LL |
| Jaw closing In-Lever | prkg1 | HiC_scaffold_8 | LL |
| Jaw closing In-Lever | prss8 | HiC_scaffold_8 | LL |
| Jaw closing In-Lever | pstk | HiC_scaffold_8 | LL |
| Jaw closing In-Lever | pts | HiC_scaffold_8 | LL |
| Jaw closing In-Lever | rab37 | HiC_scaffold_8 | LL |
| Jaw closing In-Lever | rab3gap1 | HiC_scaffold_8 | LL |
| Jaw closing In-Lever | ramp1 | HiC_scaffold_8 | LL |
| Jaw closing In-Lever | rangap1 | HiC_scaffold_8 | LL |
| Jaw closing In-Lever | rasd1 | HiC_scaffold_8 | LL |
| Jaw closing In-Lever | rbp3 | HiC_scaffold_8 | LL |
| Jaw closing In-Lever | reep3 | HiC_scaffold_8 | LL |
| Jaw closing In-Lever | rhbdf1 | HiC_scaffold_8 | LL |
| Jaw closing In-Lever | sap30bp | HiC_scaffold_8 | LL |
| Jaw closing In-Lever | sbk1 | HiC_scaffold_8 | LL |
| Jaw closing In-Lever | shisa6 | HiC_scaffold_8 | LL |
| Jaw closing In-Lever | shisa9 | HiC_scaffold_8 | LL |
| Jaw closing In-Lever | shisa9a | HiC_scaffold_8 | LL |
| Jaw closing In-Lever | slc16a12b | HiC_scaffold_8 | LL |
| Jaw closing In-Lever | slc2a11 | HiC_scaffold_8 | LL |
| Jaw closing In-Lever | slc9a3r1 | HiC_scaffold_8 | LL |
| Jaw closing In-Lever | snx29 | HiC_scaffold_8 | LL |
| Jaw closing In-Lever | sox8 | HiC_scaffold_8 | LL |
| Jaw closing In-Lever | spag9 | HiC_scaffold_8 | LL |
| Jaw closing In-Lever | srcin1 | HiC_scaffold_8 | LL |
| Jaw closing In-Lever | sstr2 | HiC_scaffold_8 | LL |
| Jaw closing In-Lever | st6galnac2 | HiC_scaffold_8 | LL |
| Jaw closing In-Lever | stat5b | HiC_scaffold_8 | LL |
| Jaw closing In-Lever | sult2b1 | HiC_scaffold_8 | LL |
| Jaw closing In-Lever | tcerg1l | HiC_scaffold_8 | LL |
| Jaw closing In-Lever | tdrkh | HiC_scaffold_8 | LL |
| Jaw closing In-Lever | tex2 | HiC_scaffold_8 | LL |
| Jaw closing In-Lever | tgas113e22.1 | HiC_scaffold_8 | LL |
| Jaw closing In-Lever | thap10 | HiC_scaffold_8 | LL |
| Jaw closing In-Lever | tmem130 | HiC_scaffold_8 | LL |
| Jaw closing In-Lever | tmem238 | HiC_scaffold_8 | LL |
| Jaw closing In-Lever | tmem94 | HiC_scaffold_8 | LL |
| Jaw closing In-Lever | tmprss5 | HiC_scaffold_8 | LL |
| Jaw closing In-Lever | tnfrsf13b | HiC_scaffold_8 | LL |
| Jaw closing In-Lever | tom1l2 | HiC_scaffold_8 | LL |
| Jaw closing In-Lever | trim16 | HiC_scaffold_8 | LL |
| Jaw closing In-Lever | trim39 | HiC_scaffold_8 | LL |
| Jaw closing In-Lever | trim65 | HiC_scaffold_8 | LL |
| Jaw closing In-Lever | trrap | HiC_scaffold_8 | LL |
| Jaw closing In-Lever | trub1 | HiC_scaffold_8 | LL |
| Jaw closing In-Lever | tuba1c | HiC_scaffold_8 | LL |
| Jaw closing In-Lever | tvp23b | HiC_scaffold_8 | LL |
| Jaw closing In-Lever | ubald1 | HiC_scaffold_8 | LL |
| Jaw closing In-Lever | ubtd1 | HiC_scaffold_8 | LL |
| Jaw closing In-Lever | ugt2c1 | HiC_scaffold_8 | LL |
| Jaw closing In-Lever | unc13d | HiC_scaffold_8 | LL |
| Jaw closing In-Lever | unk | HiC_scaffold_8 | LL |
| Jaw closing In-Lever | uros | HiC_scaffold_8 | LL |
| Jaw closing In-Lever | ush1g | HiC_scaffold_8 | LL |
| Jaw closing In-Lever | usp22 | HiC_scaffold_8 | LL |
| Jaw closing In-Lever | uts2r | HiC_scaffold_8 | LL |
| Jaw closing In-Lever | wbp2 | HiC_scaffold_8 | LL |
| Jaw closing In-Lever | wfikkn2 | HiC_scaffold_8 | LL |
| Jaw closing In-Lever | xrcc6 | HiC_scaffold_8 | LL |
| Jaw closing In-Lever | zc3h7b | HiC_scaffold_8 | LL |
| Jaw closing In-Lever | znf235 | HiC_scaffold_8 | LL |
| Jaw closing In-Lever | znf569 | HiC_scaffold_8 | LL |
| Jaw closing In-Lever | znf84 | HiC_scaffold_8 | LL |
| Jaw closing In-Lever | zranb1 | HiC_scaffold_8 | LL |
| Maxillary Head Protrusion | sep7 | HiC_scaffold_53 | LL |
| Maxillary Head Protrusion | abcb1 | HiC_scaffold_53 | LL |
| Maxillary Head Protrusion | abhd16a | HiC_scaffold_53 | LL |
| Maxillary Head Protrusion | acan | HiC_scaffold_53 | LL |
| Maxillary Head Protrusion | adam22 | HiC_scaffold_53 | LL |
| Maxillary Head Protrusion | adamts16 | HiC_scaffold_53 | LL |
| Maxillary Head Protrusion | adar | HiC_scaffold_53 | LL |
| Maxillary Head Protrusion | adcy2 | HiC_scaffold_53 | LL |
| Maxillary Head Protrusion | agmo | HiC_scaffold_53 | LL |
| Maxillary Head Protrusion | ago1 | HiC_scaffold_53 | LL |
| Maxillary Head Protrusion | ago3 | HiC_scaffold_53 | LL |
| Maxillary Head Protrusion | aicda | HiC_scaffold_53 | LL |
| Maxillary Head Protrusion | aif1l | HiC_scaffold_53 | LL |
| Maxillary Head Protrusion | alg2 | HiC_scaffold_53 | LL |
| Maxillary Head Protrusion | ankib1 | HiC_scaffold_53 | LL |
| Maxillary Head Protrusion | ankmy2 | HiC_scaffold_53 | LL |
| Maxillary Head Protrusion | ankrd28 | HiC_scaffold_53 | LL |
| Maxillary Head Protrusion | apoa1 | HiC_scaffold_53 | LL |
| Maxillary Head Protrusion | apoa4 | HiC_scaffold_53 | LL |
| Maxillary Head Protrusion | apoeb | HiC_scaffold_53 | LL |
| Maxillary Head Protrusion | apoh | HiC_scaffold_53 | LL |
| Maxillary Head Protrusion | aqp10 | HiC_scaffold_53 | LL |
| Maxillary Head Protrusion | arhgef1 | HiC_scaffold_53 | LL |
| Maxillary Head Protrusion | arid1a | HiC_scaffold_53 | LL |
| Maxillary Head Protrusion | arnt | HiC_scaffold_53 | LL |
| Maxillary Head Protrusion | atg12 | HiC_scaffold_53 | LL |
| Maxillary Head Protrusion | atp1a3 | HiC_scaffold_53 | LL |
| Maxillary Head Protrusion | atp6v1c1a | HiC_scaffold_53 | LL |
| Maxillary Head Protrusion | atp8b2 | HiC_scaffold_53 | LL |
| Maxillary Head Protrusion | atxn1 | HiC_scaffold_53 | LL |
| Maxillary Head Protrusion | azi2 | HiC_scaffold_53 | LL |
| Maxillary Head Protrusion | azin1 | HiC_scaffold_53 | LL |
| Maxillary Head Protrusion | baiap2 | HiC_scaffold_53 | LL |
| Maxillary Head Protrusion | bcam | HiC_scaffold_53 | LL |
| Maxillary Head Protrusion | bckdhb | HiC_scaffold_53 | LL |
| Maxillary Head Protrusion | bcl3 | HiC_scaffold_53 | LL |
| Maxillary Head Protrusion | brd2 | HiC_scaffold_53 | LL |
| Maxillary Head Protrusion | brd9 | HiC_scaffold_53 | LL |
| Maxillary Head Protrusion | btg4 | HiC_scaffold_53 | LL |
| Maxillary Head Protrusion | c1orf232 | HiC_scaffold_53 | LL |
| Maxillary Head Protrusion | c1ra | HiC_scaffold_53 | LL |
| Maxillary Head Protrusion | c3ar1 | HiC_scaffold_53 | LL |
| Maxillary Head Protrusion | c4 | HiC_scaffold_53 | LL |
| Maxillary Head Protrusion | c7orf31 | HiC_scaffold_53 | LL |
| Maxillary Head Protrusion | c8orf76 | HiC_scaffold_53 | LL |
| Maxillary Head Protrusion | c8orf88 | HiC_scaffold_53 | LL |
| Maxillary Head Protrusion | ca14 | HiC_scaffold_53 | LL |
| Maxillary Head Protrusion | cacng6 | HiC_scaffold_53 | LL |
| Maxillary Head Protrusion | cacng7 | HiC_scaffold_53 | LL |
| Maxillary Head Protrusion | cacng8 | HiC_scaffold_53 | LL |
| Maxillary Head Protrusion | carmil1 | HiC_scaffold_53 | LL |
| Maxillary Head Protrusion | casp2 | HiC_scaffold_53 | LL |
| Maxillary Head Protrusion | ccdc106 | HiC_scaffold_53 | LL |
| Maxillary Head Protrusion | ccr3 | HiC_scaffold_53 | LL |
| Maxillary Head Protrusion | ccr4 | HiC_scaffold_53 | LL |
| Maxillary Head Protrusion | ccr5 | HiC_scaffold_53 | LL |
| Maxillary Head Protrusion | cd22 | HiC_scaffold_53 | LL |
| Maxillary Head Protrusion | cd2ap | HiC_scaffold_53 | LL |
| Maxillary Head Protrusion | cd33 | HiC_scaffold_53 | LL |
| Maxillary Head Protrusion | cd4 | HiC_scaffold_53 | LL |
| Maxillary Head Protrusion | cd40 | HiC_scaffold_53 | LL |
| Maxillary Head Protrusion | cd79a | HiC_scaffold_53 | LL |
| Maxillary Head Protrusion | cdc42 | HiC_scaffold_53 | LL |
| Maxillary Head Protrusion | cdc42ep5 | HiC_scaffold_53 | LL |
| Maxillary Head Protrusion | cdca3 | HiC_scaffold_53 | LL |
| Maxillary Head Protrusion | cdh17 | HiC_scaffold_53 | LL |
| Maxillary Head Protrusion | ceacam1 | HiC_scaffold_53 | LL |
| Maxillary Head Protrusion | ceacam2 | HiC_scaffold_53 | LL |
| Maxillary Head Protrusion | ceacam5 | HiC_scaffold_53 | LL |
| Maxillary Head Protrusion | celf3 | HiC_scaffold_53 | LL |
| Maxillary Head Protrusion | cep72 | HiC_scaffold_53 | LL |
| Maxillary Head Protrusion | cers2 | HiC_scaffold_53 | LL |
| Maxillary Head Protrusion | cgn | HiC_scaffold_53 | LL |
| Maxillary Head Protrusion | chrna7 | HiC_scaffold_53 | LL |
| Maxillary Head Protrusion | chrnb2 | HiC_scaffold_53 | LL |
| Maxillary Head Protrusion | ciart | HiC_scaffold_53 | LL |
| Maxillary Head Protrusion | cic | HiC_scaffold_53 | LL |
| Maxillary Head Protrusion | cited3 | HiC_scaffold_53 | LL |
| Maxillary Head Protrusion | cldn12 | HiC_scaffold_53 | LL |
| Maxillary Head Protrusion | clec3b | HiC_scaffold_53 | LL |
| Maxillary Head Protrusion | clk2 | HiC_scaffold_53 | LL |
| Maxillary Head Protrusion | clptm1l | HiC_scaffold_53 | LL |
| Maxillary Head Protrusion | clstn3 | HiC_scaffold_53 | LL |
| Maxillary Head Protrusion | cmc1 | HiC_scaffold_53 | LL |
| Maxillary Head Protrusion | cmlkr1 | HiC_scaffold_53 | LL |
| Maxillary Head Protrusion | cnfn | HiC_scaffold_53 | LL |
| Maxillary Head Protrusion | cnot3 | HiC_scaffold_53 | LL |
| Maxillary Head Protrusion | cnp-1 | HiC_scaffold_53 | LL |
| Maxillary Head Protrusion | cnr2 | HiC_scaffold_53 | LL |
| Maxillary Head Protrusion | col14a1 | HiC_scaffold_53 | LL |
| Maxillary Head Protrusion | coq3 | HiC_scaffold_53 | LL |
| Maxillary Head Protrusion | cox6b1 | HiC_scaffold_53 | LL |
| Maxillary Head Protrusion | cpq | HiC_scaffold_53 | LL |
| Maxillary Head Protrusion | cpvl | HiC_scaffold_53 | LL |
| Maxillary Head Protrusion | crabp2 | HiC_scaffold_53 | LL |
| Maxillary Head Protrusion | creb5 | HiC_scaffold_53 | LL |
| Maxillary Head Protrusion | crot | HiC_scaffold_53 | LL |
| Maxillary Head Protrusion | CP | HiC_scaffold_53 | LL |
| Maxillary Head Protrusion | csmd3 | HiC_scaffold_53 | LL |
| Maxillary Head Protrusion | csnk2a1 | HiC_scaffold_53 | LL |
| Maxillary Head Protrusion | csnk2b | HiC_scaffold_53 | LL |
| Maxillary Head Protrusion | cspg5 | HiC_scaffold_53 | LL |
| Maxillary Head Protrusion | cthrc1 | HiC_scaffold_53 | LL |
| Maxillary Head Protrusion | ctrl | HiC_scaffold_53 | LL |
| Maxillary Head Protrusion | cxcr3 | HiC_scaffold_53 | LL |
| Maxillary Head Protrusion | cxcr3.2 | HiC_scaffold_53 | LL |
| Maxillary Head Protrusion | cxcr4-b | HiC_scaffold_53 | LL |
| Maxillary Head Protrusion | cyp21a2 | HiC_scaffold_53 | LL |
| Maxillary Head Protrusion | cyp4b1 | HiC_scaffold_53 | LL |
| Maxillary Head Protrusion | cyth2 | HiC_scaffold_53 | LL |
| Maxillary Head Protrusion | cyth3 | HiC_scaffold_53 | LL |
| Maxillary Head Protrusion | d215 | HiC_scaffold_53 | LL |
| Maxillary Head Protrusion | dbi | HiC_scaffold_53 | LL |
| Maxillary Head Protrusion | dcaf13 | HiC_scaffold_53 | LL |
| Maxillary Head Protrusion | dcbld1 | HiC_scaffold_53 | LL |
| Maxillary Head Protrusion | ddr1 | HiC_scaffold_53 | LL |
| Maxillary Head Protrusion | dedd2 | HiC_scaffold_53 | LL |
| Maxillary Head Protrusion | dennd3 | HiC_scaffold_53 | LL |
| Maxillary Head Protrusion | depdc1b | HiC_scaffold_53 | LL |
| Maxillary Head Protrusion | deptor | HiC_scaffold_53 | LL |
| Maxillary Head Protrusion | derl1 | HiC_scaffold_53 | LL |
| Maxillary Head Protrusion | dgat1 | HiC_scaffold_53 | LL |
| Maxillary Head Protrusion | dgkb | HiC_scaffold_53 | LL |
| Maxillary Head Protrusion | dnah11 | HiC_scaffold_53 | LL |
| Maxillary Head Protrusion | dnali1 | HiC_scaffold_53 | LL |
| Maxillary Head Protrusion | dop1a | HiC_scaffold_53 | LL |
| Maxillary Head Protrusion | dpy19l1 | HiC_scaffold_53 | LL |
| Maxillary Head Protrusion | dpys | HiC_scaffold_53 | LL |
| Maxillary Head Protrusion | dscc1 | HiC_scaffold_53 | LL |
| Maxillary Head Protrusion | e2f3 | HiC_scaffold_53 | LL |
| Maxillary Head Protrusion | ebag9 | HiC_scaffold_53 | LL |
| Maxillary Head Protrusion | ecm1 | HiC_scaffold_53 | LL |
| Maxillary Head Protrusion | edn1 | HiC_scaffold_53 | LL |
| Maxillary Head Protrusion | efhb | HiC_scaffold_53 | LL |
| Maxillary Head Protrusion | efna1 | HiC_scaffold_53 | LL |
| Maxillary Head Protrusion | efna3 | HiC_scaffold_53 | LL |
| Maxillary Head Protrusion | eif3e | HiC_scaffold_53 | LL |
| Maxillary Head Protrusion | emc2 | HiC_scaffold_53 | LL |
| Maxillary Head Protrusion | emg1 | HiC_scaffold_53 | LL |
| Maxillary Head Protrusion | entpd3 | HiC_scaffold_53 | LL |
| Maxillary Head Protrusion | epb41 | HiC_scaffold_53 | LL |
| Maxillary Head Protrusion | ephb5 | HiC_scaffold_53 | LL |
| Maxillary Head Protrusion | epn1 | HiC_scaffold_53 | LL |
| Maxillary Head Protrusion | erf | HiC_scaffold_53 | LL |
| Maxillary Head Protrusion | erp44 | HiC_scaffold_53 | LL |
| Maxillary Head Protrusion | esrp1 | HiC_scaffold_53 | LL |
| Maxillary Head Protrusion | etfb | HiC_scaffold_53 | LL |
| Maxillary Head Protrusion | ethe1 | HiC_scaffold_53 | LL |
| Maxillary Head Protrusion | etv1 | HiC_scaffold_53 | LL |
| Maxillary Head Protrusion | eva1b | HiC_scaffold_53 | LL |
| Maxillary Head Protrusion | fabp4 | HiC_scaffold_53 | LL |
| Maxillary Head Protrusion | fam110c | HiC_scaffold_53 | LL |
| Maxillary Head Protrusion | fam131b | HiC_scaffold_53 | LL |
| Maxillary Head Protrusion | fam189b | HiC_scaffold_53 | LL |
| Maxillary Head Protrusion | fam83a | HiC_scaffold_53 | LL |
| Maxillary Head Protrusion | fam8a1 | HiC_scaffold_53 | LL |
| Maxillary Head Protrusion | faxc | HiC_scaffold_53 | LL |
| Maxillary Head Protrusion | fbxl4 | HiC_scaffold_53 | LL |
| Maxillary Head Protrusion | fbxw7 | HiC_scaffold_53 | LL |
| Maxillary Head Protrusion | fcgbp | HiC_scaffold_53 | LL |
| Maxillary Head Protrusion | fdps | HiC_scaffold_53 | LL |
| Maxillary Head Protrusion | fez2 | HiC_scaffold_53 | LL |
| Maxillary Head Protrusion | ffar2 | HiC_scaffold_53 | LL |
| Maxillary Head Protrusion | ffar3 | HiC_scaffold_53 | LL |
| Maxillary Head Protrusion | fhl3 | HiC_scaffold_53 | LL |
| Maxillary Head Protrusion | fhod3 | HiC_scaffold_53 | LL |
| Maxillary Head Protrusion | fkbp14 | HiC_scaffold_53 | LL |
| Maxillary Head Protrusion | flcn | HiC_scaffold_53 | LL |
| Maxillary Head Protrusion | flot1 | HiC_scaffold_53 | LL |
| Maxillary Head Protrusion | foxj2 | HiC_scaffold_53 | LL |
| Maxillary Head Protrusion | foxo3 | HiC_scaffold_53 | LL |
| Maxillary Head Protrusion | frrs1l | HiC_scaffold_53 | LL |
| Maxillary Head Protrusion | fsbp | HiC_scaffold_53 | LL |
| Maxillary Head Protrusion | fxyd1 | HiC_scaffold_53 | LL |
| Maxillary Head Protrusion | fzd6 | HiC_scaffold_53 | LL |
| Maxillary Head Protrusion | galnt1 | HiC_scaffold_53 | LL |
| Maxillary Head Protrusion | galr1 | HiC_scaffold_53 | LL |
| Maxillary Head Protrusion | gapdh | HiC_scaffold_53 | LL |
| Maxillary Head Protrusion | gba | HiC_scaffold_53 | LL |
| Maxillary Head Protrusion | gdf6a | HiC_scaffold_53 | LL |
| Maxillary Head Protrusion | gem | HiC_scaffold_53 | LL |
| Maxillary Head Protrusion | glcci1 | HiC_scaffold_53 | LL |
| Maxillary Head Protrusion | glipr2 | HiC_scaffold_53 | LL |
| Maxillary Head Protrusion | gmeb1 | HiC_scaffold_53 | LL |
| Maxillary Head Protrusion | gnb3 | HiC_scaffold_53 | LL |
| Maxillary Head Protrusion | gnl2 | HiC_scaffold_53 | LL |
| Maxillary Head Protrusion | gpatch3 | HiC_scaffold_53 | LL |
| Maxillary Head Protrusion | gpn2 | HiC_scaffold_53 | LL |
| Maxillary Head Protrusion | gpr20 | HiC_scaffold_53 | LL |
| Maxillary Head Protrusion | gpr42 | HiC_scaffold_53 | LL |
| Maxillary Head Protrusion | grb10 | HiC_scaffold_53 | LL |
| Maxillary Head Protrusion | grik3 | HiC_scaffold_53 | LL |
| Maxillary Head Protrusion | grik5 | HiC_scaffold_53 | LL |
| Maxillary Head Protrusion | grina | HiC_scaffold_53 | LL |
| Maxillary Head Protrusion | grwd1 | HiC_scaffold_53 | LL |
| Maxillary Head Protrusion | gsdme | HiC_scaffold_53 | LL |
| Maxillary Head Protrusion | gsg1l | HiC_scaffold_53 | LL |
| Maxillary Head Protrusion | gsk3a | HiC_scaffold_53 | LL |
| Maxillary Head Protrusion | gstk1 | HiC_scaffold_53 | LL |
| Maxillary Head Protrusion | gtpbp10 | HiC_scaffold_53 | LL |
| Maxillary Head Protrusion | h2-eb1 | HiC_scaffold_53 | LL |
| Maxillary Head Protrusion | hacl1 | HiC_scaffold_53 | LL |
| Maxillary Head Protrusion | hamp | HiC_scaffold_53 | LL |
| Maxillary Head Protrusion | hamp1 | HiC_scaffold_53 | LL |
| Maxillary Head Protrusion | has1 | HiC_scaffold_53 | LL |
| Maxillary Head Protrusion | has2 | HiC_scaffold_53 | LL |
| Maxillary Head Protrusion | hcn4 | HiC_scaffold_53 | LL |
| Maxillary Head Protrusion | hdac9b | HiC_scaffold_53 | LL |
| Maxillary Head Protrusion | hepacam2 | HiC_scaffold_53 | LL |
| Maxillary Head Protrusion | herpud2 | HiC_scaffold_53 | LL |
| Maxillary Head Protrusion | hey1 | HiC_scaffold_53 | LL |
| Maxillary Head Protrusion | hibadh | HiC_scaffold_53 | LL |
| Maxillary Head Protrusion | hivep3 | HiC_scaffold_53 | LL |
| Maxillary Head Protrusion | hlf | HiC_scaffold_53 | LL |
| Maxillary Head Protrusion | hoxa10b | HiC_scaffold_53 | LL |
| Maxillary Head Protrusion | hoxa11b | HiC_scaffold_53 | LL |
| Maxillary Head Protrusion | hoxa9b | HiC_scaffold_53 | LL |
| Maxillary Head Protrusion | hspa8 | HiC_scaffold_53 | LL |
| Maxillary Head Protrusion | hspb6 | HiC_scaffold_53 | LL |
| Maxillary Head Protrusion | iffo1 | HiC_scaffold_53 | LL |
| Maxillary Head Protrusion | iglon5 | HiC_scaffold_53 | LL |
| Maxillary Head Protrusion | il16 | HiC_scaffold_53 | LL |
| Maxillary Head Protrusion | il20ra | HiC_scaffold_53 | LL |
| Maxillary Head Protrusion | il6r | HiC_scaffold_53 | LL |
| Maxillary Head Protrusion | ing4 | HiC_scaffold_53 | LL |
| Maxillary Head Protrusion | ino80c | HiC_scaffold_53 | LL |
| Maxillary Head Protrusion | invs | HiC_scaffold_53 | LL |
| Maxillary Head Protrusion | iqcg | HiC_scaffold_53 | LL |
| Maxillary Head Protrusion | irgc | HiC_scaffold_53 | LL |
| Maxillary Head Protrusion | irx2 | HiC_scaffold_53 | LL |
| Maxillary Head Protrusion | irx4 | HiC_scaffold_53 | LL |
| Maxillary Head Protrusion | isoc2 | HiC_scaffold_53 | LL |
| Maxillary Head Protrusion | ispd | HiC_scaffold_53 | LL |
| Maxillary Head Protrusion | itga10 | HiC_scaffold_53 | LL |
| Maxillary Head Protrusion | jazf1 | HiC_scaffold_53 | LL |
| Maxillary Head Protrusion | josd2 | HiC_scaffold_53 | LL |
| Maxillary Head Protrusion | kcnj2 | HiC_scaffold_53 | LL |
| Maxillary Head Protrusion | kcnk5 | HiC_scaffold_53 | LL |
| Maxillary Head Protrusion | kdf1 | HiC_scaffold_53 | LL |
| Maxillary Head Protrusion | khdc4 | HiC_scaffold_53 | LL |
| Maxillary Head Protrusion | kirrel1 | HiC_scaffold_53 | LL |
| Maxillary Head Protrusion | klf10 | HiC_scaffold_53 | LL |
| Maxillary Head Protrusion | krtcap2 | HiC_scaffold_53 | LL |
| Maxillary Head Protrusion | lag3 | HiC_scaffold_53 | LL |
| Maxillary Head Protrusion | laptm4b | HiC_scaffold_53 | LL |
| Maxillary Head Protrusion | lars2 | HiC_scaffold_53 | LL |
| Maxillary Head Protrusion | lenep | HiC_scaffold_53 | LL |
| Maxillary Head Protrusion | leng1 | HiC_scaffold_53 | LL |
| Maxillary Head Protrusion | leng8 | HiC_scaffold_53 | LL |
| Maxillary Head Protrusion | leng9 | HiC_scaffold_53 | LL |
| Maxillary Head Protrusion | lim2 | HiC_scaffold_53 | LL |
| Maxillary Head Protrusion | limd1 | HiC_scaffold_53 | LL |
| Maxillary Head Protrusion | lin37 | HiC_scaffold_53 | LL |
| Maxillary Head Protrusion | lipe | HiC_scaffold_53 | LL |
| Maxillary Head Protrusion | lmtk3 | HiC_scaffold_53 | LL |
| Maxillary Head Protrusion | lpcat1 | HiC_scaffold_53 | LL |
| Maxillary Head Protrusion | lsr | HiC_scaffold_53 | LL |
| Maxillary Head Protrusion | m6pr | HiC_scaffold_53 | LL |
| Maxillary Head Protrusion | macc1 | HiC_scaffold_53 | LL |
| Maxillary Head Protrusion | mag | HiC_scaffold_53 | LL |
| Maxillary Head Protrusion | maip1 | HiC_scaffold_53 | LL |
| Maxillary Head Protrusion | mal2 | HiC_scaffold_53 | LL |
| Maxillary Head Protrusion | malsu1 | HiC_scaffold_53 | LL |
| Maxillary Head Protrusion | mamu-dra | HiC_scaffold_53 | LL |
| Maxillary Head Protrusion | man1c1 | HiC_scaffold_53 | LL |
| Maxillary Head Protrusion | maneal | HiC_scaffold_53 | LL |
| Maxillary Head Protrusion | map7d1 | HiC_scaffold_53 | LL |
| Maxillary Head Protrusion | matn4 | HiC_scaffold_53 | LL |
| Maxillary Head Protrusion | mboat1 | HiC_scaffold_53 | LL |
| Maxillary Head Protrusion | mboat7 | HiC_scaffold_53 | LL |
| Maxillary Head Protrusion | mbp | HiC_scaffold_53 | LL |
| Maxillary Head Protrusion | mcam | HiC_scaffold_53 | LL |
| Maxillary Head Protrusion | mdc1 | HiC_scaffold_53 | LL |
| Maxillary Head Protrusion | me1 | HiC_scaffold_53 | LL |
| Maxillary Head Protrusion | mecr | HiC_scaffold_53 | LL |
| Maxillary Head Protrusion | med18 | HiC_scaffold_53 | LL |
| Maxillary Head Protrusion | megf8 | HiC_scaffold_53 | LL |
| Maxillary Head Protrusion | meox2 | HiC_scaffold_53 | LL |
| Maxillary Head Protrusion | mep1b | HiC_scaffold_53 | LL |
| Maxillary Head Protrusion | mindy3 | HiC_scaffold_53 | LL |
| Maxillary Head Protrusion | mios | HiC_scaffold_53 | LL |
| Maxillary Head Protrusion | mlf2 | HiC_scaffold_53 | LL |
| Maxillary Head Protrusion | mms22l | HiC_scaffold_53 | LL |
| Maxillary Head Protrusion | mpp6 | HiC_scaffold_53 | LL |
| Maxillary Head Protrusion | mpra | HiC_scaffold_53 | LL |
| Maxillary Head Protrusion | mpv17l | HiC_scaffold_53 | LL |
| Maxillary Head Protrusion | mrc1 | HiC_scaffold_53 | LL |
| Maxillary Head Protrusion | mrpl13 | HiC_scaffold_53 | LL |
| Maxillary Head Protrusion | mrpl17 | HiC_scaffold_53 | LL |
| Maxillary Head Protrusion | mrpl51 | HiC_scaffold_53 | LL |
| Maxillary Head Protrusion | mrps15 | HiC_scaffold_53 | LL |
| Maxillary Head Protrusion | mrs2 | HiC_scaffold_53 | LL |
| Maxillary Head Protrusion | mrtfb | HiC_scaffold_53 | LL |
| Maxillary Head Protrusion | msh5 | HiC_scaffold_53 | LL |
| Maxillary Head Protrusion | mtdh | HiC_scaffold_53 | LL |
| Maxillary Head Protrusion | mtf1 | HiC_scaffold_53 | LL |
| Maxillary Head Protrusion | mtss1 | HiC_scaffold_53 | LL |
| Maxillary Head Protrusion | mycbp | HiC_scaffold_53 | LL |
| Maxillary Head Protrusion | myh10 | HiC_scaffold_53 | LL |
| Maxillary Head Protrusion | mypop | HiC_scaffold_53 | LL |
| Maxillary Head Protrusion | nacad | HiC_scaffold_53 | LL |
| Maxillary Head Protrusion | nat14 | HiC_scaffold_53 | LL |
| Maxillary Head Protrusion | ncapd2 | HiC_scaffold_53 | LL |
| Maxillary Head Protrusion | ndufa3 | HiC_scaffold_53 | LL |
| Maxillary Head Protrusion | ndufv1 | HiC_scaffold_53 | LL |
| Maxillary Head Protrusion | necap1 | HiC_scaffold_53 | LL |
| Maxillary Head Protrusion | nectin1 | HiC_scaffold_53 | LL |
| Maxillary Head Protrusion | nectin2 | HiC_scaffold_53 | LL |
| Maxillary Head Protrusion | nek10 | HiC_scaffold_53 | LL |
| Maxillary Head Protrusion | nfatc1 | HiC_scaffold_53 | LL |
| Maxillary Head Protrusion | ngfr | HiC_scaffold_53 | LL |
| Maxillary Head Protrusion | nhlrc1 | HiC_scaffold_53 | LL |
| Maxillary Head Protrusion | nkd2l | HiC_scaffold_53 | LL |
| Maxillary Head Protrusion | nmt2 | HiC_scaffold_53 | LL |
| Maxillary Head Protrusion | nop2 | HiC_scaffold_53 | LL |
| Maxillary Head Protrusion | notch1 | HiC_scaffold_53 | LL |
| Maxillary Head Protrusion | nov | HiC_scaffold_53 | LL |
| Maxillary Head Protrusion | nphs1 | HiC_scaffold_53 | LL |
| Maxillary Head Protrusion | nptx2 | HiC_scaffold_53 | LL |
| Maxillary Head Protrusion | nr0b2 | HiC_scaffold_53 | LL |
| Maxillary Head Protrusion | nr1d2 | HiC_scaffold_53 | LL |
| Maxillary Head Protrusion | nr4a3 | HiC_scaffold_53 | LL |
| Maxillary Head Protrusion | nrsn1 | HiC_scaffold_53 | LL |
| Maxillary Head Protrusion | nxph1 | HiC_scaffold_53 | LL |
| Maxillary Head Protrusion | oprd1 | HiC_scaffold_53 | LL |
| Maxillary Head Protrusion | osbpl3 | HiC_scaffold_53 | LL |
| Maxillary Head Protrusion | oscp1 | HiC_scaffold_53 | LL |
| Maxillary Head Protrusion | osgin2 | HiC_scaffold_53 | LL |
| Maxillary Head Protrusion | oxr1 | HiC_scaffold_53 | LL |
| Maxillary Head Protrusion | p3h3 | HiC_scaffold_53 | LL |
| Maxillary Head Protrusion | pafah1b3 | HiC_scaffold_53 | LL |
| Maxillary Head Protrusion | pde11a | HiC_scaffold_53 | LL |
| Maxillary Head Protrusion | pdp1 | HiC_scaffold_53 | LL |
| Maxillary Head Protrusion | pex5 | HiC_scaffold_53 | LL |
| Maxillary Head Protrusion | phactr4b | HiC_scaffold_53 | LL |
| Maxillary Head Protrusion | phc1 | HiC_scaffold_53 | LL |
| Maxillary Head Protrusion | phldb3 | HiC_scaffold_53 | LL |
| Maxillary Head Protrusion | pigv | HiC_scaffold_53 | LL |
| Maxillary Head Protrusion | pim2 | HiC_scaffold_53 | LL |
| Maxillary Head Protrusion | pip5k1a | HiC_scaffold_53 | LL |
| Maxillary Head Protrusion | pitpnc1 | HiC_scaffold_53 | LL |
| Maxillary Head Protrusion | plcl2 | HiC_scaffold_53 | LL |
| Maxillary Head Protrusion | plec | HiC_scaffold_53 | LL |
| Maxillary Head Protrusion | plekhg4b | HiC_scaffold_53 | LL |
| Maxillary Head Protrusion | plekhg6 | HiC_scaffold_53 | LL |
| Maxillary Head Protrusion | pnisr | HiC_scaffold_53 | LL |
| Maxillary Head Protrusion | pon2 | HiC_scaffold_53 | LL |
| Maxillary Head Protrusion | pop1 | HiC_scaffold_53 | LL |
| Maxillary Head Protrusion | pou2f2 | HiC_scaffold_53 | LL |
| Maxillary Head Protrusion | pou3f1 | HiC_scaffold_53 | LL |
| Maxillary Head Protrusion | pou3f2 | HiC_scaffold_53 | LL |
| Maxillary Head Protrusion | ppp1r8 | HiC_scaffold_53 | LL |
| Maxillary Head Protrusion | prdm9 | HiC_scaffold_53 | LL |
| Maxillary Head Protrusion | prkca | HiC_scaffold_53 | LL |
| Maxillary Head Protrusion | proser3 | HiC_scaffold_53 | LL |
| Maxillary Head Protrusion | prpf3 | HiC_scaffold_53 | LL |
| Maxillary Head Protrusion | prpf31 | HiC_scaffold_53 | LL |
| Maxillary Head Protrusion | prss35 | HiC_scaffold_53 | LL |
| Maxillary Head Protrusion | psenen | HiC_scaffold_53 | LL |
| Maxillary Head Protrusion | psmd4 | HiC_scaffold_53 | LL |
| Maxillary Head Protrusion | ptdss1 | HiC_scaffold_53 | LL |
| Maxillary Head Protrusion | ptk2 | HiC_scaffold_53 | LL |
| Maxillary Head Protrusion | ptp4a3 | HiC_scaffold_53 | LL |
| Maxillary Head Protrusion | ptpn3 | HiC_scaffold_53 | LL |
| Maxillary Head Protrusion | ptpn6 | HiC_scaffold_53 | LL |
| Maxillary Head Protrusion | ptpru | HiC_scaffold_53 | LL |
| Maxillary Head Protrusion | ptx2 | HiC_scaffold_53 | LL |
| Maxillary Head Protrusion | pxn1 | HiC_scaffold_53 | LL |
| Maxillary Head Protrusion | rab5a | HiC_scaffold_53 | LL |
| Maxillary Head Protrusion | rabac1 | HiC_scaffold_53 | LL |
| Maxillary Head Protrusion | racgap1 | HiC_scaffold_53 | LL |
| Maxillary Head Protrusion | rad54b | HiC_scaffold_53 | LL |
| Maxillary Head Protrusion | rapgef5 | HiC_scaffold_53 | LL |
| Maxillary Head Protrusion | rarb | HiC_scaffold_53 | LL |
| Maxillary Head Protrusion | rasip1 | HiC_scaffold_53 | LL |
| Maxillary Head Protrusion | rbfox1l | HiC_scaffold_53 | LL |
| Maxillary Head Protrusion | rbm12b | HiC_scaffold_53 | LL |
| Maxillary Head Protrusion | rbm24 | HiC_scaffold_53 | LL |
| Maxillary Head Protrusion | rbp1 | HiC_scaffold_53 | LL |
| Maxillary Head Protrusion | rcc1 | HiC_scaffold_53 | LL |
| Maxillary Head Protrusion | rec8 | HiC_scaffold_53 | LL |
| Maxillary Head Protrusion | rft2 | HiC_scaffold_53 | LL |
| Maxillary Head Protrusion | rhbdl2 | HiC_scaffold_53 | LL |
| Maxillary Head Protrusion | rims2 | HiC_scaffold_53 | LL |
| Maxillary Head Protrusion | rnf144b | HiC_scaffold_53 | LL |
| Maxillary Head Protrusion | rnf41 | HiC_scaffold_53 | LL |
| Maxillary Head Protrusion | rpa2 | HiC_scaffold_53 | LL |
| Maxillary Head Protrusion | rpp38 | HiC_scaffold_53 | LL |
| Maxillary Head Protrusion | rps19 | HiC_scaffold_53 | LL |
| Maxillary Head Protrusion | rps27l | HiC_scaffold_53 | LL |
| Maxillary Head Protrusion | rps9 | HiC_scaffold_53 | LL |
| Maxillary Head Protrusion | rragc | HiC_scaffold_53 | LL |
| Maxillary Head Protrusion | rsph4a | HiC_scaffold_53 | LL |
| Maxillary Head Protrusion | rspo1 | HiC_scaffold_53 | LL |
| Maxillary Head Protrusion | rt1-b | HiC_scaffold_53 | LL |
| Maxillary Head Protrusion | rundc3b | HiC_scaffold_53 | LL |
| Maxillary Head Protrusion | rusc1 | HiC_scaffold_53 | LL |
| Maxillary Head Protrusion | rxrba | HiC_scaffold_53 | LL |
| Maxillary Head Protrusion | s100a1 | HiC_scaffold_53 | LL |
| Maxillary Head Protrusion | s100a16 | HiC_scaffold_53 | LL |
| Maxillary Head Protrusion | s100a6 | HiC_scaffold_53 | LL |
| Maxillary Head Protrusion | s100g | HiC_scaffold_53 | LL |
| Maxillary Head Protrusion | sall3 | HiC_scaffold_53 | LL |
| Maxillary Head Protrusion | sbk2 | HiC_scaffold_53 | LL |
| Maxillary Head Protrusion | scgn | HiC_scaffold_53 | LL |
| Maxillary Head Protrusion | scrt2 | HiC_scaffold_53 | LL |
| Maxillary Head Protrusion | sdc2-b | HiC_scaffold_53 | LL |
| Maxillary Head Protrusion | serinc1 | HiC_scaffold_53 | LL |
| Maxillary Head Protrusion | setdb1b | HiC_scaffold_53 | LL |
| Maxillary Head Protrusion | shc1 | HiC_scaffold_53 | LL |
| Maxillary Head Protrusion | she | HiC_scaffold_53 | LL |
| Maxillary Head Protrusion | shisa7 | HiC_scaffold_53 | LL |
| Maxillary Head Protrusion | siglec1 | HiC_scaffold_53 | LL |
| Maxillary Head Protrusion | siglec14 | HiC_scaffold_53 | LL |
| Maxillary Head Protrusion | siglec5 | HiC_scaffold_53 | LL |
| Maxillary Head Protrusion | slc25a32 | HiC_scaffold_53 | LL |
| Maxillary Head Protrusion | slc25a40 | HiC_scaffold_53 | LL |
| Maxillary Head Protrusion | slc2a3 | HiC_scaffold_53 | LL |
| Maxillary Head Protrusion | slc40a1 | HiC_scaffold_53 | LL |
| Maxillary Head Protrusion | slc50a1 | HiC_scaffold_53 | LL |
| Maxillary Head Protrusion | slc6a3 | HiC_scaffold_53 | LL |
| Maxillary Head Protrusion | smarcc1 | HiC_scaffold_53 | LL |
| Maxillary Head Protrusion | smg9 | HiC_scaffold_53 | LL |
| Maxillary Head Protrusion | smp | HiC_scaffold_53 | LL |
| Maxillary Head Protrusion | smpd5 | HiC_scaffold_53 | LL |
| Maxillary Head Protrusion | snip1 | HiC_scaffold_53 | LL |
| Maxillary Head Protrusion | snx27 | HiC_scaffold_53 | LL |
| Maxillary Head Protrusion | sostdc1 | HiC_scaffold_53 | LL |
| Maxillary Head Protrusion | sox11 | HiC_scaffold_53 | LL |
| Maxillary Head Protrusion | sp8b | HiC_scaffold_53 | LL |
| Maxillary Head Protrusion | spaca6 | HiC_scaffold_53 | LL |
| Maxillary Head Protrusion | sphk2 | HiC_scaffold_53 | LL |
| Maxillary Head Protrusion | spire1 | HiC_scaffold_53 | LL |
| Maxillary Head Protrusion | spsb1 | HiC_scaffold_53 | LL |
| Maxillary Head Protrusion | sqle | HiC_scaffold_53 | LL |
| Maxillary Head Protrusion | st14 | HiC_scaffold_53 | LL |
| Maxillary Head Protrusion | steap4 | HiC_scaffold_53 | LL |
| Maxillary Head Protrusion | stk40 | HiC_scaffold_53 | LL |
| Maxillary Head Protrusion | stmn2 | HiC_scaffold_53 | LL |
| Maxillary Head Protrusion | sybu | HiC_scaffold_53 | LL |
| Maxillary Head Protrusion | taf12 | HiC_scaffold_53 | LL |
| Maxillary Head Protrusion | tap1 | HiC_scaffold_53 | LL |
| Maxillary Head Protrusion | tarsl2 | HiC_scaffold_53 | LL |
| Maxillary Head Protrusion | tbc1d15 | HiC_scaffold_53 | LL |
| Maxillary Head Protrusion | tbc1d31 | HiC_scaffold_53 | LL |
| Maxillary Head Protrusion | tbrg4 | HiC_scaffold_53 | LL |
| Maxillary Head Protrusion | tcea3 | HiC_scaffold_53 | LL |
| Maxillary Head Protrusion | tent4a | HiC_scaffold_53 | LL |
| Maxillary Head Protrusion | tent5a | HiC_scaffold_53 | LL |
| Maxillary Head Protrusion | tex10 | HiC_scaffold_53 | LL |
| Maxillary Head Protrusion | tfpt | HiC_scaffold_53 | LL |
| Maxillary Head Protrusion | them4 | HiC_scaffold_53 | LL |
| Maxillary Head Protrusion | themis2 | HiC_scaffold_53 | LL |
| Maxillary Head Protrusion | thsd4 | HiC_scaffold_53 | LL |
| Maxillary Head Protrusion | tlr13 | HiC_scaffold_53 | LL |
| Maxillary Head Protrusion | tmc7 | HiC_scaffold_53 | LL |
| Maxillary Head Protrusion | tmem106b | HiC_scaffold_53 | LL |
| Maxillary Head Protrusion | tmem107 | HiC_scaffold_53 | LL |
| Maxillary Head Protrusion | tmem158 | HiC_scaffold_53 | LL |
| Maxillary Head Protrusion | tmem222 | HiC_scaffold_53 | LL |
| Maxillary Head Protrusion | tmem238 | HiC_scaffold_53 | LL |
| Maxillary Head Protrusion | tmem244 | HiC_scaffold_53 | LL |
| Maxillary Head Protrusion | tmem245 | HiC_scaffold_53 | LL |
| Maxillary Head Protrusion | tmem65 | HiC_scaffold_53 | LL |
| Maxillary Head Protrusion | tmem67 | HiC_scaffold_53 | LL |
| Maxillary Head Protrusion | tmem74 | HiC_scaffold_53 | LL |
| Maxillary Head Protrusion | tmprss6 | HiC_scaffold_53 | LL |
| Maxillary Head Protrusion | tnc | HiC_scaffold_53 | LL |
| Maxillary Head Protrusion | tnfrsf11b | HiC_scaffold_53 | LL |
| Maxillary Head Protrusion | tnfrsf1a | HiC_scaffold_53 | LL |
| Maxillary Head Protrusion | tnfrsf6b | HiC_scaffold_53 | LL |
| Maxillary Head Protrusion | tpbg | HiC_scaffold_53 | LL |
| Maxillary Head Protrusion | tpi1b | HiC_scaffold_53 | LL |
| Maxillary Head Protrusion | tppp | HiC_scaffold_53 | LL |
| Maxillary Head Protrusion | trhr | HiC_scaffold_53 | LL |
| Maxillary Head Protrusion | trim16 | HiC_scaffold_53 | LL |
| Maxillary Head Protrusion | trim25 | HiC_scaffold_53 | LL |
| Maxillary Head Protrusion | trim46 | HiC_scaffold_53 | LL |
| Maxillary Head Protrusion | trio | HiC_scaffold_53 | LL |
| Maxillary Head Protrusion | trip13 | HiC_scaffold_53 | LL |
| Maxillary Head Protrusion | trp3 | HiC_scaffold_53 | LL |
| Maxillary Head Protrusion | trpv5 | HiC_scaffold_53 | LL |
| Maxillary Head Protrusion | tshz1 | HiC_scaffold_53 | LL |
| Maxillary Head Protrusion | tstd3 | HiC_scaffold_53 | LL |
| Maxillary Head Protrusion | ttk | HiC_scaffold_53 | LL |
| Maxillary Head Protrusion | ttyh1 | HiC_scaffold_53 | LL |
| Maxillary Head Protrusion | tuft1 | HiC_scaffold_53 | LL |
| Maxillary Head Protrusion | twist1 | HiC_scaffold_53 | LL |
| Maxillary Head Protrusion | tyrobp | HiC_scaffold_53 | LL |
| Maxillary Head Protrusion | ube2s | HiC_scaffold_53 | LL |
| Maxillary Head Protrusion | ubxn11 | HiC_scaffold_53 | LL |
| Maxillary Head Protrusion | upp1 | HiC_scaffold_53 | LL |
| Maxillary Head Protrusion | usf2 | HiC_scaffold_53 | LL |
| Maxillary Head Protrusion | utp11 | HiC_scaffold_53 | LL |
| Maxillary Head Protrusion | uts2r | HiC_scaffold_53 | LL |
| Maxillary Head Protrusion | vamp1 | HiC_scaffold_53 | LL |
| Maxillary Head Protrusion | vars | HiC_scaffold_53 | LL |
| Maxillary Head Protrusion | vim | HiC_scaffold_53 | LL |
| Maxillary Head Protrusion | virma | HiC_scaffold_53 | LL |
| Maxillary Head Protrusion | vmo1 | HiC_scaffold_53 | LL |
| Maxillary Head Protrusion | vsig10l | HiC_scaffold_53 | LL |
| Maxillary Head Protrusion | vwde | HiC_scaffold_53 | LL |
| Maxillary Head Protrusion | wdtc1 | HiC_scaffold_53 | LL |
| Maxillary Head Protrusion | wnt4 | HiC_scaffold_53 | LL |
| Maxillary Head Protrusion | xrcc1 | HiC_scaffold_53 | LL |
| Maxillary Head Protrusion | yqjl | HiC_scaffold_53 | LL |
| Maxillary Head Protrusion | yrdc | HiC_scaffold_53 | LL |
| Maxillary Head Protrusion | zadh2 | HiC_scaffold_53 | LL |
| Maxillary Head Protrusion | zbtb7b | HiC_scaffold_53 | LL |
| Maxillary Head Protrusion | zc3h12a | HiC_scaffold_53 | LL |
| Maxillary Head Protrusion | zdhhc11 | HiC_scaffold_53 | LL |
| Maxillary Head Protrusion | zdhhc18 | HiC_scaffold_53 | LL |
| Maxillary Head Protrusion | zhx2 | HiC_scaffold_53 | LL |
| Maxillary Head Protrusion | znf208 | HiC_scaffold_53 | LL |
| Maxillary Head Protrusion | znf226 | HiC_scaffold_53 | LL |
| Maxillary Head Protrusion | znf236 | HiC_scaffold_53 | LL |
| Maxillary Head Protrusion | znf282 | HiC_scaffold_53 | LL |
| Maxillary Head Protrusion | znf385d | HiC_scaffold_53 | LL |
| Maxillary Head Protrusion | znf436 | HiC_scaffold_53 | LL |
| Maxillary Head Protrusion | znf516 | HiC_scaffold_53 | LL |
| Maxillary Head Protrusion | znf524 | HiC_scaffold_53 | LL |
| Maxillary Head Protrusion | znf574 | HiC_scaffold_53 | LL |
| Maxillary Head Protrusion | znf585b | HiC_scaffold_53 | LL |
| Maxillary Head Protrusion | znf628 | HiC_scaffold_53 | LL |
| Maxillary Head Protrusion | znf687a | HiC_scaffold_53 | LL |
| Maxillary Head Protrusion | znf737 | HiC_scaffold_53 | LL |
| Maxillary Head Protrusion | znf79 | HiC_scaffold_53 | LL |
| Maxillary Head Protrusion | znf865 | HiC_scaffold_53 | LL |
